# Predisposition to Cancer Caused by Genetic and Functional Defects of Mammalian *Atad5*

**DOI:** 10.1371/journal.pgen.1002245

**Published:** 2011-08-25

**Authors:** Daphne W. Bell, Nilabja Sikdar, Kyoo-young Lee, Jessica C. Price, Raghunath Chatterjee, Hee-Dong Park, Jennifer Fox, Masamichi Ishiai, Meghan L. Rudd, Lana M. Pollock, Sarah K. Fogoros, Hassan Mohamed, Christin L. Hanigan, NISC Comparative Sequencing Program, Suiyuan Zhang, Pedro Cruz, Gabriel Renaud, Nancy F. Hansen, Praveen F. Cherukuri, Bhavesh Borate, Kirk J. McManus, Jan Stoepel, Payal Sipahimalani, Andrew K. Godwin, Dennis C. Sgroi, Maria J. Merino, Gene Elliot, Abdel Elkahloun, Charles Vinson, Minoru Takata, James C. Mullikin, Tyra G. Wolfsberg, Philip Hieter, Dae-Sik Lim, Kyungjae Myung

**Affiliations:** 1 Cancer Genetics Branch, National Human Genome Research Institute, National Institutes of Health, Bethesda, Maryland, United States of America; 2 Genetics and Molecular Biology Branch, National Human Genome Research Institute, National Institutes of Health, Bethesda, Maryland, United States of America; 3 Center for Cancer Research, National Cancer Institute, National Institutes of Health, Bethesda, Maryland, United States of America; 4 National Research Laboratory for Genomic Stability, Department of Biological Sciences, Korea Advanced Institute of Science and Technology, Daejeon, Korea; 5 Laboratory of DNA Damage Signaling, Department of Late Effect Studies, Radiation Biology Center, Kyoto University, Yoshidakonoe-cho, Sakyo-ku, Kyoto, Japan; 6 NIH Intramural Sequencing Center, National Institutes of Health, Bethesda, Maryland, United States of America; 7 Genome Technology Branch, National Human Genome Research Institute, National Institutes of Health, Bethesda, Maryland, United States of America; 8 Michael Smith Laboratories, University of British Columbia, Vancouver, Canada; 9 Department of Pathology and Laboratory Medicine, University of Kansas Medical Center, Kansas City, Kansas, United States of America; 10 Molecular Pathology Unit and Center for Cancer Research, Massachusetts General Hospital, Charlestown, Massachusetts, United States of America; 11 Genetic Disease Research Branch, National Human Genome Research Institute, National Institutes of Health, Bethesda, Maryland, United States of America; University of Washington, United States of America

## Abstract

ATAD5, the human ortholog of yeast Elg1, plays a role in PCNA deubiquitination. Since PCNA modification is important to regulate DNA damage bypass, ATAD5 may be important for suppression of genomic instability in mammals *in vivo*. To test this hypothesis, we generated heterozygous (*Atad5^+/m^*) mice that were haploinsuffficient for Atad5. *Atad5^+/m^* mice displayed high levels of genomic instability *in vivo*, and *Atad5^+/m^* mouse embryonic fibroblasts (MEFs) exhibited molecular defects in PCNA deubiquitination in response to DNA damage, as well as DNA damage hypersensitivity and high levels of genomic instability, apoptosis, and aneuploidy. Importantly, 90% of haploinsufficient *Atad5^+/m^* mice developed tumors, including sarcomas, carcinomas, and adenocarcinomas, between 11 and 20 months of age. High levels of genomic alterations were evident in tumors that arose in the *Atad5^+/m^* mice. Consistent with a role for *Atad5* in suppressing tumorigenesis, we also identified somatic mutations of *ATAD5* in 4.6% of sporadic human endometrial tumors, including two nonsense mutations that resulted in loss of proper ATAD5 function. Taken together, our findings indicate that loss-of-function mutations in mammalian *Atad5* are sufficient to cause genomic instability and tumorigenesis.

## Author Summary

Genomic instability is a hallmark of tumorigenesis, suggesting that mutations in genes suppressing genomic instability contribute to this phenotype. In this study, we demonstrate for the first time that haploinsufficiency for Atad5, a protein that is important in stabilizing stalled DNA replication forks by regulating PCNA ubiquitination during DNA damage bypass, predisposes >90% of mice to tumorigenesis in multiple organs. In heterozygous *Atad5* mice, both somatic cells and the spontaneous tumors showed high levels of genomic instability. In a subset of sporadic human endometrial tumors, we identified heterozygous loss-of-function somatic mutations in the *ATAD5* gene, consistent with the role of mouse *Atad5* in suppressing tumorigenesis. Collectively, our findings suggest that *ATAD5* may be a novel tumor suppressor gene.

## Introduction

Chromosomal instability is a common feature of many tumors [1]. However, the genetic basis of chromosomal instability (CIN) in human tumorigenesis is poorly understood [2]. In contrast, genetic screens in *S. cerevisiae* have identified many genes controlling a variety of processes such as chromosome condensation, sister-chromatid cohesion, kinetochore structure and function, centrosome and microtubule formation and dynamics, and cell-cycle checkpoints [3]–[5] which, when mutated, lead to CIN. There is a growing body of evidence starting to emerge to support the idea that the presence of a CIN phenotype could lead to mammalian tumorigenesis [6]–[11]. Moreover, based on the observation that there are evolutionarily conserved synthetic lethal interactions among a number of yeast CIN genes, it has recently been proposed that identifying CIN genes that are disrupted in human cancer could lead to the design of rational therapeutics [5], [12], [13].

The yeast *ELG1* gene was originally identified in screens for suppressors of CIN and telomere maintenance proteins [3], [4], [14], [15], [16], [17]. Recently, we demonstrated that human *ATAD5* (*hELG1*), responds to a wide range of DNA damaging agents, and that reduced expression of *ATAD5* in cell lines leads to sensitivity to DNA damaging agents, and high levels of genomic instability [18]. In addition, we found that human ATAD5, together with a deubiquitinating enzyme complex, USP1-UAF1, functions to reduce the level of ubiquitinated PCNA on chromatin [19]. Defects in the regulation of PCNA ubiqutination result in deficiency in the post-replication repair pathways. Although the importance of the post-replication pathway in the suppression of tumorigenesis has been highlighted by the discovery that germline mutations in the *POLH* gene, which encodes a polymerase functioning in a post-replication repair pathway, are responsible for the variant form of Xeroderma Pigmentosum, a skin cancer-prone syndrome [20], [21], it has not yet been demonstrated whether the regulation of post-replication pathways by PCNA ubiquitination is important for tumor suppression.

Because reduced expression of *ATAD5* is associated with high levels of genomic instability *in vitro* [19], we hypothesized that reduced expression of mouse *Atad5* would also lead to genomic instability *in vivo*. Here we demonstrate that mice heterozygous for *Atad5* display high levels of genomic instability *in vivo* and that >90% of *Atad5^+/m^* mice develop a variety of spontaneous tumors. Mouse embryonic fibroblasts (MEFs) derived from the *Atad5* heterozygous mice were highly sensitive to DNA damaging agents, demonstrating high levels of aneuploidy and genomic instability in response to DNA damage. We further show that somatic, loss-of-function mutations in *ATAD5* are present in a subset of primary human endometrial cancers. Taken together, our findings provide the first evidence to indicate that mammalian *Atad5* suppresses genomic instability and tumorigenesis.

## Results

### Characterization of the Atad5 Mutant Allele in the RRF055 ES Cell Line

**Figure 1:**
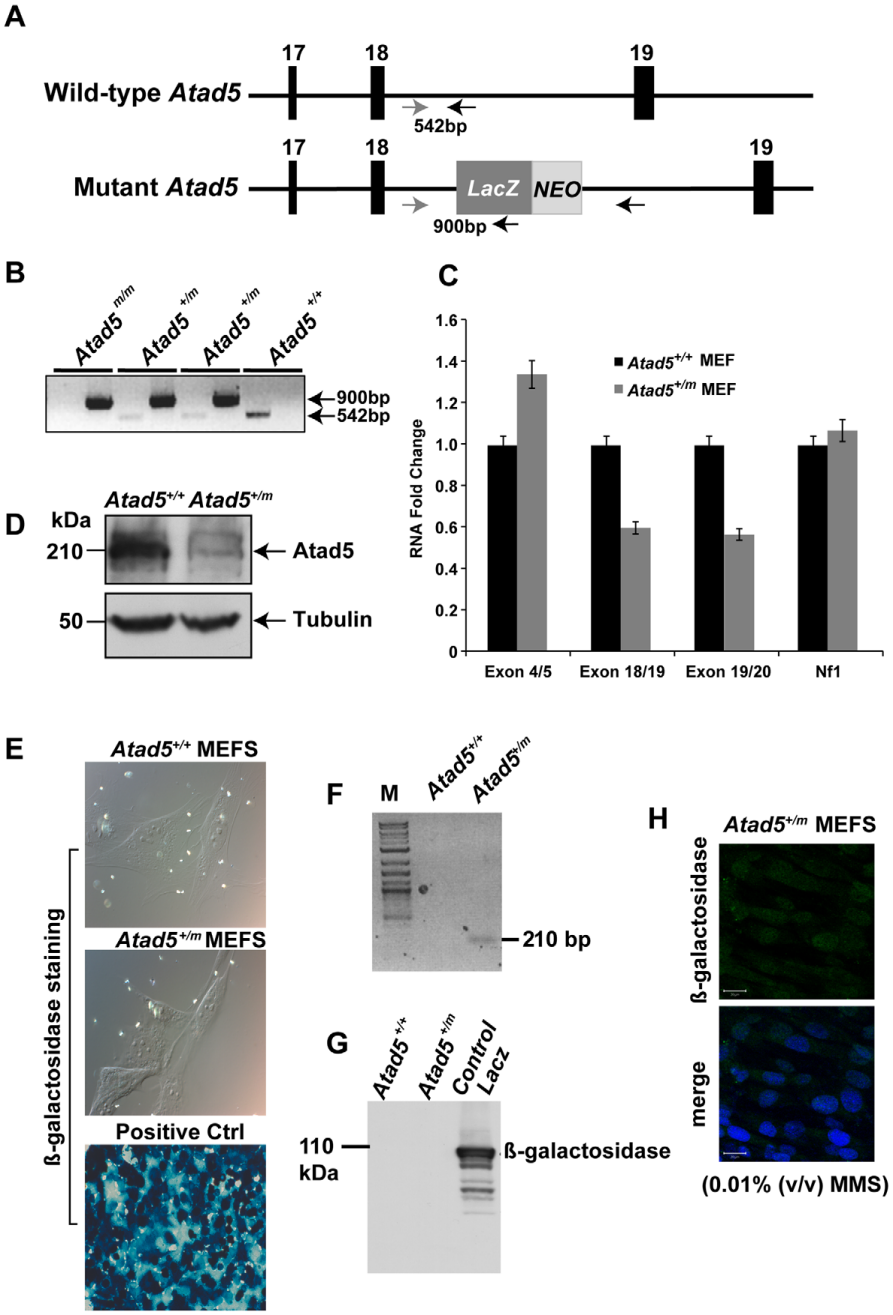
Disruption of the mouse Atad5 gene by insertional mutagenesis. (A) Schematic diagram of wild type and mutant *Atad5* loci. Exons 17–19 are indicated as solid boxes. *LacZ* and *NEO* indicate the location of the cassette that disrupted *Atad5* gene expression by insertional mutagenesis. Arrows and numbers between arrows indicate the locations of primers used for genotyping and expected sizes of PCR products, respectively. PCR with gray and black primer that can bind wild type locus generates 542 bp PCR products from wild type loci but not in mutant locus due to the inserted *LacZ-NEO* cassette. In contrast, PCR with gray and another black primer that can bind *LacZ* sequence generates 900 bp PCR product only from mutant locus. (B) PCR based genotyping of DNAs isolated from mouse embryos at day 3.5 p.c. Wild type and mutant alleles of *Atad5* are represented by + and m, respectively. 1^st^ and 2^nd^ lanes in each sample shows PCR products generated from wild type locus (542 bp) and mutant locus (900 bp) explained in the legend A. (C) RT-PCR analysis of *Atad5* mRNA levels in wild type and *Atad5^+/m^* MEFs. The exons to which each *Atad5* primers bind are indicated on the x-axis. (D) Western blot analysis of Atad5 in wild type MEFs and *Atad5^+/m^* MEFs. (E) β galactosidase expression in wild type (*Atad5^+/+^*; negative control), *Atad5^+/m^* MEF cells, and HEK 293T cells transiently transfected with a plasmid expressing β-galactosidase (positive control) were examined by immunostaining with a β-galactosidase antibody. (F) The fused *Atad5-LacZ* transcript was confirmed by RT-PCR. M stands for molecular marker. A 210 bp RT-PCR product only from *Atad5^+/m^* cDNA MEF cells was detected. (G) β galactosidase expression in wild type (*Atad5^+/+^*; negative control), *Atad5^+/m^* MEF cells, and HEK 293T cells transiently transfected with a plasmid expressing β-galactosidase (positive control) were determined by Western blot analysis. (H) Atad5- β-galactosidase fusion protein was not detected in the *Atad5^+/m^* MEF cells even after 12 hours recovery from 0.01% MMS treatment for one hour by immunostaining with a β-galactosidase antibody. Top and bottom panels are images of β-galactosidase antibody staining only and merged with DAPI, respectively.

To investigate whether Atad5 is critical for the maintenance of genomic stability *in vivo*, we generated a mouse model using a 129/Sv background embryonic stem (ES) cell line, RRF055 from Baygenomics (http://baygenomics.ucsf.edu). RRF055 has a retroviral insertion between exons 18 and 19 of the *Atad5* gene in mouse chromosome 11qB5 (79,902,914–79,949,293; Figure 1A). The retroviral insertion in the ES cells was confirmed by sequencing a PCR product amplified using a primer set specific to sequences at the intron following exon 18 of *Atad5* gene and the *LacZ* gene (Figure 1A and 1B). Although we could recover *Atad5^m/m^* embryos at embryonic day 3.5, no *Atad5^m/m^* mice were born after intercrossing *Atad5^m/+^* mice (Figure 1B). There was no significant difference in the level of mRNA containing exons 4 and 5 between *Atad5^+/m^* and wild type MEFs (Figure 1C). In contrast, the expression levels of mRNA containing exons 18, 19, and 20 were reduced by half in the *Atad5^+/m^* MEFs compared to wild type MEFs. Consistent with 50% decrease in full length *Atad5* mRNA, the level of full length Atad5 protein was also reduced by at least half in *Atad5^+/m^* MEFs compared to wild type MEFs (Figure 1D). For *Atad5*, the RefSeq transcript is the only transcript that represents a full-length cDNA. Although there are several shorter, putative transcripts included in the UCSC, Ensembl, and NCBI genome browsers these are based on large-scale cDNA sequencing projects and are not sufficiently well annotated to be included in the Consensus CDC project, which is a collaborative effort to identify a core set of protein coding regions.

Mouse *Atad5* is located close to the *Nf1* tumor suppressor gene. To exclude the possibility that the retroviral insertion affected the expression of *Nf1*, the mRNA level of *Nf1* was measured in *Atad5^+/m^* MEFs. There was no significant difference in the mRNA expression of *Nf1* in *Atad5^+/m^* MEFs compared to wild type MEFs indicating that the retroviral insertion only reduced the level of Atad5 (Figure 1C).

To determine whether the fused mRNA of *Atad5* and *LacZ* generated a hybrid protein, we analyzed β galactosidase expression in *Atad5^+/m^* MEFs either by immunohistochemistry or by western blotting of whole cell extracts with β-galactosidase or Atad5 antibodies. No protein expression was observed (Figure 1E and 1G) although RT-PCR analysis with a primer for *Atad5* exon 18 and another primer for *LacZ* demonstrated expression of a fused mRNA that was in-frame as determined by sequencing the RT-PCR product (Figure 1F). The human ATAD5 protein is stabilized and forms foci in the nuclei of cultured cells in response to DNA damage [18]. Therefore, we reasoned that if the β-galactosidase-fused Atad5 were expressed, it would form foci and be more readily detectable in the presence of DNA damage. After one hour of 0.01% methyl methane sulfonate (MMS) treatment, we did not detect any foci formation of β-galactosidase-fused Atad5 (Figure 1H). We conclude that an mRNA encoding the N-terminal 1347 amino acids of Atad5 fused with *LacZ* is transcribed, but the fused protein is not stable.

### Atad5 Deficiency Causes Genomic Instability

**Figure 2:**
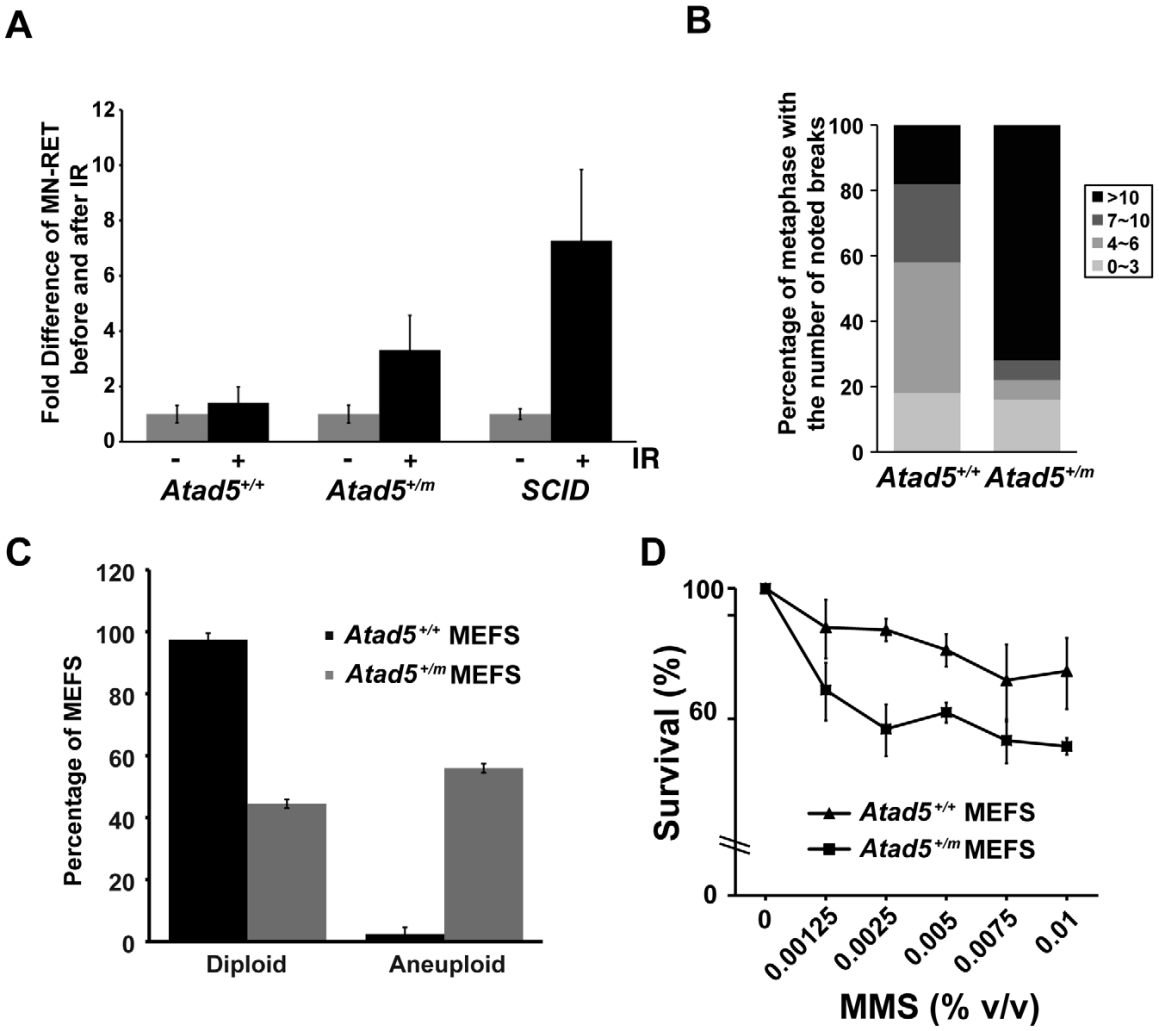
The heterozygous *Atad5^+/m^* mutation causes genomic instability *in vivo* and in MEFs. (A) *Atad5^+/m^* mice exhibited an increase in IR-induced MN-RETs. Wild type (*Atad5^+/+^*) and SCID mice were treated with IR and their peripheral blood cells were analyzed in the same manner for comparison. CD71 and propidium iodide (PI) were used to detect RET cells and micronuclei, respectively. Fold induction after IR irradiation in each mouse with a different genotype normalized to MN-RET from unirradiated mice is presented in the graph. Five wild type, five *Atad5^+/m^*, and two SCID mice were analyzed. (B) MEFs derived from the *Atad5^+/m^* mice produced more chromatid breaks than wild type MEFs in response to MMS treatment. Percentages of metaphases having noted number of breaks are presented as graphs. Noticeable chromatid breaks per cell were quantified for 50 metaphases from both wild type and the *Atad5^+/m^* MEFs. (C) MEFs derived from the *Atad5^+/m^* mice have a high level of aneuploidy. (D) Survival of wild type and the *Atad5^+/m^* MEFs following MMS treatment is displayed as the percentage of viable cells relative to untreated cells. P-values were calculated or each data point as follows, 0.00125% MMS (p = 0.05); 0.0025% MMS (p = 0.002), 0.005% MMS (p = 0.005), 0.0075% MMS (p = 0.09); and 0.01% MMS (p = 0.03), respectively.

To investigate whether Atad5 suppresses genomic instability *in vivo*, we used two different assays. In the first assay, we compared the frequency of micronuclei formation in peripheral blood reticulocytes following ionizing radiation treatment of wild type and *Atad5^+/m^* mice. Unlike anucleated RETs that are produced during normal differentiation, micronucleated reticulocytes (MN-RETs) have a micronucleus that is produced by lagging or fragmented chromosomes resulting from genomic instability [22]. Therefore, the frequency of MN-RETs provides a measure of genomic instability. In response to 0.7 Gy γ-irradiation, the frequency of MN-RETs in *Atad5^+/m^* mice increased 3.3-fold after 48 hours compared to the 1.4-fold increase observed in wild type mice (Figure 2A). The increase in MN-RETs observed in *Atad5^+/m^* mice was about half of the 7.3-fold increase observed in severe combined immune deficient (SCID) mice, which are defective in DNA-dependent protein kinase catalytic subunit, a major DNA double-strand break repair enzyme. We conclude that the heterozygous mutation of *Atad5* causes genomic instability *in vivo* following exposure to γ-irradiation.

In a second assay, we measured genomic instability in cultured cells. *Atad5^+/m^* mouse embryo fibroblasts (MEFs) exhibited a high level of genomic instability in response to MMS (Figure 2B). Following 0.01% MMS treatment, 72% of *Atad5^+/m^* MEFs had more than 10 chromatid breaks compared to 18% of wild type MEFs. Furthermore, untreated *Atad5^+/m^* MEFs displayed a significantly higher level of spontaneous aneuploidy compared to *Atad5^+/+^* mice (Figure 2C). In addition, *Atad5^+/m^* MEFs increased sensitivity to MMS treatment compared to wild type MEFs (Figure 2D).

To investigate whether the genomic instability observed in *Atad5^+/m^* mice is evolutionarily conserved, we constructed *ATAD5*-deficient heterozygous DT40 cells by targeted knockout of one allele of *chATAD5*, and monitored their sensitivity to DNA damage. In two individual heterozygous *ATAD5*-deficient chicken DT40 clones, DNA damaging agent sensitivity was observed (Figure S1). In addition, *ATAD5*-deficient heterozygous DT40 cells exhibited higher levels of chromosomal instabilities in response to ionizing radiation than wild type DT40 cells (Figure S1). Collectively, these findings indicate that heterozygous mutation of the *Atad5* gene in mouse and chicken cells causes a defect in the DNA damage response, which is consistent with the known role of yeast Elg1 and human ATAD5 in suppressing genomic instability [18], [23], [24], [25].

**Figure 3:**
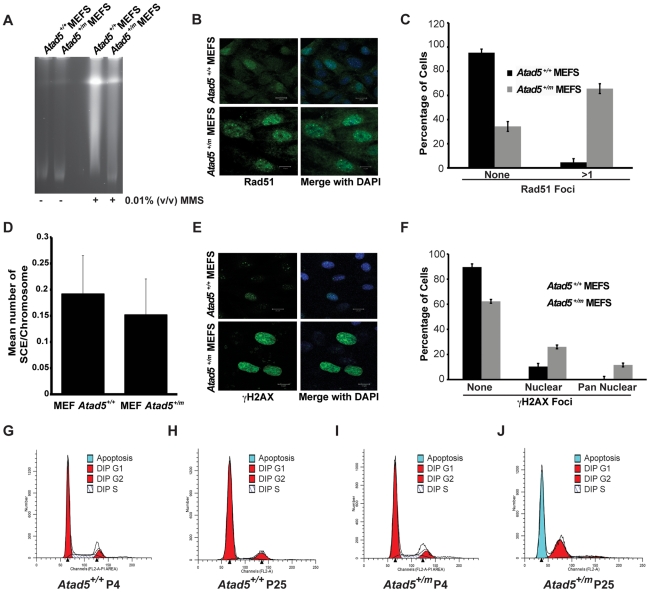
MEFs derived from the *Atad5^+/m^* mice are defective in DNA repair, display a hyper-recombination phenotype, and are apoptosis-prone. (A) *Atad5^+/m^* MEFs are defective in DNA repair. Broken DNAs after 0.01% MMS treatment were visualized by pulse field gel electrophoresis. (B, C) *Atad5^+/m^* MEFs display high levels of spontaneous Rad51 foci in the nucleus. B and C show, respectively, an actual image and a graphic presentation of the number of Rad51 foci in the nucleus of each cell type. (D) *Atad5^+/m^* MEFs display similar rates of sister chromatid exchange rate compared to wild type MEFs. (E, F) *Atad5^+/m^* MEFs display high level of γH2AX foci in the nucleus. An actual image (E) and a graphic presentation (F) of the number of cells having different types of γH2AX foci in the nucleus from each cell type are shown. (G, H, I, J) *Atad5^+/m^* MEFs undergo apoptosis at the 25^th^ passage. FACS analysis of wild type (G, H) and *Atad5^+/m^* MEFs (I, J) from the 4^th^ passage (G, I) and the 25^th^ passage (H, J) were performed based on their DNA contents. Bars in images for *Atad5^+/+^* and *Atad5^+/m^* (B and E) indicate 20 µm and 10 µm, respectively.

The high levels of genomic instability and sensitivity to DNA damaging agents that we observed in *Atad5^+/m^* MEFs (Figure 2) suggested that Atad5 haploinsufficiency causes defects in DNA repair. To test this hypothesis, DNA from wild type and *Atad5^+/m^* MEFs were analyzed by pulsed-field gel electrophoresis after 0.01% MMS treatment and a 16 hour recovery period. *Atad5^+/m^* MEFs displayed smaller DNA fragments following 0.01% MMS treatment compared to wild type MEFs (Figure 3A), suggesting a defect in DNA repair in *Atad5^+/m^* MEFs.

DNA repair deficiency generates spontaneous DNA damage. Consistent with this paradigm, after 25 passages in culture without treatment by DNA damaging agents, *Atad5^+/m^* MEFs had a large population of cells with spontaneous Rad51 foci, reflecting the accumulation of DNA damage and possibly hyper-recombination phenotype in cells (Figure 3B and 3C). Although there was an increase in Rad51 foci formation in *Atad5^+/m^* MEFs, we did not observe any significant increase in the sister chromatid exchange rate in *Atad5^+/m^* MEFs compared to wild type MEFs (Figure 3D). To independently investigate the accumulation of spontaneous DNA damage in *Atad5^+/m^* MEFs, H2AX phosphorylation (γ-H2AX) was monitored in wild type and *Atad5^+/m^* MEFs after 25 passages in culture. The frequency of cells exhibiting γ-H2AX foci was greater in *Atad5^+/m^* MEFs than wild type MEFs (Figure 3E and 3F).

Approximately 10% of *Atad5^+/m^* MEFs showed pan nuclear γ-H2AX staining, which is frequently observed in cells undergoing apoptosis (Figure 3E and 3F). To determine whether *Atad5^+/m^* MEFs undergo apoptosis, the DNA content of wild type and *Atad5^+/m^* MEFs was analyzed by FACS at passage 4 and passage 25. Although there was no clear difference in wild type and *Atad5^+/m^* MEFs at passage 4, *Atad5^+/m^* MEFs but not wild type MEFs underwent apoptosis at passage 25 (Figure 3G–3J). To understand the molecular defects that cause DNA repair deficiency in *Atad5^+/m^* MEFs, PCNA ubiquitination was investigated. PCNA ubiquitination was increased more in response to MMS in *Atad5^+/m^* MEFs compared to wild type MEFs (Figure S2). However, it is important to point out that we did not observe a similar increase of PCNA ubiquitination in *ATAD5* heterozygous chicken DT40 cells. Taken together, these data demonstrate that Atad5 haploinsufficiency causes defects in DNA repair, which results in the accumulation of spontaneous DNA damage that triggers aneuploidy and apoptosis and is associated with an inability to reduce PCNA ubiquitination in the nucleus.

### *Atad5^+/m^* Mice Develop a Wide Spectrum of Spontaneous Tumors That Have High Levels of Genomic Instability

**Figure 4:**
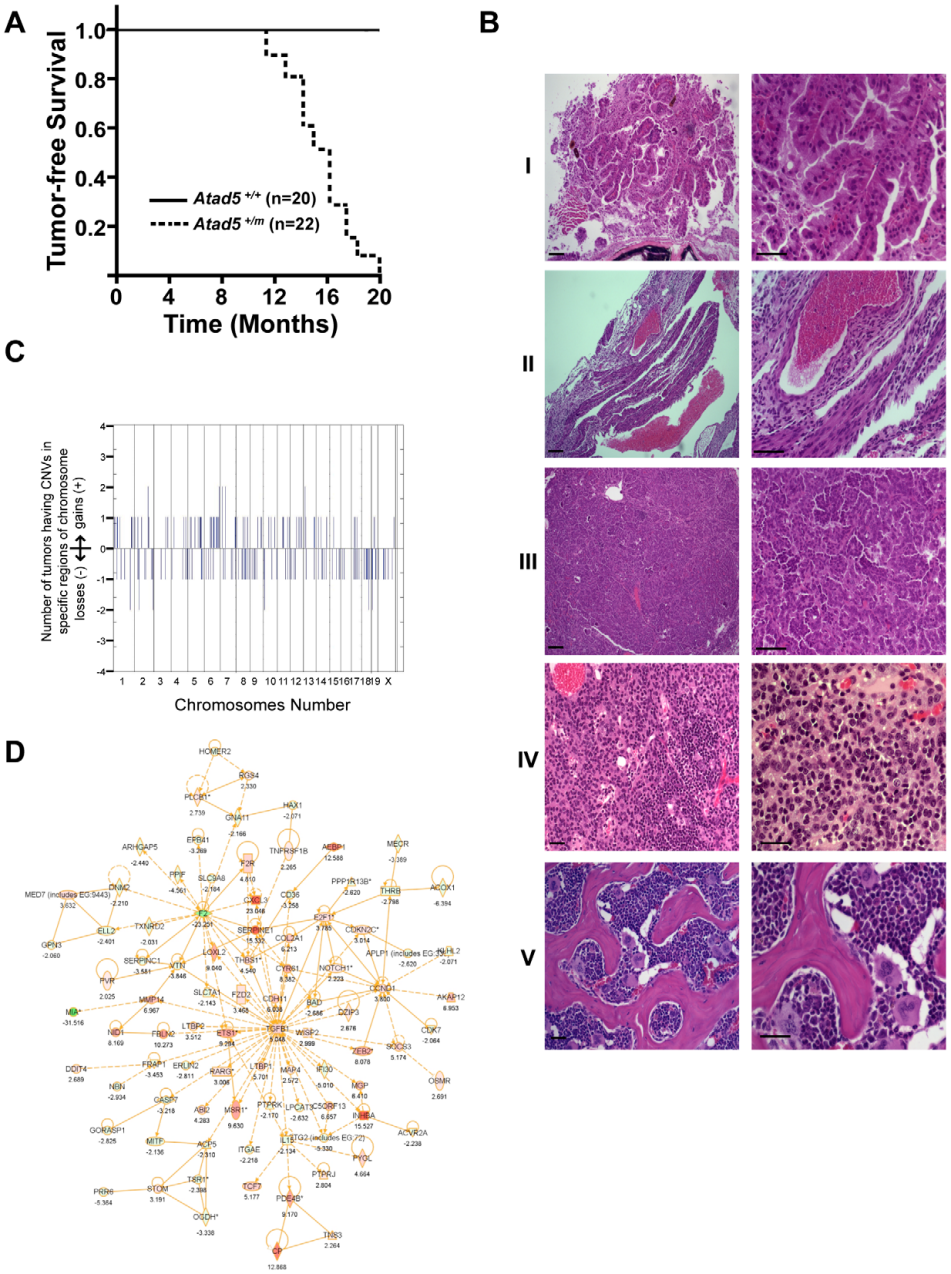
*Atad5^+/m^* mice are tumor-prone. (A) Kaplan-Meier survival graph of wild type (*Atad5^+/+^*; n = 20) and the *Atad5^+/m^* mice (n = 22). (B) Representative H&E stained sections of tumors that arose in the *Atad5^+/m^* mice; (I) Harderian gland adenoma, (II) hemangioma, (III) lung bronchiolar adenoma, (IV) thymoma, and (V) sarcoma from bone marrow. The panel on the right is a two-fold magnification of panel on the left. (C) Frequency of genomic copy number gains and losses among four tumors from the *Atad5^+/m^* mice, plotted as a function of genome location with all chromosomes starting from 1pter (left) to X (right). Vertical lines indicate chromosome boundaries. Positive and negative values indicate the number of tumors that showed copy number gains (+) and losses (−), respectively. (D) The pathway activated in two tumors from the *Atad5^+/m^* mice analyzed by microarray. Red and green colors represent the activation and inactivation in tumors, respectively.

To investigate whether the genomic instability found in the *Atad5^+/m^* mice leads to tumorigenesis, we monitored a cohort of 42 mice (22 *Atad5^+/m^* mice and 20 wild type age matched controls) with a normal diet over time. All 22 *Atad5^+/m^* mice either died or had to be sacrificed due to ill-health between 11 and 20 months of age whereas all of the wild type mice survived beyond 20 months (p value<0.001) (Figure 4A). Twenty of 22 (90.9%) *Atad5^+/m^* mice developed tumors compared to 4 of 20 (20%) wild type mice (p value<0.001) (Figure 4A and 4B). Tumors that developed in wild type mice were lung adenomas, which appeared after 24 months. In contrast, necropsy analyses revealed that *Atad5^+/m^* mice developed a variety of tumors in many tissues and cell types with a high incidence of adenocarcinomas and sarcomas (Figure 4B and Table 1). The predominance of adenocarcinomas and sarcomas observed in *Atad5^+/m^* mice is in stark contrast to the range of tumors observed in the 129/Sv strain used in this study. Wild type 129/Sv mice develop tumors spontaneously in 7% of male mice with no prevalence of a specific type [26]; there have been no reports of wild type 129/Sv strain mice developing adenocarcinomas and sarcomas at a high frequency.

**Table 1:** Various spectra of tumors were observed in heterozygous *Atad5* mice.

Age ¶(months)	Sex	Tumor Type	Organ
11	F	Sarcoma	Skeletal muscles
14	F	Sarcoma	Uterus
11	F	Granulosa Cell Tumor	Ovary
14	F	Granulosa Cell Tumor	Ovary
13	F	Spindle Cell Tumor	Small intestine/Stomach
17	F	Adenocarcinoma	Lung
18	F	Adenocarcinoma	Harderian glands
20	F	Adenocarcinoma	Harderian glands
14	M	Carcinoma	Lung
14	M	Adenocarcinoma	Harderian glands
15	M	Sarcoma	Peripheral nerve
15	M	Lymphoma	Lymph/Lung/Liver
15	M	Adenocarcinoma	Harderian glands
16	M	Sarcoma	Tail
16	M	Sarcoma	Hindlimb
16	M	Adenocarcinoma	Harderian glands
16	M	Adenocarcinoma	Harderian glands/Lung
17	M	Thymoma	Thymus
17	M	Hemangiosarcoma	Subcutis
18	M	Adenocarcinoma	Liver

Two *Atad5^+/m^* mice that did not develop tumors had abscesses and died from apparent unresolved infections.

**¶:** Age at death or sacrifice due to ill-health.

Because ATAD5 has been suggested to suppress genomic instability in yeast and human cells [4], [18], [24], we hypothesized that the tumors that arose in the *Atad5^+/m^* mice might be associated with high levels of genomic instability. We used array-comparative genome hybridization to determine the genomic copy number profiles of 2 adenomas and 2 sarcomas from the *Atad5^+/m^* mice. This revealed numerous copy number variations (CNVs) affecting almost all chromosomes (Figure 4C and Table S1), indicating that the tumors that arose in *Atad5^+/m^* mice had enhanced genomic instability. Furthermore, several genomic regions were gained or lost in multiple tumors.

We next compared the gene expression profiles of one adenocarcinoma and one sarcoma from *Atad5^+/m^* mice with those of their surrounding tissues, by microarray analysis. In total, there was a 2-fold or greater change in the expression of 584 genes, including 198 cancer-related genes (103 up-regulated and 95 down-regulated), in both tumors compared to the surrounding tissues. We selected a number of genes that were dysregulated in tumors and validated the different levels of expression by qRT PCR (Figure S3B and S3C). When the genome-wide CNVs and gene expression profiles of tumors from the *Atad5^+/m^* mice were compared, there was a high degree of positive correlation between them; 143 of 584 genes that were up- or down-regulated in tumors appeared to be due to CNVs (Table S3).

To determine whether a specific pathway(s) was activated or suppressed in tumors, the microarray datasets were analyzed with the Ingenuity Pathway Analysis software (Figure 4D, Figure S3, and Table S2). The expression of genes in the TGF-β signaling network was significantly up-regulated in both tumors (Figure 4D). We concluded that at least a subset of tumors from the *Atad5^+/m^* mice was associated with the activation of TGF-β signaling. Whether this finding reflects the progression of tumorigenesis or the mechanism of tumor initiation associated with *Atad5* haploinsufficiency has not been determined.

### Human ATAD5 Is Somatically Mutated in Primary Endometrial Cancers

The high incidence of cancer in the *Atad5^+/m^* mice, led us to hypothesize that somatic mutations of *ATAD5* might be associated with human cancers. To test this hypothesis we searched for somatic *ATAD5* mutations among a series of sporadic human endometrial tumors. We focused on endometrial cancer because aneuploidy, a form of CIN, is frequently observed in non-endometrioid endometrial cancers (NEECs) and occurs at a lower frequency in endometrioid endometrial cancers (EECs) [27]. We resequenced all coding exons of *ATAD5* from 108 primary endometrial tumors, consisting of 66 NEECs and 42 EECs. Somatic mutations were distinguished from germline polymorphisms by resequencing the variant position from matched normal DNAs.

**Figure 5:**
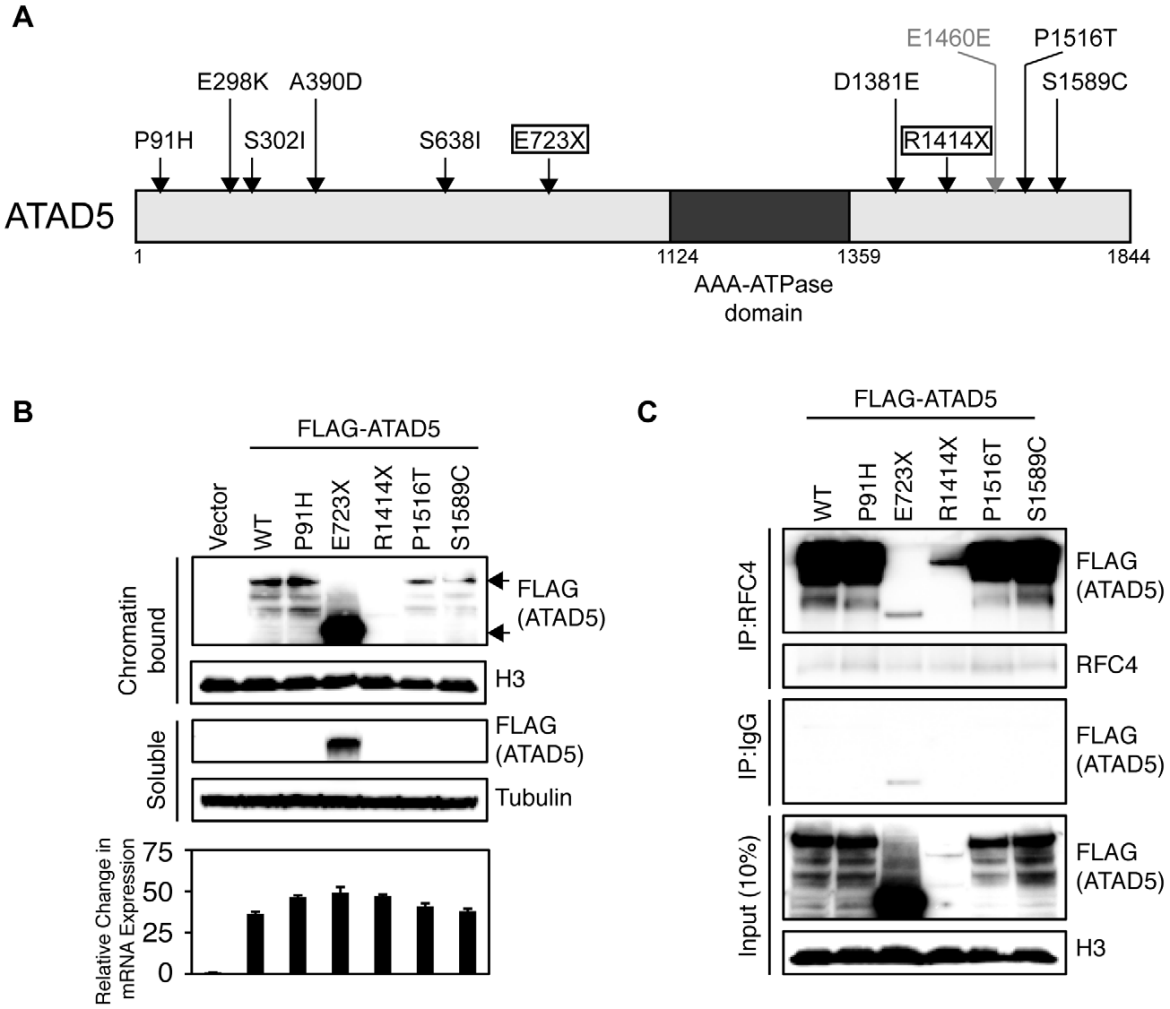
Somatic mutations of *ATAD5* in primary endometrial tumors exhibit functional defects *in vitro*. (A) Schematic representation of the ATAD5 protein showing positions of somatic mutations (arrows) relative to the AAA-ATPase domain. Truncating (boxed), missense (black) and synonymous (gray) mutations are distinguished. Amino acids are numbered. (B) The ATAD5-R1414X mutant is associated with reduced protein levels. HEK 293T cells were transfected with constructs expressing wild type (WT) *ATAD5* or mutant *ATAD5*. Soluble fractions, which include cytoplasmic and nucleoplasmic proteins, and chromatin-bound fractions were separated by SDS-PAGE after 48 h. Relative expression of wild type or mutant ATAD5 was determined by Western blotting with anti-FLAG-antibody. Histone H3 and tubulin were used as loading controls for chromatin-bound and soluble fractions respectively. Total RNAs, extracted from cells used in the Western blot analysis, served as templates for RT-PCR. *ATAD5* mRNA expression levels determined by quantitative RT-PCR, were normalized to β-actin expression levels and are represented as a relative value compared to that of vector transfectants. (C) The ATAD5-E723X mutant protein does not interact with RFC4. Chromatin-bound fractions from HEK 293T cells transfected with constructs expressing wild type or mutant *ATAD5* were immunoprecipitated (IP) with anti-RFC4 antibody or IgG control antibody, and subjected to Western blot analysis.

Overall, we identified 11 somatic mutations in the *ATAD5* gene in 4.6% (5 of 108) of endometrial tumors (Table 2). The frequency of somatic *ATAD5* mutations was higher among NEECs (6.0%, 4 of 66) than EECs (2.3%, 1 of 42), although this difference did not reach statistical significance (*P* = 0.48, Fisher's exact test of significance). The observed 10∶1 ratio of nonsynonymous∶synonymous (NS∶S) *ATAD5* mutations was 5-fold higher than an expected 2∶1 ratio in the absence of selection. All of the somatic *ATAD5* mutations occurred exclusively at cytosine or guanine residues, and two tumors had multiple mutations. Taken together, these observations raise the possibility that some of these tumors may have an underlying a hypermutable phenotype. Two *ATAD5* mutations were nonsense mutations predicted to cause premature protein truncation (Figure 5A). One of the nonsense mutations (ATAD5-R1414X) occurred in an almost equivalent position to the insertion mutation within the *Atad5* tumor-prone mouse described here.

**Table 2:** Somatic mutations of *ATAD5* were identified in primary human endometrial tumors.

Case no.†	Tumor subtype	Nucleotide change	Amino acid change	Mutation type	Predicted effect of missense mutations on protein function	
					By SIFT	By PolyPhen
T3	NEEC	c.G905T	S302I	Missense	Tolerated	Probably damaging
T3	NEEC	c.C1169A	A390D	Missense	Tolerated	Probably damaging
T3	NEEC	c.G2167T	E723X	Nonsense	-	-
T3	NEEC	c.G4380A	E1460E	Synonymous	-	-
T113	NEEC	c.G892A	E298K	Missense	Tolerated	Benign
T113	NEEC	c.G1913T	S638I	Missense	Tolerated	Possibly damaging
T113	NEEC	c.C4546A	P1516T	Missense	Tolerated	Probably damaging
T113	NEEC	c.C4143A	D1381E	Missense	Tolerated	Probably damaging
T51	NEEC	c.C4240T	R1414X	Nonsense	-	-
T62	NEEC	c.C4766G	S1589C	Missense	Tolerated	Probably damaging
T88	EEC	c.C272A	P91H	Missense	Tolerated	Benign

†Case no T3 is also known as OM-1323.

### Somatic Mutations of Human ATAD5 Result in Haploinsufficiency and Cause Defects in ATAD5 Function

To test whether the somatic *ATAD5* mutations we observed in human endometrial tumors encode loss-of function proteins, we compared the functional properties of wild type and mutant (P91H, P1516T, S1589C, E723X, and R1414X) forms of ATAD5. These five mutants were chosen for analysis because they occurred in a mutually exclusive fashion in endometrial tumors. We analyzed the cellular distribution and stability of the ATAD5 proteins by transiently transfecting human embryonic kidney (HEK) 293T cells with Flag-tagged wild type or mutant *ATAD5* constructs. Wild type ATAD5 protein as well as the P91H, P1516T, S1589C, and R1414X mutant proteins were found exclusively in the chromatin-bound fraction (Figure 5B). Within the chromatin-bound fraction, the levels of the ATAD5-P91H mutant protein were comparable to that of wild type ATAD5 whereas the protein levels of the ATAD5-P1516T and ATAD5-S1589C mutants were slightly lower than the wild type protein and the level of the ATAD5-E723X protein was greater than that of the wild type protein (Figure 5B). The ATAD5-E723X truncated mutant protein was detected in both the soluble fraction and the chromatin-bound fraction of transfected cells (Figure 5B) suggesting that the ATAD5-E723X truncation mutant has impaired chromatin retention. The ATAD5-R1414X truncated protein was undetectable either in the chromatin-bound or the soluble fractions of transfected cells (Figure 5B) despite the observation that the *ATAD5-R1414X* transcript was expressed at comparable levels to that of the wild type transcript. This is consistent with our finding that the mouse model used in this study makes a transcript encoding the N-terminal 1347 amino acids of *Atad5* fused with *LacZ* but fails to produce a stable protein (Figure 1). Since haploinsufficiency for *Atad5* leads to cancer predisposition in our mouse model, we sought to determine whether the wild type *ATAD5* allele is expressed in the human endometrial tumor (T51) with the *ATAD5-R1414X* mutation. By sequencing RT-PCR products generated from purified populations of tumor cells obtained by laser capture microdissection, we observed expression of both the wild type and *ATAD5-R1414X* alleles (Figure S4). We conclude that the truncated ATAD5-R1414X mutant protein is unstable and that it may contribute to endometrial cancer by a haploinsufficient mechanism.

ATAD5 forms an RFC-like complex by binding to RFC4, an invariant subunit of both RFC and RFC-like complexes in mammalian cells [17]. Immunoprecipitation of RFC4 from transiently transfected HEK 293T cells, followed by immunoblotting with an anti-Flag antibody, demonstrated that the ATAD5-E723X truncation mutant had a greatly reduced ability to bind to RFC4 (Figure 5C). This observation indicates that the ATAD5-E723X mutant has an impaired ability to form an intact ATAD5-RFC complex. This is consistent with previous reports that the C-terminal region of yeast Elg1p is essential for complex formation with other RFC subunits [28]. We conclude that the presence of the ATAD5-E723X truncated protein in both the soluble and chromatin bound fractions of the cell (Figure 5B), may be the result of a defect in the ability to form an intact ATAD5-RFC complex. Despite the low levels of the ATAD5-R1414X truncated protein present in the chromatin-bound fraction, this mutant retained the ability to bind to RFC4 (Figure 5C). The reduced levels of the ATAD5-R1414X protein and the reduced binding of the ATAD5-E723X protein to RFC4 are consistent with a loss of ATAD5 function. Although the three missense mutants interacted with RFC4, it is possible that they might affect the function of the assembled protein complex by other mechanisms not tested here; alternatively, these mutations might be so-called “passenger” mutations that have no effect on ATAD5 function.

## Discussion

There is an emerging consensus that mutations in chromosomal instability genes contribute to many kinds of malignancy [29]. For example, previous studies of endometrial carcinomas have uncovered somatic alterations of a number of genes implicated in the maintenance of genomic integrity including somatic mutations in *CDC4/FBXW7*, and *p53*, and amplification or altered expression of *STK15*, *BUB1*, *CCNB2*, and *CCNE* [30]–[33]. In this report we have demonstrated the first somatic *ATAD5* mutations in human cancer through a resequencing analysis of primary endometrial carcinomas. The frequency of somatic *ATAD5* mutations was somewhat higher among NEECs than EECs. NEECs are the most clinically aggressive subtype of endometrial cancer that, as a group, exhibit aneuploidy, a form of chromosomal instability, much more frequently than EECs do [27]. Although *ATAD5* was mutated at low frequency in endometrial carcinomas, this is not unexpected in light of recent studies of whole exomes of breast, colorectal and pancreatic cancers [34]–[36]. Those studies revealed that most somatically mutated genes are mutated at low frequency but that many infrequently mutated genes impinge on a smaller number of discrete biological processes and biochemical pathways resulting in a significant overall contribution to tumorigenesis. In this regard, it will be interesting, in future studies, to determine the extent to which the ATAD5 pathway is disrupted in sporadic endometrial cancer.

The microarray analysis and quantitative PCR of mouse tumor samples showed that the wild type allele of *Atad5* was retained and was expressed normally. In addition, human endometrial tumors carrying *ATAD5*-R1414X mutation expressed both the wild type and mutant alleles. These data are consistent with a mechanism by which haploinsufficiency of *ATAD5* causes genomic instability leading to malignant transformation.

We have also recently reported increased CIN in a human cell line expressing reduced levels of ATAD5 following siRNA silencing [18]. The reduced expression of *ATAD5* in human cells resulted in a spontaneous hyper-recombination phenotype [19]. Finally, primary *Atad5^+/m^* fibroblasts had high levels of aneuploidy and other visible genome damage in culture, even in the absence of DNA damaging agents and tumors from *Atad5^+/m^* mice exhibited a large number of spontaneous genomic copy number alterations. It is possible that the high level of spontaneous genomic damage might be due to defects in the removal of ubiquitin from PCNA by ATAD5 together with USP1-UAF1 after DNA damage bypass [19]. In this model the persistent presence of ubiquitinated PCNA on DNA could retain translesion synthesis DNA polymerases in DNA replication forks. Due to their low processivity, DNA replication forks that have prolonged translesion synthesis DNA polymerases could collapse and abnormal recombination might enact to create a high level of genomic instability and tumors in the *Atad5^+/m^* mouse. Consistent with this possibility, we observed higher levels of PCNA ubiquitination in response to MMS in *Atad5^+/m^* MEFs compared to wild type MEFs. Interestingly, the *Atad5^+/m^* MEFs displayed high levels of spontaneous Rad51 foci that might lead to a high level of recombination although there was no significant induction of sister chromatid exchange rate. However, it is worth noting that recent studies have shown that the *Usp1* knock-out mice, and the *PCNA-K164R* knock-in mice both have milder phenotypes [37], [38] compared to *Atad5^+/m^* mice. Therefore we cannot exclude the possibility that tumorigenesis in the *Atad5^+/m^* mice might not be due to the dysregulation of PCNA ubiquitination, but instead might result from other defects caused by Atad5 haploinsufficiency. For example, it is possible that haploinsufficiency for *Atad5* might affect DNA replication especially in Okazaki fragment maturation during lagging strand synthesis because it has been observed that yeast Elg1 interacts with Rad27, the yeast homolog of the FEN1 flap endonuclease, which specifically removes RNA primers, during lagging strand maturation [17].

Late passage *Atad5^+/m^* MEFs undergo apoptosis in the absence of DNA damaging agents. This is somewhat paradoxical because tumorigenesis is frequently associated with impaired apoptosis. Nonetheless, there are several examples of tumorigenesis requiring apoptosis [39], [40]. We speculate that tumors that arise in *Atad5^+/m^* mice might be result from high levels of cell turnover associated with the increased attrition of stem cells that presumably accompanies high levels of spontaneous DNA damage. Therefore, there was increased risk of transformation. Alternatively, it is possible that the DNA damage that occurs in *Atad5^+/m^* cells might lead to the accumulation of additional mutations that render *Atad5^+/m^* cells resistant to apoptosis, thus accounting for the 11-month latency period before the appearance of tumors.

In conclusion, our study provides the first evidence that loss of Atad5/ATAD5 function is associated with tumorigenesis. Our findings warrant future investigations to explore the role of *ATAD5* in other human cancers. As concerted efforts to systematically sequence entire tumor exomes and genomes move forward rapidly, we predict that a wider spectrum of sporadic human tumors will harbor acquired somatic *ATAD5* mutations. Furthermore, given our observation that haploinsufficiency for *Atad5* in mice predisposes to tumorigenesis, it will also be important to determine whether germline variants of *ATAD5* confer increased risk for cancer. Notably, there are studies suggesting a higher cancer incidence in approximately 5 to 10% of Neurofibromatosis type 1 patients who have a micro-deletion that includes *NF1*, *ATAD5*, and several other genes [41], [42]. The high incidence of tumors in the heterozygous *Atad5^+/m^* mice in this study raises the possibility that the tumors in *NF1* microdeletion patients might arise as a result of a synergistic effect mediated by the co-deletion of *ATAD5*.

## Materials and Methods

### Generation of *Atad5^+/m^* Mice

Mice used in this study were derived from the ES cell line (ID: RRF055, 129/Sv background). Chimeric male mouse generated from the ES cell injected C57BL/6 blastocytes were transferred into pseudopregrant CD1 female recipients. All mice works were conducted under in compliance with federal regulations and guidelines. All mice work in this study was approved by the NHGRI Animal Care and Use Committee (NIH animal study proposal: G-05-2, KM as a PI).

### MN-RET Assay

We followed the method described previously [22]. Briefly, six-week old male mice were exposed to 0.7 Gy of γ-ray irradiation from a ^137^Cs source. Normochromatic erythrocytes (NCEs) and reticulocytes (RETs) in erythrocytes were distinguished by their negative and positive expressions of CD71 in cell surface, respectively. The RETs carrying micronuclei were detected by the nucleic acid binding agent propidium iodide. At least 10,000 RETs and 500,000 NCEs were analyzed in each blood sample.

### Clinical Specimens

Anonymized primary endometrial tumor tissues (n = 102) and matched histologically normal tissues were obtained from the Cooperative Human Tissue Network, or from the Biosample Repository at Fox Chase Cancer Center, Philadelphia PA. Six cases of matched tumor and normal DNAs were purchased from Oncomatrix. All primary tumor tissues were pretreatment specimens, snap-frozen within 30 minutes of surgical removal. A hematoxylin and eosin (H&E) stained section was cut from each tumor specimen and reviewed by a pathologist to verify histology and to delineate regions of tissue with a tumor cell content of ≥70%. All specimens and accompanying clinicopathological information were anonymized, and procured with appropriate IRB approval. The Office of Human Subjects Research declared exempt for human specimen usage because the specimens were anonymized when investigators received them: The exemption numbers are #3529, #3456, and #3534.

### Identity Testing

To confirm that tumor-normal pairs were consistent with derivation from the same individual, all human DNA samples were genotyped using the Coriell Identity Mapping kit (Coriell). Genotyping fragments were size separated on an ABI-3730*xl* DNA analyzer (Applied Biosystems). Alleles were scored using GeneMapper software.

### Primer Design, PCR Amplification, and Nucleotide Sequencing

Primer pairs to amplify *ATAD5* were designed using published methods [43] (Table S4). PCR products were subjected to bidirectional Sanger sequencing using the BigDye Terminator Version 3.1 Cycle Sequencing Kit (Applied Biosystems). Sequencing reactions were run on ABI 3730*xl* DNA Analyzers (Applied Biosystems). Sequence data was analyzed as described in the Supplemental Experimental Procedures.

### Laser Capture Microdissection and RT-PCR

Pure populations of tumor cells were isolated from heterogeneous tissue sections by laser capture microdissection (LCM) using an Arcturus PixCell IIe system. RT-PCR products, generated from microdissected tumor cells were sequenced to discriminate between monoallelic and biallelic expression of mutant *ATAD5* alleles.

### Analysis of *Atad5^+/m^* ES Cells, MEFs, and Mice

The wild-type and mutant *Atad5* alleles were distinguished by PCR using primers: 1298NF (5′- GAG ACT GTC TCA CCA TGT ACA GGG-3′), 1372NR (5′- ATA AAT TGT AAA AAA TCG ATC TCT-3′) and pGKR (5′- GTG GCC TGT CCC TCT CAC CTT CTA-3′). PCR products of 542 bp and 900 bp distinguished the wild-type and mutant alleles, respectively (Figure 1B). PCR conditions were an initial denaturation step at 94°C for 5 minutes, followed by 35 cycles of a denaturation step at 94°C for 30 seconds, an annealing step at 55°C for 30 seconds, and an elongation step at 72°C for 60 seconds and by a final elongation step at 72°C for 7 minutes. There were no additional insertions of the retroviral vector in the genome of RRF055 ES cells, as confirmed by Southern hybridization with a NEO probe. The ES cells were injected to into C57BL/6 blastocytes that were transferred into pseudopregnant CD1 female recipients. A resulting chimeric male mouse was mated with C57BL/6 females. Germline transmission was confirmed in progeny of agouti mice. The germline transmitted heterozygous mice were crossed with wild-type 129/Sv mice to increase the heterozygous population. In addition, two heterozygous mice were mated to generate *Atad5^m/m^* mice. Mice were bred and maintained under a protocol (#G-05-02) approved by the Institutional Animal Care and Use committee in the NHGRI, US NIH Animal Care Facility. Young *Atad5^+/m^* mice did not show any distinct phenotypic features and were fertile. However, when the *Atad5^+/m^* mice were intercrossed, we did not observe any postnatal progeny that were homozygous for the *Atad5* allele (*Atad5^m/m^*).

Mouse embryonic fibroblast (MEF) cells were isolated from both wild-type and *Atad5^+/m^* embryos at day 13.5 p.c. and cultured in Dulbecco's modified Eagle's medium (DMEM) (high glucose, w/o L-glutamine, w/o sodium pyruvate, GibcoBRL) supplemented with 2 mM glutamine, 1% penicillin/streptomycin, 15% fetal calf serum, 2 mM non essential amino acids, 0.1 mM 2-β mercaptoethanol in 4% oxygen concentration. RNAs isolated from wild-type and *Atad5^+/m^* MEFs were analyzed by RT-PCR with primer sets specifically amplifying the region of mRNA containing exons 4 and 5, exons 18 and 19, or exons 19 and 20 respectively from 500 ng of total RNA isolated with TRIzol reagent (Invitrogen). The expression of β-actin was used as an internal expression control. For quantitative RT-PCR, reactions were performed using the ABI7300 sequence Detection System (Applied Biosystems) with an annealing temperature of 60°C and the following primer pairs: *Atad5*-Ex4F (5′-GAC TGA AGA AAC AGT GGT ACC-3′) and *Atad5*-Ex5R (5′-CAA AGA CAG GAA TGG CTG CTC-3′); *Atad5*-Ex14F (5′-GCC TCT TCA CAG CGA AGT GG-3′) and *Atad5*-Ex15R (5′-GTG CTC GCT TCT GCC CAC T-3′); *Atad5*-Ex18F (5′-GTC TAG TGT TTG ATG GCT GCT TTG-3′) and *Atad5*-Ex19R (5′-CAC TTG TAG ATA GCT GGC AAC-3′); *Atad5*-Ex19F (5′-GTT GCC AGC TAT CTA CAA GTG-3′) and *Atad5*-Ex20R (5′-GAG CCA TCT TCT GAA CAA ACC-3′); and LacZR (5′-CTT CGC TAT TAC GCCAGC TGG-3′).

### Protein Analysis

Total cell extract was prepared by sonication of cells in lysis buffer (100 mM Tris-Cl (pH 7.5), 50 mM NaCl, 8% glycerol, and protease inhibitor cocktail (Roche) with either 0.5% Triton X-100 or 0.5% NP40 as a detergent). Supernatant after centrifugation was used as total cell extract. Proteins in total cell extract were separated by electrophoresis using 4–12% gradient polyacrylamide gels (BioRad) and transferred to PVDF membranes (BioRad). Proteins on PVDF membranes were detected by Western blotting with antibodies. Atad5 protein was detected by anti ELG1 antibody raised in rabbits against human N-terminal 1–197 amino acid fragments of ELG1. Fused Elg1 protein was detected by anti-Beta galactosidase antibody (Abcam).

The TritonX-100-insoluble fraction (chromatin bound fraction) was isolated from the Triton X-100-soluble fraction using the traditional methods with slight modifications. Harvested cells were resuspended in buffer A (100 mM NaCl, 300 mM sucrose, 3 mM MgCl2, 10 mM Pipes (pH 6.8), 1 mM EGTA, 0.2% Triton X-100, 100 mM NaVO4, 50 mM NaF, and protease inhibitors (RocheApplied Science)) and incubated for 5 minutes on ice with gentle inverting. The supernatants were recovered as the “soluble fraction” after centrifugation. Followed by washing with buffer A, the pellet was resuspended either in buffer X (100 mM Tris-HCl (pH 8.5), 250 mM NaCl, 1 mM EDTA, 1% Nonidet P-40, 100 mM NaVO4, 50 mM NaF, 2 mg/ml bovine serum albumin, and protease inhibitors) or in buffer B (50 mM Tris-HCl (pH 7.5), 150 mM NaCl, 5 mM EDTA, 1% Triton X-100, 0.1% SDS, 100 mM 100 mM NaVO4, 50 mM NaF, and protease inhibitors) for immunoblotting. Followed by a 10 min incubation on ice, the samples were sonicated and then incubated for another 10 min on ice before centrifugation to isolate the “chromatin-bound fraction.” Immunoblots were stained with PCNA (Santa Cruz) and Rad9 (Cell Signaling) antibodies.

### Generation of *Atad5^m/m^* Mouse Embryos

All embryos were generated by natural mating of *Atad5^+/m^* mice. The morning of the day on which a vaginal plug was detected was designated as day 0.5 p.c. and 3.5 p.c. embryos were collected by flushing uteri with M2 medium (Sigma). To genotype embryos collected at other embryonic days, individual yolk sacs were lysed in 20 µl of 1xPCR lysis buffer with 0.2 mg/ml of proteinase K at 55°C for overnight.

### Chromosomal Abnormalities and Cell Survival Assay

MEFs were treated with the indicated doses of methyl methane sulfonate (MMS) for 2 hours, washed with PBS three times, and incubated in media for one day. Colcemid (0.1 µg/ml) was added to the medium over night before cells were collected. For each cell culture, 50 metaphases were analyzed for chromosomal abnormalities. MEFs were plated in 96 well plates (1,000 cells/well) in triplicate. The following day, cells were treated with MMS at different doses, ranging from 0.00012 to 0.01% for one hour. Cells were washed with PBS and incubated for ten more days. Cell survival was determined using the CyQuant cell proliferation assay kit (Invitrogen).

### Immunofluorescence and Confocal Microscopy

MEF cells were cultured in 2 well Lab Tek chamber slides (Nunc) chamber slide. The next day cells were fixed with 3.7% para-formaldehyde, treated with 0.2% tritonX-100 and stained with phospho-H2AX (α-γ-H2AX; Millipore) and Rad51 (Santa Cruz) antibodies. Fluorescence conjugated anti IgG antibodies (Invitrogen-Molecular Probes) were used as a secondary antibody. Confocal images were collected with a Ziess LSM 510 NLO meta system mounted on a Ziess Axiovert 200 M microscope with an oil immersion Plan-Apochromat X 63/1.4 differential interference contrast objective lens.

### Sister Chromatid Exchange for MEF Cells

MEF cells cultured in 10 cm culture dish for one day were incubated with BrdU for 36 hours. Cells were then treated overnight with Colcemid (0.1 µg/ml). For each cell culture, 50 metaphases were analyzed for sister chromatid exchange abnormalities.

### Cell Cycle Analysis for MEF Cells

MEF cells cultured in 10 cm culture dishes for a day were harvested and washed three times with PBS. Following fixation with 70% ethanol, cells were washed with PBS, stained with PI solution, and kept at 37°C for 30 minutes before sorting. Data were analyzed by ModFIT software (http://www.vsh.com/products/mflt/index.asp).

### Generation of the Heterozygous ATAD5-Deficient Chicken DT40 Cells

The full-length chicken cDNA (DDBJ accession number AB511312) encodes a protein of 1,816 amino acids with 45.6% identity to human *ATAD5*. Partial chicken (ch) *ATAD5* cDNA and genomic sequences were identified by searching databases and cloned by PCR. To knock out *ATAD5* in chicken DT40 cells, a *chATAD5* targeting vector was designed to replace a region that is homologous to the RFC large subunit, RFC1 (PRKO4195) as assigned by conserved domain database with a drug-resistant gene cassette (Figure S1A). The *ATAD5*-targeting vector was created by replacing a 2.7 kb genomic fragment containing 3 exons that correspond to chATAD5 amino acids 1338–1735, with a *his*-resistant gene cassette. DT40 cell cultures, electroporation and subsequent selection, RT-PCR analysis, cell growth determination, colony formation assay were performed as described [44]–[46]. For γ-ray irradiation, ^137^Cs gammacell 40 exactor (MDS Nordion) was used. One allele of *chATAD5* gene was targeted as evidenced by Southern blot (Figure S1B). Semi-quantitative RT-PCR analysis showed that *ATAD5* mRNA levels were decreased to nearly half of the wild type level (Figure S2C). Despite numerous attempts to disrupt the second allele, we were unable to recover homozygous *chATAD5* null DT40 cells. In striking contrast to the targeting efficiency of the first chATAD5 allele ∼10% (3/29), no double positive clones were obtained from 139 transfectants. Thus, we speculate that complete loss of the *chATAD5* gene might be lethal.

### Pulsed-Field Gel Electrophoresis

MEFs were treated with 0.01% MMS for one hour and then allowed to recover for 16 hours. ∼6×10^5^ cells were imbedded into Pulsed-Field Certified Agarose (Bio-Rad) for a final agarose concentration of 0.75%. Agarose plugs were then digested in proteinase K reaction buffer (100 mM EDTA, pH 8.0, 0.2% sodium deoxycholate, 1% sodium lauryl sarcosine, and 1 mg/ml Proteinase K) at 50°C overnight and washed 4 times in wash buffer (20 mM Tris, pH 8.0, 50 mM EDTA). The plugs were loaded onto a 1% Pulsed-Field Certified Agarose gel (Bio-Rad). Separation was performed on a CHEF-DR III pulsed-field electrophoresis system (Bio-Rad; 100° field angle, 1200 s switch time, 2 V/cm, 14°C) for 72 hours. The gel was stained with ethidium bromide.

### Array-Based Comparative Genome Hybridization (CGH) Analysis

Genomic DNA isolated from normal and tumor tissues from the *Atad5^m/+^* mice, using the DNeasy Blood and Tissue Kit (Qiagen, Valencia, CA), was used for array-CGH array analysis assisted by the NHGRI Genomics core. Data analysis was performed by the NHGRI Bioinformatics core. DNAs were hybridized to Whole Mouse Genome CGH Microarrays (105K) (Agilent Technologies) according to the manufacturer's protocol. After hybridization, arrays were washed and scanned. The scanned array images were analyzed and data was extracted using Agilent Feature Extraction (FE) Software with Linear Lowess normalization and background subtraction. Agilent's DNA Analytics Software (Version 4.0.85) was used to identify chromosomal imbalances. The altered chromosomal regions and breakpoints were detected using ADM-1 with threshold 6.0.

### Tissue Microarray Analysis, Pathway Analysis, and Integration of CGH Data with Expression Analysis

Expression profiling was accomplished using the Agilent High Throughput Array (HTA) GeneChip system. All the necessary enzymes and reagents were purchased from the SABiosciences unless specified below. Total RNA was isolated from normal and tumor tissues by guanidinium thiocyanate-phenol-chorofrom extraction using TRIzol reagent (Invitrogen) following the manufacturer's protocol. 600 ng of total RNA from 4 (2 wild-type and 2 tumor) samples were reverse transcribed into cDNA using T_7_dT_24_ primer containing the poly T sequence and the promoter for T7 RNA polymerase. After second strand synthesis, double-stranded cDNA was used as a template to synthesize complementary RNA (cRNA) in a modified “Eberwine” type RNA amplification process. During this amplification process, cRNA was labeled with aminoallyl-UTP (Sigma). 8 µg of aminoallyl-cRNA from each sample was conjugated with Cy3-NHS ester (GE Healthcare) to generate Cy3-labelled cRNA. 1.65 µg Cy3-labelled cRNA generated was fragmented by the cRNA fragmentation reaction and then applied for hybridization. Hybridization was done at 65°C for 17 hours with 4 rpm shaking. The Whole Mouse Genome Oligo Microarray assembly was then dissembled in the hybridization wash buffer 1 at room temperature. The microarray slides were washed with the fresh hybridization wash buffer 1 for 1 minute, followed with the hybridization with the wash buffer 2 for one minute at 37°C, with acetonitrile for 1 minute at room temperature, and with the stabilization and drying solution for 30 seconds. The dried microarray slide was scanned into a TIFF image file on an Agilent microarray scanner at 5 µm resolution using default settings. Microarray intensity data were extracted from the TIFF image using the Agilent Feature Extraction Software 9.1.3. The Feature Extraction Software also processed the signal intensities from Cy3 channel for each microarray. The processed data were then imported into GeneSpring GX 10 (Agilent Technologies) software and normalized using the standard settings for Agilent microarray including per chip global normalization. Global normalization was performed by dividing each measurement by the 50^th^ percentile of all measurements in that sample. The percentile was calculated using only genes marked as present. Data for genes that were absent in all samples and all the control probes were excluded from analyses. Paired t-test were performed to identify a list of statistically significant (P<0.05) differentially expressed genes between normal and tumor samples with a cut-off criteria of expression differences greater than or equal to 2-fold. Each differentially expressed gene was classified according to its Gene Ontology (GO), in which genes are organized into hierarchical categories based on biological process, molecular function and cellular compartment. GO term analysis was performed using Genespring GX 10 software. To determine the functional relationships among the identified genes, differentially expressed genes were imported into Ingenuity Pathway Analysis software (IPA) (http://www.ingenuity.com). IPA contains the most literature knowledge of biological interactions among the gene products. This web-based software generated a set of molecular network based on interaction between uploaded genes and all other genes present in the knowledge base. In order to compare the measurements obtained from CGH array analysis and microarray expression data, the genes that were present in regions of amplification and deletion detected by array-CGH were chosen to determine their correlation in microarray expression. The goal of this analysis was to detect genes where a copy number change is correlated with the change in expression. Multiple public expression database, Oncomine (https://www.oncomine.org/resource/login.html) was manually interrogated for mouse *Atad5* and its aliases. Only studies comparing normal against tumor tissues were queried for this gene.

### Nucleic Acid Isolation

Genomic DNA was isolated from macrodissected tissue with greater than 70% tumor purity using the Puregene kit (Qiagen). Total RNA was isolated with TRIzol (Invitrogen), according to the manufacturer's instructions. cDNA was synthesized using the Super Script III First Strand Synthesis System (Invitrogen).

### Human Cell Line Culture and Transfection

Human embryonic kidney (HEK) 293T cells were maintained in Dulbecco's modified Eagle's medium (DMEM) with 10% fetal bovine serum (FBS) (Hyclone), 100 U/mL penicillin G and 100 µg/mL streptomycin. Transfections were carried out using Lipofectamine2000 (Invitrogen) and cells were incubated for 48 h prior to harvesting. For RNA interference, transfection was performed two times with an interval of 24 h.

### DNA Constructs and siRNA

Full-length *ATAD5* cDNA was cloned into p3XFLAG CMV10 expression vector and used as a template for site-directed mutagenesis with the QuickChange Site-Directed Mutagenesis kit (Stratagene). Construct integrity was verified by nucleotide sequencing. siRNAs (Dharmacon) to target the 3′ untranslated region (Utr) of *ATAD5* were: 5′- GUAUAUUUCUCGAUGUAC A-3′ (sense) and 5′- UGUACAUCGAGAAAUAUACUU -3′ (antisense).

### Immunoprecipitation and Immunoblotting

Triton X-100 soluble and insoluble fractions were isolated using a modified version of the method described by Kannouche et al [47]. For immunoprecipitation, proteins were pre-cleared with protein G Sepharose beads (GE Healthcare), incubated with specific antibodies, and then the complex recognized by antibodies was precipitated with protein G Sepharose beads. Proteins or immunoprecipitated samples were resolved on NuPAGE Novex 4–12% Bis-Tris Gel (Invitrogen) and transferred to PVDF membranes (Bio-Rad), followed by immunoblotting.

### Quantitative Reverse Transcription and Real-Time PCR

Total RNAs (500 ng), prepared using TRIzol (Invitrogen) were subjected to qRT-PCR using the SuperScriptIII Platinum Two Step qRT-PCR kit (Invitrogen) and a 7300 Real Time PCR system (Applied Biosystems). Primer sequences used for qRT-PCR were: ATAD5, 5′-CGG AGA CGA AGA AAG CAA AG-3′ (sense) and 5′-CAA TGA GAA ACA AGG GCA GA-3′ (antisense), β-actin, 5′-GCT CGT CGT CGA CAA CGG CTC-3′ (sense) and 5′-CAA ACA TGA TCT GGG TCA TCT TCT C-3′ (antisense). β-actin expression served as an internal control.

### PCR Amplification of ATAD5 from Clinical Samples

PCR was performed in a total volume of 10 µl containing 5 ng genomic DNA, 0.16 µM sense primer, 0.16 µM antisense primer, and Immomix (Bioline). PCR amplification was performed on MBS Satellite 384 Thermalcyclers (Thermo Scientific). Cycling conditions were 95°C for 3 min followed by 40 cycles of 95°C for 15 s; 60°C for 15 s; 72°C, 1 min; followed by a final extension of 72°C for 5 min.

### Nucleotide Sequencing of *ATAD5* from Clinical Samples

Sequence trace quality was assessed with the base-calling program, Phred [48]–[50]. All traces were included in the subsequent analysis, since deletion-insertion polymorphisms can mimic poor quality data from a Phred-quality measure, but may contain valid sequence data. All sequences for a given primer pair were assembled using Consed [50]; overlapping amplimers were assembled separately to allow independent cross validation of calls in overlapping regions. Sequence variants, including single-nucleotide differences and short (<100 base pair) insertions and deletions, were identified using PolyPhred v6.11 [51] and DIPDetector, an insertion-deletion (indel) detector optimized for improved sensitivity in finding insertions and deletions from aligned trace data. Since a heterozygous insertion or deletion produces traces that are superpositions of two alleles of different length, DIPDetector is able to use Phred's primary and secondary peak predictions at each base position to calculate an autocorrelation function of the trace with itself shifted by different potential insertion or deletion sizes. A maximum in the value of this function with respect to shift size indicates a potential indel. To improve accuracy, DIPDetector also examines the trace alignments for homozygous insertions and deletions, and uses these homozygous genotypes to refine its prediction for the insertion or deletion position, resulting in greater accuracy of positions when compared to PolyPhred. Human genome assembly hg18 (NCBI Build 36.1) was used as the reference sequence. Variant positions were cross-referenced to dbSNP (Build 129) entries to identify known polymorphisms. To determine whether novel variants were somatic mutations or germline polymorphisms, the appropriate tumor DNA and matched normal DNA were re-amplified in an independent PCR followed by sequence analysis of the variant position.

### Laser Capture Microdissection and RT-PCR

Frozen tissue sections (8 µM) of OCT-embedded primary tumors were prepared at American HistoLabs Inc., (Gaithersburg MD), and were H&E stained immediately prior to microdissection. Approximately 4000–5000 laser pulses were performed using the following infrared laser parameters: laser spot size 7.5 µm, power 70 mW, duration 950 µs. Each tissue section and the subsequent dissection was reviewed by a staff pathologist. RNA was isolated from LCM samples using the Arcturus Picopure RNA isolation kit (MDS Analytical Technologies). RNA (50–200 ng) was converted to cDNA using SuperScript III First-Strand Synthesis System (Invitrogen). RT-PCR primers were designed to span intronic boundaries to avoid amplification of any contaminating genomic DNA. Primer sequences (5′-3′) were: CTT TTT GAG GAG GTT GAT G (sense) and GTC CAA GTC AGT GTC AAA TAG ATC AC (antisense). Purified RT-PCR products were subjected to bidirectional sequencing to determine whether there was monoallelic or biallelic expression of mutant *ATAD5* alleles.

## Supporting Information

Figure S1 Establishment of heterozygous *ATAD5*-deficient chicken DT40 cells by targeted disruption. (A) Schematic representation of part of the chicken (ch) *ATAD5* locus, the gene targeting constructs, and the configuration of the targeted allele. The black box indicates the position of exons. B, *BamHI* site. (B) Southern blot analysis of *BamHI*-digested genomic DNA from DT40 cells of the indicated genotypes, using a flanking probe as shown in panel A. (C) Semi-quantitative RT-PCR analysis of *ATAD5* mRNA expression. cDNAs prepared from wild-type and two heterozygous cells (Clones 1 and 2) were serially diluted and subjected to PCR amplification using primers shown as open triangles in panel A. The gel picture is shown in the left panel. N, no cDNA template. GAPDH was also amplified as control. Densitometric analysis of *ATAD5* band intensity was carried out using Image J software and plotted in the right panel. (D) Survival of DT40 cells of the indicated genotypes was evaluated by colony survival in medium containing methylcellulose after exposure to increasing doses of γ-irradiation. Error bars indicate the standard deviation from at least three experiments. (E) Survival of chicken DT40 cells with indicated genotypes was evaluated by colony survival in medium containing methylcellurose by continuous exposure to different doses of MMS. Error bars indicate standard deviation from at least three experiments. * and ** indicate statistical significance of p<0.05 and p<0.01, respectively by the Student's t-test. (F) Chromosome analysis of DT40 cells with the indicated genotypes. Cells were γ-ray irradiated (+, 2Gy) or left untreated (−, 0 Gy) and sampled at indicated time points. One hundred metaphases were scored blindly for each preparation as described [44]. Error bars indicated standard deviations. FANCD2 deficient DT40 cells were used as a control [52].
(TIF)

Figure S2 MEFs derived from the *Atad5^+/m^* mice have molecular defects in suppression of PCNA ubiquitination. The level of PCNA ubiquitination in response to 0.01% MMS treatment was compared between wild-type and *Atad5^+/m^* MEFs. Bottom table shows the levels of PCNA and Ubiquitinated PCNA (Ub-PCNA) quantified by comparing to Histone H3. The level of Ub-PCNA quantified by comparing to PCNA is in the same table.
(TIF)

Figure S3 Differential mRNA expression in tumors from *Atad5^+/m^* mice. (A) Results of unsupervised hierarchical clustering of two tumors from *Atad5^+/m^* animals. Red indicates increased gene expression and green indicates decreased gene expression in tumors (two right hand columns) relative to controls provided by surrounding tissues (two left hand columns). (B, C) Quantitative RT-PCR analysis confirmed the expression changes for (B) four genes that exhibited increased expression by microarray analysis and (C) three genes that exhibited decreased expression by microarray analysis.
(TIF)

Figure S4 The ATAD5-R1414X somatic mutation is heterozygous in endometrial tumor T51. (A) Sequence trace of matched germline DNA surrounding *ATAD5* codon 1414 (boxed). Only the wild type sequence is detectable. (B) Sequence trace of genomic PCR products generated from macrodissected tumor tissue from T51. Both wildtype and mutant bases are detected at codon 1414 (arrow). (C) Sequence trace of RT-PCR products generated from pure populations of tumor cells obtained by laser capture microdissection of tumor T51. Both wildtype and mutant bases are detected at codon 1414 (arrow).
(TIF)

Table S1 Regions of copy number alteration and the corresponding genes among four tumors from *Atad5^+/m^* mice.
(PDF)

Table S2 List of cancer gene transcripts that had up or down regulated expression in two tumor samples compared to their normal controls.
(PDF)

Table S3 Genes that showed both altered expression and copy number in murine tumors.
(PDF)

Table S4 Primers used to PCR amplify human *ATAD5*.
(TIF)
