# Supplementary figures and images for: Predisposition to Cancer Caused by Genetic and Functional Defects of Mammalian Atad5
Source: PLoS Genet. 2011 Aug 25;7(8):e1002245. doi: 10.1371/journal.pgen.1002245 (PMC3161924; doi:10.1371/journal.pgen.1002245)

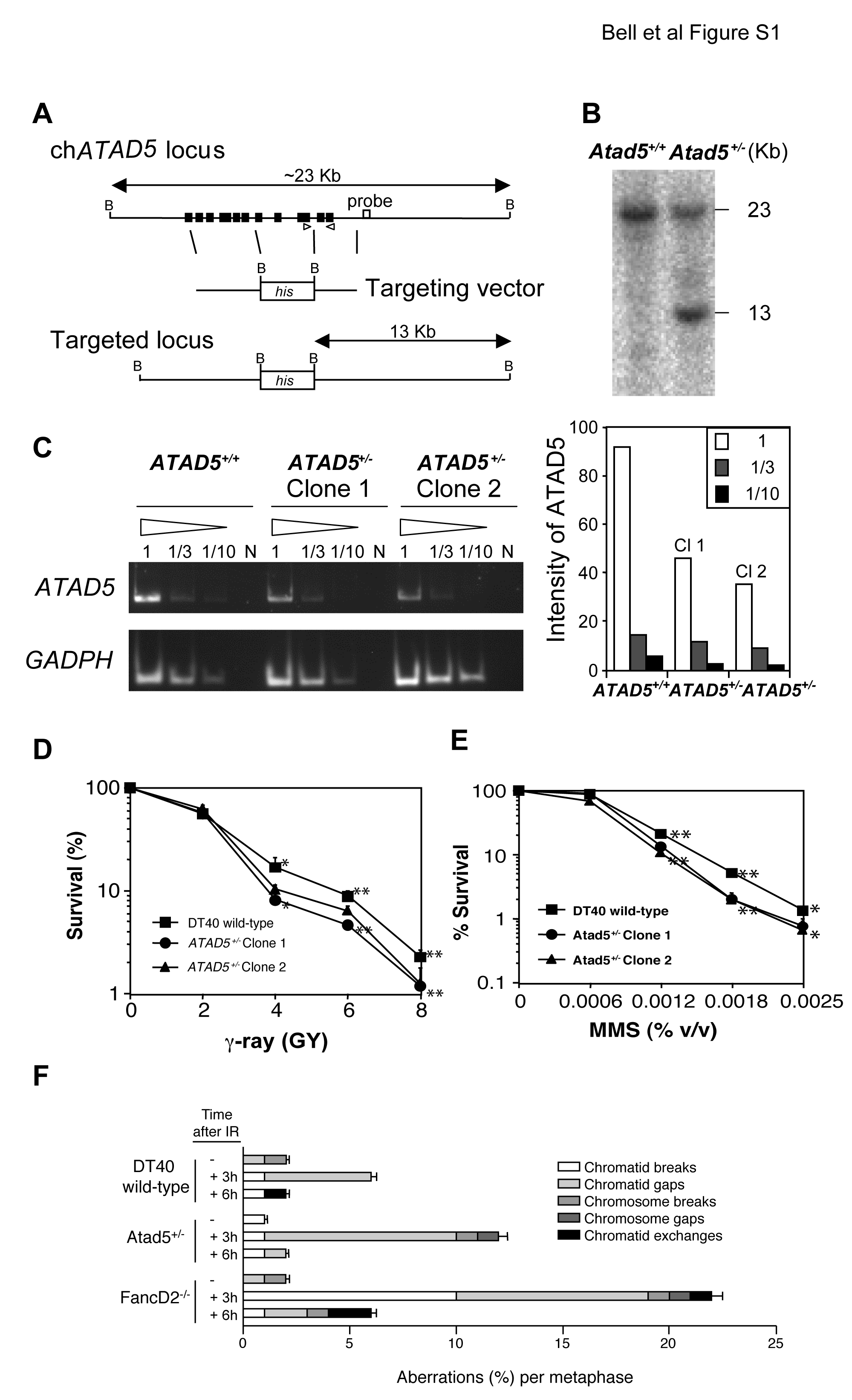

Supplement: Figure S1 — Establishment of heterozygous ATAD5-deficient chicken DT40 cells by targeted disruption. (A) Schematic representation of part of the chicken (ch) ATAD5 locus, the gene targeting constructs, and the configuration of the targeted allele. The black box indicates the position of exons. B, BamHI site. (B) Southern blot analysis of BamHI-digested genomic DNA from DT40 cells of the indicated genotypes, using a flanking probe as shown in panel A. (C) Semi-quantitative RT-PCR analysis of ATAD5 mRNA expression. cDNAs prepared from wild-type and two heterozygous cells (Clones 1 and 2) were serially diluted and subjected to PCR amplification using primers shown as open triangles in panel A. The gel picture is shown in the left panel. N, no cDNA template. GAPDH was also amplified as control. Densitometric analysis of ATAD5 band intensity was carried out using Image J software and plotted in the right panel. (D) Survival of DT40 cells of the indicated genotypes was evaluated by colony survival in medium containing methylcellulose after exposure to increasing doses of γ-irradiation. Error bars indicate the standard deviation from at least three experiments. (E) Survival of chicken DT40 cells with indicated genotypes was evaluated by colony survival in medium containing methylcellurose by continuous exposure to different doses of MMS. Error bars indicate standard deviation from at least three experiments. * and ** indicate statistical significance of p<0.05 and p<0.01, respectively by the Student's t-test. (F) Chromosome analysis of DT40 cells with the indicated genotypes. Cells were γ-ray irradiated (+, 2Gy) or left untreated (−, 0 Gy) and sampled at indicated time points. One hundred metaphases were scored blindly for each preparation as described [44]. Error bars indicated standard deviations. FANCD2 deficient DT40 cells were used as a control [52]. (TIF) [file pgen.1002245.s001.tif]

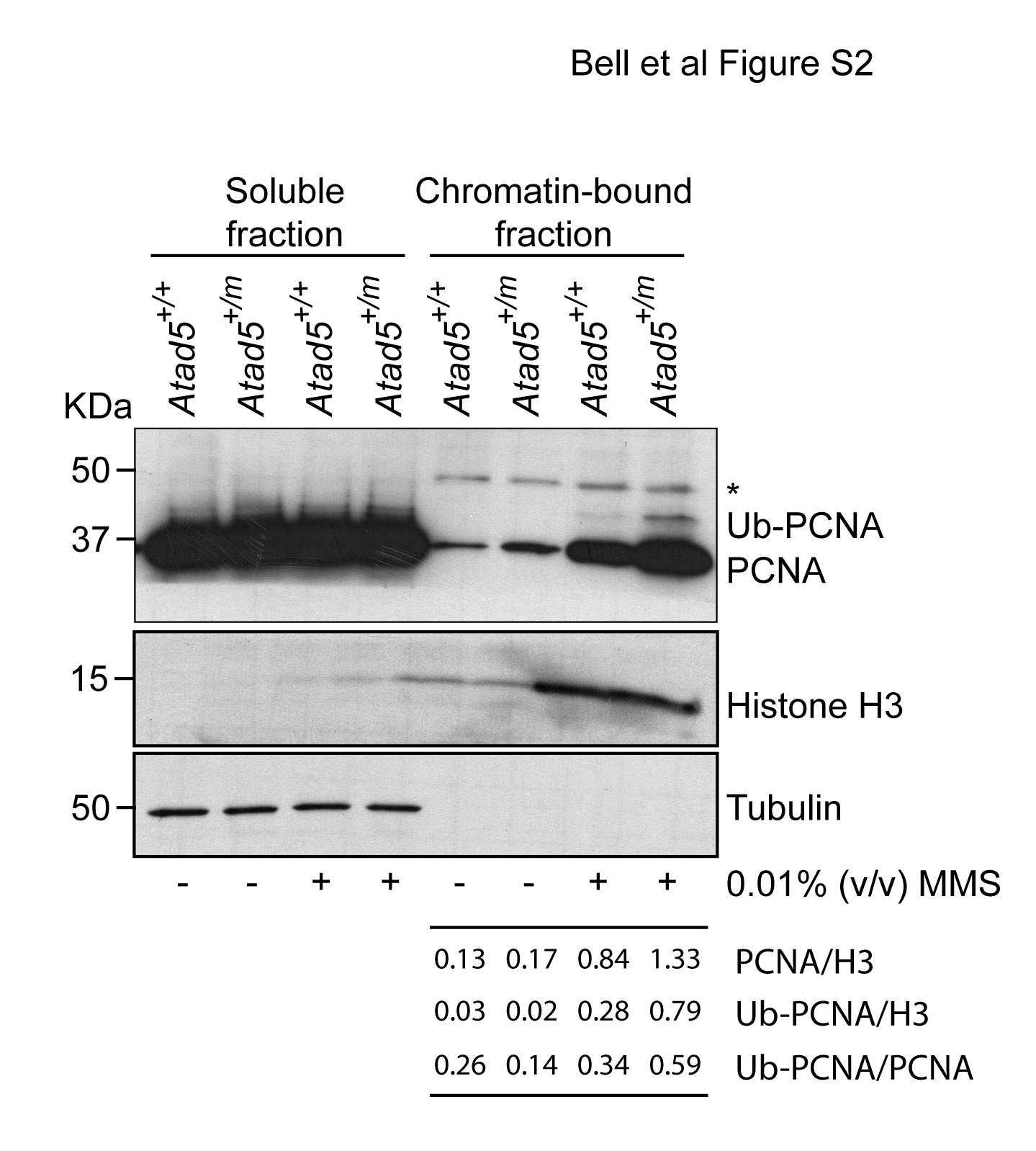

Supplement: Figure S2 — MEFs derived from the Atad5+/m mice have molecular defects in suppression of PCNA ubiquitination. The level of PCNA ubiquitination in response to 0.01% MMS treatment was compared between wild-type and Atad5+/m MEFs. Bottom table shows the levels of PCNA and Ubiquitinated PCNA (Ub-PCNA) quantified by comparing to Histone H3. The level of Ub-PCNA quantified by comparing to PCNA is in the same table. (TIF) [file pgen.1002245.s002.tif]

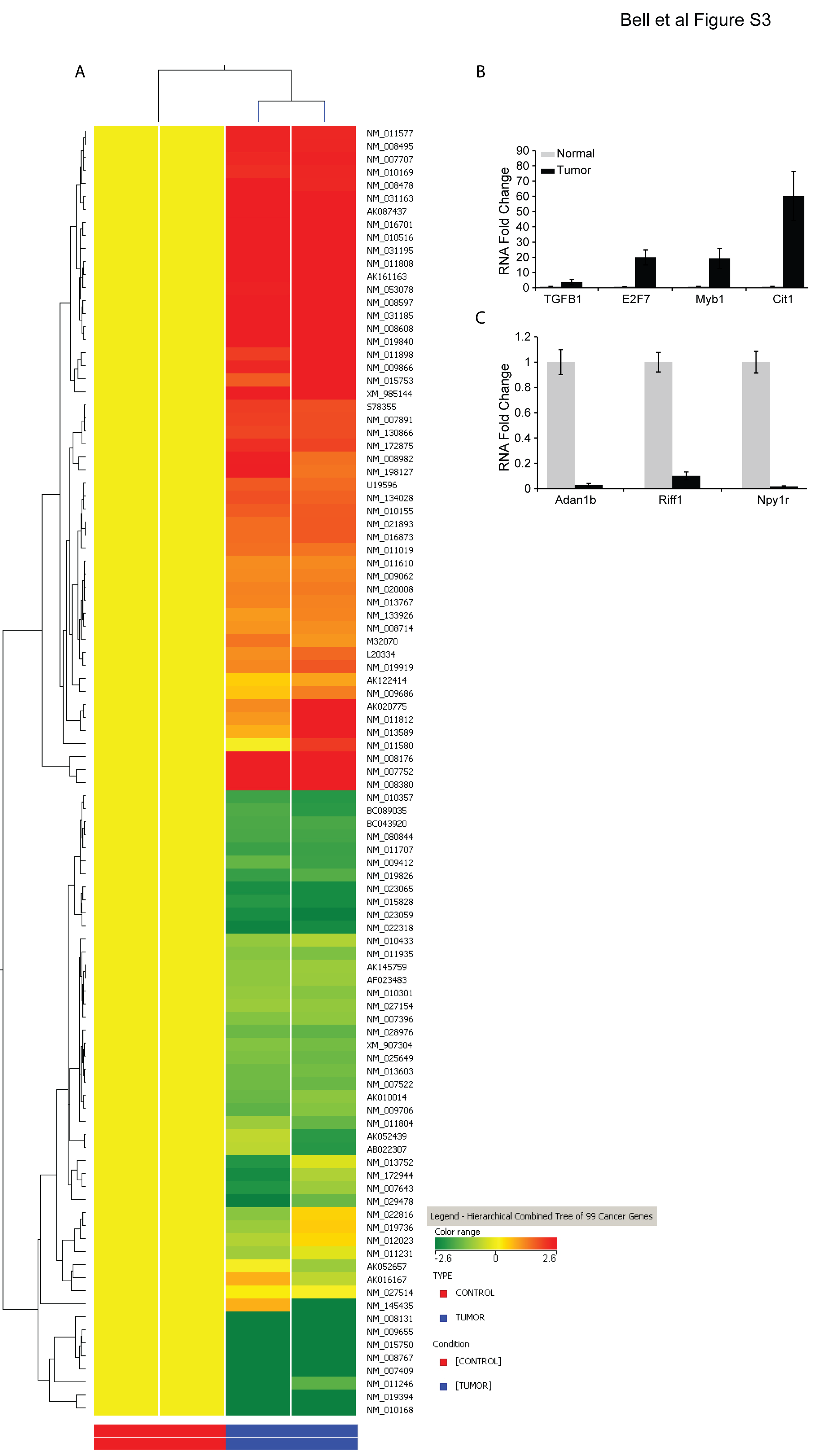

Supplement: Figure S3 — Differential mRNA expression in tumors from Atad5+/m mice. (A) Results of unsupervised hierarchical clustering of two tumors from Atad5+/m animals. Red indicates increased gene expression and green indicates decreased gene expression in tumors (two right hand columns) relative to controls provided by surrounding tissues (two left hand columns). (B, C) Quantitative RT-PCR analysis confirmed the expression changes for (B) four genes that exhibited increased expression by microarray analysis and (C) three genes that exhibited decreased expression by microarray analysis. (TIF) [file pgen.1002245.s003.tif]

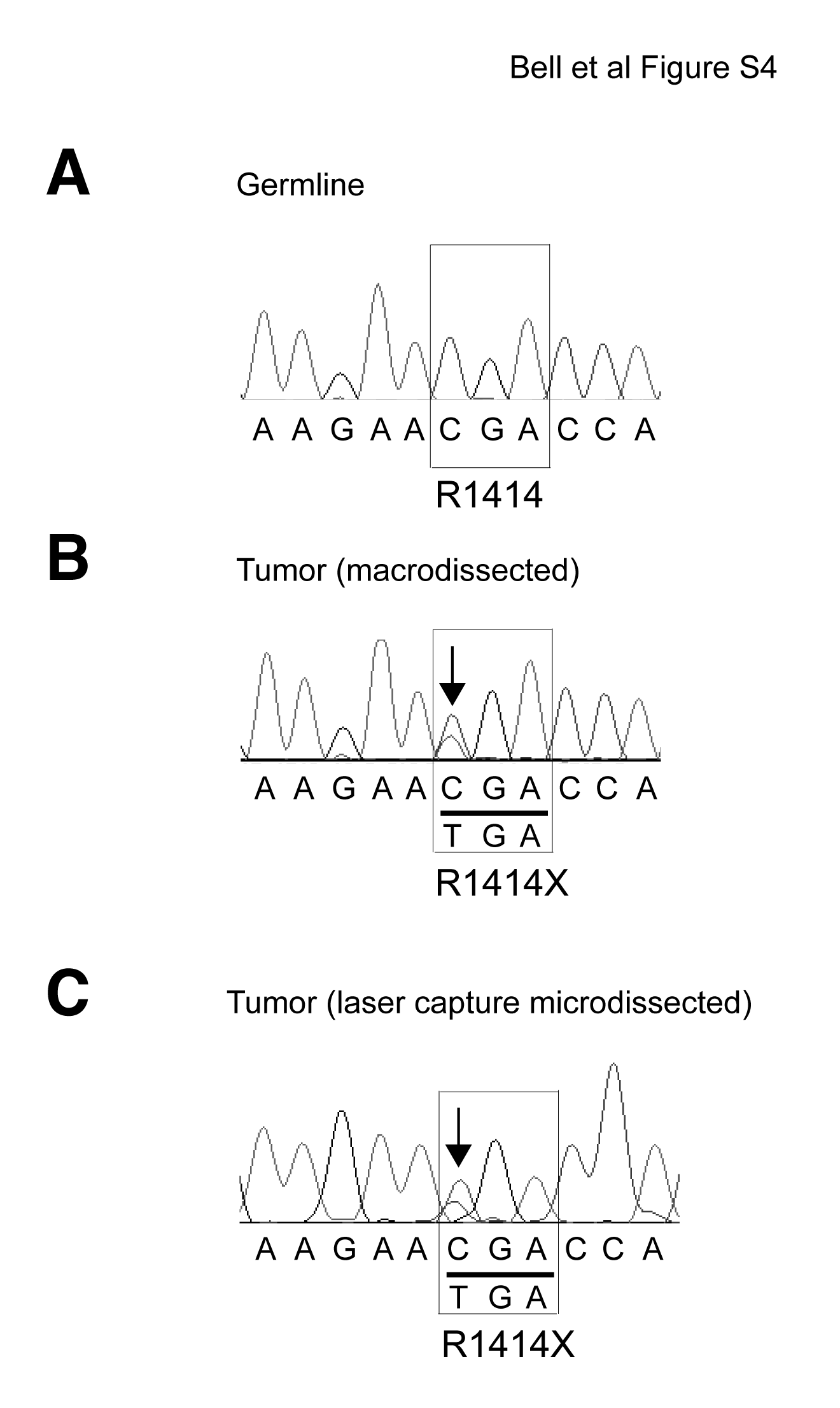

Supplement: Figure S4 — The ATAD5-R1414X somatic mutation is heterozygous in endometrial tumor T51. (A) Sequence trace of matched germline DNA surrounding ATAD5 codon 1414 (boxed). Only the wild type sequence is detectable. (B) Sequence trace of genomic PCR products generated from macrodissected tumor tissue from T51. Both wildtype and mutant bases are detected at codon 1414 (arrow). (C) Sequence trace of RT-PCR products generated from pure populations of tumor cells obtained by laser capture microdissection of tumor T51. Both wildtype and mutant bases are detected at codon 1414 (arrow). (TIF) [file pgen.1002245.s004.tif]

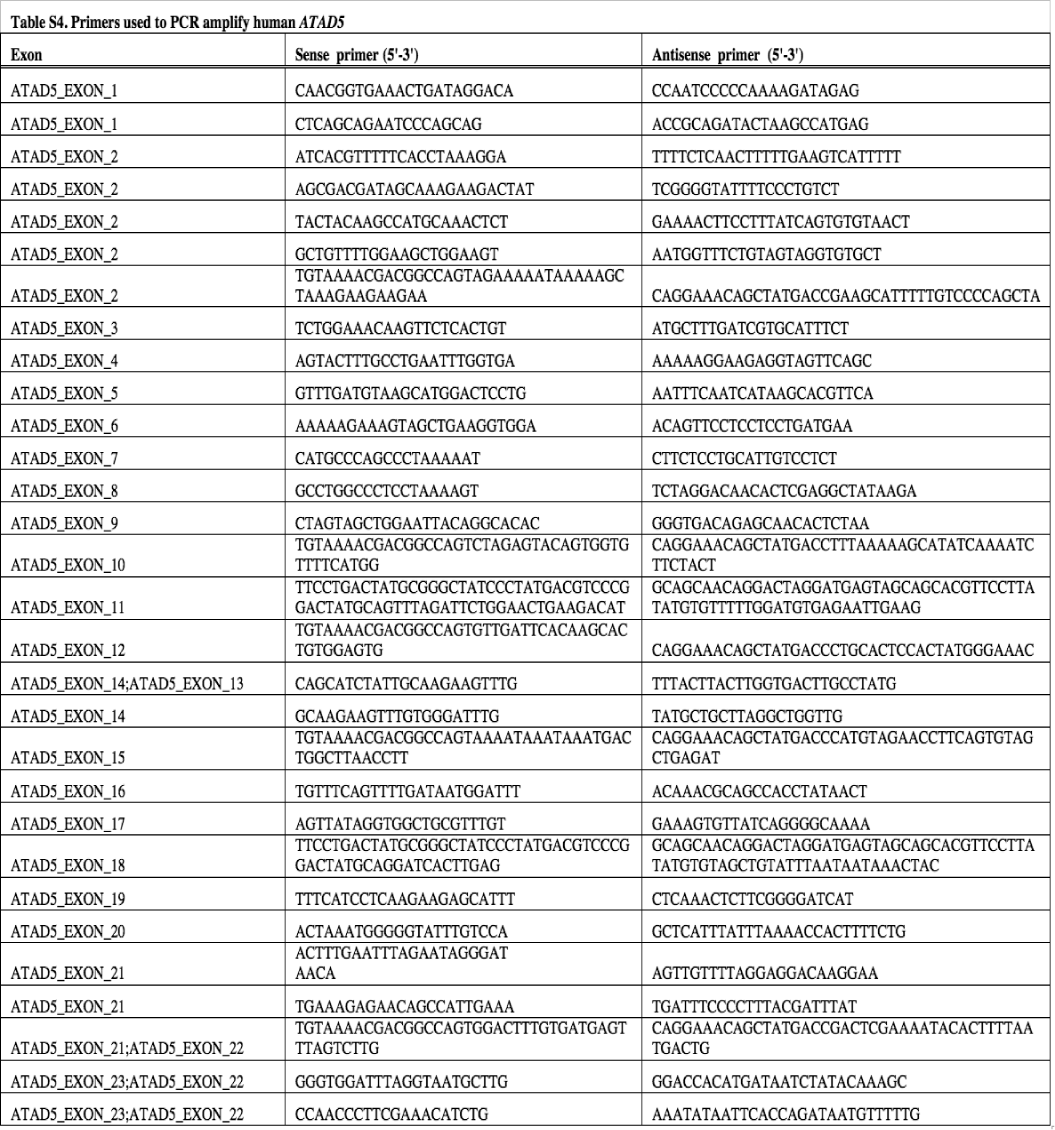

Supplement: Table S4 — Primers used to PCR amplify human ATAD5. (TIF) [file pgen.1002245.s008.tif]
