# Supplementary material for: Predisposition to Cancer Caused by Genetic and Functional Defects of Mammalian Atad5
Source: PLoS Genet. 2011 Aug 25;7(8):e1002245. doi: 10.1371/journal.pgen.1002245 (PMC3161924; doi:10.1371/journal.pgen.1002245)
Supplement: Table S1 — Regions of copy number alteration and the corresponding genes among four tumors from Atad5+/m mice. (PDF) [file pgen.1002245.s005.pdf]

Table S1. Regions of copy number alteration and the corresponding genes among four tumors from Atad5+/m mice.

ChrNumber	ProbeName	Start	Stop	Deletion	Amplification
chr1	A_67_P04010830	9884081	9884140	0	25
chr1	A_53_P127556	17723913	17723972	0	25
chr1	A_53_P127556	17723913	17723972	0	25
chr1	A_53_P127556	17723913	17723972	0	25
chr1	A_53_P127501	17741507	17741566	0	25
chr1	A_67_P04055333	32621242	32621301	25	0
chr1	A_53_P163710	36210559	36210618	0	25
chr1	A_53_P111377	39494205	39494264	0	25
chr1	A_67_P04111240	58801090	58801149	0	25
chr1	A_67_P00100290	72235377	72235436	25	0
chr1	A_67_P04212616	106703566	106703625	25	0
chr1	A_67_P04316715	157349593	157349652	50	0
chr1	A_67_P00238028	165814781	165814840	0	25
chr1	A_67_P00247956	171555916	171555975	0	25
chr1	A_67_P04379846	184316327	184316386	0	25
chr2	A_53_P122881	4900498	4900557	0	25
chr2	A_53_P140447	29688284	29688338	25	0
chr2	A_53_P140339	29690643	29690702	25	0
chr2	A_53_P126333	29694550	29694604	25	0
chr2	A_53_P149149	30007128	30007187	0	25
chr2	A_53_P142959	38182019	38182069	25	0
chr2	A_53_P142959	38182019	38182069	25	0
chr2	A_53_P142959	38182019	38182069	25	0
chr2	A_67_P04497742	42163870	42163929	50	0
chr2	A_53_P124470	65156156	65156214	0	25
chr2	A_67_P04546499	65179814	65179873	0	25
chr2	A_67_P04546499	65179814	65179873	0	25
chr2	A_67_P04546499	65179814	65179873	0	25
chr2	A_67_P04610311	93668807	93668866	25	0
chr2	A_53_P143963	102910708	102910767	25	0
chr2	A_53_P130345	127057207	127057258	0	50
chr2	A_53_P165807	127057461	127057519	0	50
chr2	A_53_P146037	127058196	127058246	0	50
chr2	A_53_P142604	127078793	127078852	0	50
chr2	A_53_P142604	127078793	127078852	0	50
chr2	A_53_P142604	127078793	127078852	0	50
chr2	A_53_P175034	132537535	132537594	0	25
chr2	A_53_P122355	132546377	132546436	0	25
chr2	A_53_P108325	132555577	132555636	0	25
chr2	A_67_P04754545	157053447	157053506	25	0
chr2	A_53_P133949	167875056	167875115	25	0
chr2	A_53_P138401	167892101	167892160	25	0
chr2	A_53_P137188	167895601	167895660	25	0
chr2	A_53_P137188	167895601	167895660	25	0
chr2	A_53_P137188	167895601	167895660	25	0
chr2	A_53_P173360	167901807	167901866	25	0
chr2	A_53_P130759	172627871	172627930	50	0
chr2	A_53_P150980	172634873	172634932	50	0
chr2	A_53_P119738	172645445	172645504	50	0
chr2	A_53_P124390	181599311	181599370	25	0
chr3	A_53_P165905	67061180	67061239	25	0
chr3	A_53_P165905	67061180	67061239	25	0
chr3	A_53_P165905	67061180	67061239	25	0
chr3	A_53_P111210	67063507	67063566	25	0
chr3	A_53_P144597	67068194	67068253	25	0
chr3	A_53_P168889	105097650	105097709	25	0
chr3	A_67_P05025592	108722263	108722322	0	25
chr3	A_53_P104868	127553166	127553225	0	25
chr3	A_53_P153389	127554177	127554236	0	25
chr3	A_53_P119858	127578083	127578142	0	25
chr3	A_53_P119858	127578083	127578142	0	25
chr3	A_53_P119858	127578083	127578142	0	25
chr4	A_53_P171194	6871304	6871363	25	0
chr4	A_53_P117023	25292463	25292522	0	25
chr4	A_53_P158856	108075973	108076032	25	0
chr4	A_53_P153893	116145076	116145135	0	25
chr4	A_53_P175041	116148266	116148325	0	25
chr4	A_53_P119710	116189464	116189523	0	25
chr4	A_53_P119710	116189464	116189523	0	25
chr4	A_53_P119710	116189464	116189523	0	25
chr4	A_53_P141513	116201120	116201179	0	25
chr4	A_67_P00991319	131533937	131533996	25	0
chr4	A_53_P153557	131547829	131547888	25	0
chr4	A_53_P179626	135292419	135292478	0	25
chr4	A_53_P127264	143718531	143718590	25	0
chr4	A_53_P127264	143718531	143718590	25	0
chr4	A_53_P127264	143718531	143718590	25	0
chr4	A_53_P176506	143718719	143718778	25	0
chr4	A_53_P159738	143719730	143719789	25	0
chr4	A_53_P135204	143727277	143727336	25	0
chr4	A_53_P149697	154036955	154037014	25	0
chr5	A_67_P01045598	17326606	17326665	25	0
chr5	A_53_P126590	23170604	23170663	0	25
chr5	A_53_P126590	23170604	23170663	0	25
chr5	A_53_P126590	23170604	23170663	0	25
chr5	A_53_P138609	23193207	23193266	0	25
chr5	A_53_P135438	33414961	33415020	0	25
chr5	A_67_P01077607	38089212	38089271	25	0
chr5	A_53_P108165	38108535	38108594	25	0
chr5	A_53_P175486	38109073	38109119	25	0

chr5	A_53_P123082	38109346	38109390	25	0
chr5	A_53_P162874	38111207	38111260	25	0
chr5	A_53_P173329	38113363	38113417	25	0
chr5	A_53_P128190	66556144	66556203	0	25
chr5	A_53_P171803	96198914	96198970	0	25
chr5	A_53_P174588	96211239	96211298	0	25
chr5	A_53_P167185	111078276	111078335	0	25
chr5	A_53_P122821	114719268	114719327	0	25
chr5	A_53_P168074	119026225	119026284	0	25
chr5	A_53_P124387	124864339	124864398	0	25
chr5	A_53_P172781	125678195	125678254	25	0
chr5	A_67_P05743100	130129644	130129703	25	0
chr5	A_53_P137728	136404662	136404715	25	0
chr6	A_53_P176344	18046851	18046910	25	0
chr6	A_53_P161420	40379374	40379430	25	0
chr6	A_53_P133411	51379645	51379704	0	25
chr6	A_53_P107943	51386191	51386250	0	25
chr6	A_53_P116958	53749223	53749268	0	25
chr6	A_53_P163759	53751299	53751358	0	25
chr6	A_67_P05912680	58269139	58269198	25	0
chr6	A_53_P142840	64648433	64648492	0	25
chr6	A_67_P01346282	64649017	64649076	0	25
chr6	A_67_P05924178	64668172	64668231	0	25
chr6	A_53_P166406	64669896	64669953	0	25
chr6	A_53_P177216	64671111	64671170	0	25
chr6	A_67_P05928394	66896082	66896141	0	25
chr6	A_53_P159421	81891515	81891574	0	25
chr6	A_53_P121320	83736104	83736163	25	0
chr6	A_53_P140954	90314274	90314333	0	25
chr6	A_53_P166377	90439821	90439880	0	25
chr6	A_53_P132201	92026420	92026476	0	25
chr6	A_53_P163911	92041255	92041314	0	25
chr6	A_53_P145850	92042425	92042484	0	25
chr6	A_53_P125111	113504292	113504351	0	25
chr6	A_53_P102294	113504962	113505021	0	25
chr6	A_53_P121700	114398210	114398269	0	25
chr6	A_53_P121700	114398210	114398269	0	25
chr6	A_53_P121700	114398210	114398269	0	25
chr6	A_53_P163903	115742122	115742178	0	25
chr6	A_53_P163903	115742122	115742178	0	25
chr6	A_53_P163903	115742122	115742178	0	25
chr6	A_53_P165299	122301705	122301764	0	25
chr6	A_53_P130502	129363053	129363112	0	25
chr6	A_53_P170910	132533042	132533101	0	25
chr6	A_53_P110677	132536497	132536556	0	25
chr6	A_67_P06090397	140487543	140487602	0	50
chr6	A_67_P06090397	140487543	140487602	0	50
chr6	A_67_P06090397	140487543	140487602	0	50
chr7	A_53_P175668	19185030	19185089	0	50
chr7	A_53_P161898	19188507	19188566	0	50
chr7	A_53_P136562	30274044	30274092	25	0
chr7	A_53_P136316	30276685	30276741	25	0
chr7	A_53_P167236	30287447	30287496	25	0
chr7	A_53_P163206	44394346	44394391	25	0
chr7	A_53_P113508	44419097	44419156	25	0
chr7	A_53_P107873	45558921	45558977	25	0
chr7	A_53_P119967	47474727	47474786	0	50
chr7	A_53_P147158	102452475	102452519	25	0
chr7	A_53_P149239	102461767	102461815	25	0
chr7	A_67_P06334011	115801929	115801988	25	0
chr7	A_67_P06385766	136789565	136789624	0	25
chr7	A_53_P114561	141202973	141203032	0	25
chr7	A_53_P114840	141312737	141312796	25	0
chr7	A_53_P124983	141321624	141321668	25	0
chr7	A_53_P158538	141324859	141324909	25	0
chr7	A_53_P101441	141325525	141325576	25	0
chr7	A_53_P147986	141325836	141325882	25	0
chr7	A_53_P120897	141331624	141331683	25	0
chr7	A_53_P129660	141336674	141336733	25	0
chr8	A_53_P140537	10006559	10006617	0	25
chr8	A_53_P119390	23895107	23895166	25	0
chr8	A_53_P104738	55600809	55600868	25	0
chr8	A_53_P104738	55600809	55600868	25	0
chr8	A_53_P104738	55600809	55600868	25	0
chr8	A_67_P06529067	63773877	63773936	25	0
chr8	A_67_P06540414	69431029	69431088	0	25
chr8	A_67_P06540813	69645876	69645935	0	25
chr8	A_67_P01761875	72688021	72688080	25	25
chr8	A_53_P153143	72697767	72697826	25	25
chr8	A_53_P153143	72697767	72697826	25	25
chr8	A_53_P153143	72697767	72697826	25	25
chr8	A_53_P126910	72698917	72698976	25	25
chr8	A_53_P156271	72700060	72700119	25	25
chr8	A_53_P156271	72700060	72700119	25	25
chr8	A_53_P156271	72700060	72700119	25	25
chr8	A_67_P06546672	72721301	72721360	50	25
chr8	A_67_P06558081	78030417	78030476	25	0
chr8	A_53_P123989	86323615	86323674	0	25
chr8	A_53_P157640	86328704	86328763	0	25
chr8	A_53_P103525	86870864	86870912	25	0
chr8	A_67_P01785026	88298941	88299000	25	0
chr8	A_53_P169689	88308075	88308134	25	0

chr8	A_53_P133367	88310330	88310389	25	0
chr8	A_53_P130056	97113053	97113112	25	25
chr8	A_53_P130056	97113053	97113112	25	25
chr8	A_53_P130056	97113053	97113112	25	25
chr8	A_53_P134681	97122574	97122633	25	0
chr8	A_53_P134681	97122574	97122633	25	0
chr8	A_53_P134681	97122574	97122633	25	0
chr8	A_53_P151421	97977104	97977163	25	0
chr8	A_53_P117960	97994145	97994204	25	0
chr8	A_53_P128807	108158406	108158465	0	25
chr8	A_53_P139728	108163589	108163647	0	25
chr8	A_67_P06642295	114380612	114380671	25	0
chr8	A_67_P06673097	126685325	126685384	25	0
chr8	A_53_P170387	126703183	126703242	25	0
chr8	A_53_P125806	127759543	127759602	25	0
chr8	A_53_P132183	127779173	127779232	25	0
chr8	A_53_P132183	127779173	127779232	25	0
chr8	A_53_P132183	127779173	127779232	25	0
chr8	A_53_P120427	127779694	127779753	25	0
chr8	A_53_P101229	127781274	127781333	25	0
chr8	A_53_P120650	127796302	127796361	25	0
chr9	A_53_P139458	20867691	20867747	25	0
chr9	A_53_P150466	21023842	21023901	25	0
chr9	A_53_P162938	21023844	21023902	25	0
chr9	A_67_P01881858	21034772	21034831	25	0
chr9	A_67_P01882231	21243514	21243573	25	0
chr9	A_53_P126751	21830204	21830263	25	0
chr9	A_53_P173333	39159483	39159542	25	0
chr9	A_53_P125743	46020556	46020615	25	0
chr9	A_67_P06818387	63908740	63908799	25	0
chr9	A_67_P01969536	72476042	72476101	0	25
chr9	A_53_P158355	100757025	100757084	25	25
chr9	A_53_P158355	100757025	100757084	25	25
chr9	A_53_P158355	100757025	100757084	25	25
chr9	A_53_P162858	100766218	100766277	0	25
chr9	A_53_P162858	100766218	100766277	0	25
chr9	A_53_P162858	100766218	100766277	0	25
chr9	A_53_P112402	100781741	100781800	25	25
chr9	A_53_P117142	108240429	108240488	0	25
chr10	A_67_P06966003	6571112	6571171	25	0
chr10	A_67_P06966068	6595657	6595716	25	0
chr10	A_67_P06966068	6595657	6595716	25	0
chr10	A_67_P06966068	6595657	6595716	25	0
chr10	A_67_P06974685	10521792	10521851	50	0
chr10	A_67_P06974704	10537281	10537340	50	0
chr10	A_67_P06974704	10537281	10537340	50	0
chr10	A_67_P06974704	10537281	10537340	50	0
chr10	A_53_P180466	51444579	51444638	0	25
chr10	A_53_P120731	51447163	51447222	0	25
chr10	A_53_P120731	51447163	51447222	0	25
chr10	A_53_P120731	51447163	51447222	0	25
chr10	A_53_P179423	74979039	74979095	0	25
chr10	A_53_P104449	78690108	78690167	25	0
chr10	A_53_P171067	78712875	78712934	25	0
chr10	A_53_P101125	79386680	79386732	25	0
chr10	A_67_P07121650	79551705	79551759	25	0
chr10	A_67_P02168468	79560111	79560170	25	0
chr10	A_53_P110235	80029727	80029786	25	0
chr10	A_53_P103331	80737944	80737990	25	0
chr10	A_53_P140786	80741755	80741812	25	0
chr10	A_53_P139712	80751620	80751677	25	0
chr10	A_67_P02170711	80758816	80758875	25	0
chr10	A_53_P160230	80896536	80896580	25	0
chr10	A_53_P158295	80905798	80905848	25	0
chr10	A_67_P02171033	80914889	80914948	25	0
chr10	A_53_P172610	80926954	80927005	25	0
chr10	A_67_P07180313	105155301	105155360	0	25
chr10	A_53_P102134	127134674	127134733	25	0
chr11	A_53_P154523	49929144	49929195	0	25
chr11	A_67_P02330042	54925251	54925308	0	25
chr11	A_53_P102218	54944222	54944281	0	25
chr11	A_53_P113782	54950125	54950184	0	25
chr11	A_53_P152998	60053092	60053137	0	25
chr11	A_53_P179821	60054094	60054147	0	25
chr11	A_53_P151013	60054326	60054371	0	25
chr11	A_53_P155742	60719571	60719630	0	25
chr11	A_53_P106635	60722980	60723036	0	25
chr11	A_53_P137263	60741985	60742044	0	25
chr11	A_53_P127978	60745553	60745612	0	25
chr11	A_53_P179944	77729608	77729666	25	0
chr11	A_53_P115779	94055296	94055355	25	0
chr11	A_53_P163046	94055785	94055840	25	0
chr11	A_53_P158758	94056647	94056699	25	0
chr11	A_67_P07447225	94073941	94074000	25	0
chr11	A_53_P158918	94489888	94489947	0	25
chr11	A_53_P125833	94829857	94829916	25	0
chr11	A_53_P145038	94838417	94838476	25	0
chr11	A_53_P139517	94842731	94842781	25	0
chr11	A_53_P120174	112503933	112503988	0	25
chr11	A_53_P140072	118149288	118149347	25	0
chr11	A_53_P105331	121532307	121532366	0	25
chr11	A_53_P105331	121532307	121532366	0	25

chr11	A_53_P105331	121532307	121532366	0	25
chr12	A_67_P07540943	12362435	12362494	25	0
chr12	A_67_P07583382	34526953	34527012	25	0
chr12	A_67_P07583401	34537196	34537255	25	0
chr12	A_53_P140161	45076245	45076304	25	0
chr12	A_67_P07604830	45086611	45086670	25	0
chr12	A_67_P07629427	57246480	57246539	0	25
chr12	A_67_P07663410	74001467	74001526	0	25
chr12	A_53_P153412	81886135	81886192	0	25
chr12	A_53_P122053	99200727	99200786	0	25
chr12	A_53_P101282	103801250	103801309	0	25
chr12	A_53_P178876	103807346	103807405	0	25
chr12	A_53_P166953	103807850	103807909	0	25
chr13	A_67_P07800274	18792185	18792244	0	50
chr13	A_67_P07810826	23885875	23885934	0	25
chr13	A_53_P154257	23895497	23895556	0	25
chr13	A_53_P154257	23895497	23895556	0	25
chr13	A_53_P154257	23895497	23895556	0	25
chr13	A_53_P173873	23902797	23902856	0	25
chr13	A_67_P07818090	27155183	27155242	25	0
chr13	A_67_P07818169	27210860	27210919	25	0
chr13	A_53_P170219	101714168	101714227	0	25
chr13	A_53_P178026	101842739	101842798	25	0
chr13	A_53_P120963	105288825	105288884	0	25
chr13	A_53_P146207	105295215	105295274	0	25
chr13	A_53_P168106	105299626	105299685	0	25
chr13	A_53_P163495	106134302	106134361	0	25
chr13	A_53_P163495	106134302	106134361	0	25
chr13	A_53_P163495	106134302	106134361	0	25
chr14	A_67_P08046141	19320156	19320215	25	0
chr14	A_67_P02806702	19808158	19808216	0	25
chr14	A_53_P164346	46302419	46302478	0	25
chr14	A_53_P150330	46316577	46316636	0	25
chr14	A_67_P09541093	51406262	51406321	25	0
chr14	A_67_P09541099	51409109	51409168	25	0
chr14	A_53_P139209	51411970	51412025	25	0
chr14	A_67_P09541122	51417164	51417223	25	0
chr14	A_67_P08122673	56086711	56086770	25	0
chr14	A_67_P02856200	56097748	56097807	25	0
chr14	A_53_P164866	74062544	74062603	25	0
chr14	A_53_P151273	74065315	74065374	25	0
chr14	A_67_P02887043	74086065	74086124	25	0
chr14	A_67_P08202280	92835033	92835092	25	0
chr14	A_67_P08202299	92845694	92845753	25	0
chr14	A_67_P08202312	92850513	92850572	25	0
chr14	A_67_P08202326	92855795	92855854	25	0
chr14	A_67_P08209779	96821274	96821333	0	25
chr14	A_53_P115306	96836786	96836845	0	25
chr14	A_53_P115306	96836786	96836845	0	25
chr14	A_53_P115306	96836786	96836845	0	25
chr14	A_53_P145693	96836966	96837025	0	25
chr14	A_53_P145898	96837174	96837233	0	25
chr14	A_67_P08209849	96850408	96850467	0	25
chr15	A_67_P08315264	26758170	26758229	25	0
chr15	A_67_P08341576	38867642	38867701	0	25
chr15	A_67_P08341576	38867642	38867701	0	25
chr15	A_67_P08341576	38867642	38867701	0	25
chr15	A_67_P08394141	63940802	63940861	25	0
chr15	A_53_P166918	88970933	88970988	25	0
chr15	A_53_P171948	88975221	88975280	25	0
chr15	A_53_P126649	88976584	88976635	25	0
chr15	A_53_P127105	88983305	88983364	25	0
chr15	A_53_P136286	88995066	88995122	25	0
chr15	A_53_P173334	98421490	98421546	25	0
chr16	A_67_P03139062	16574551	16574610	25	0
chr16	A_53_P139354	32162433	32162492	25	0
chr16	A_53_P130295	32170718	32170777	25	0
chr16	A_67_P08554044	32179183	32179242	25	0
chr16	A_53_P108271	33690130	33690189	25	0
chr16	A_53_P108271	33690130	33690189	25	0
chr16	A_53_P108271	33690130	33690189	25	0
chr16	A_67_P08557914	33708617	33708676	25	0
chr17	A_53_P133081	25809378	25809437	25	0
chr17	A_53_P150583	27895538	27895594	0	25
chr17	A_53_P152162	27902578	27902637	0	25
chr17	A_53_P171490	27905939	27905998	0	25
chr17	A_53_P115225	34684977	34685024	25	0
chr17	A_53_P106959	34685847	34685892	25	0
chr17	A_53_P138468	34686683	34686735	25	0
chr17	A_53_P137281	34702345	34702391	25	0
chr17	A_53_P116826	36585761	36585820	0	25
chr17	A_53_P115438	36594311	36594370	0	25
chr17	A_53_P158116	46124596	46124642	25	0
chr17	A_53_P173922	52980852	52980911	0	25
chr17	A_53_P116883	52989730	52989789	0	25
chr17	A_53_P116883	52989730	52989789	0	25
chr17	A_53_P116883	52989730	52989789	0	25
chr17	A_53_P152639	53000512	53000571	0	25
chr17	A_53_P152639	53000512	53000571	0	25
chr17	A_53_P152639	53000512	53000571	0	25
chr17	A_53_P118521	53007268	53007327	0	25
chr17	A_67_P08810155	55605452	55605511	25	0

chr17	A_53_P130606	55696986	55697035	25	0
chr17	A_53_P113362	74209351	74209410	25	0
chr18	A_67_P03431087	24557959	24558018	25	0
chr18	A_67_P08981643	43426689	43426748	25	0
chr18	A_53_P133748	43440380	43440439	25	0
chr18	A_67_P08982703	43844696	43844755	25	0
chr18	A_53_P104760	43854543	43854602	25	0
chr18	A_53_P131268	43859666	43859725	25	0
chr18	A_53_P131268	43859666	43859725	25	0
chr18	A_53_P131268	43859666	43859725	25	0
chr18	A_53_P147446	43861060	43861119	25	0
chr18	A_67_P08982782	43876946	43877005	25	0
chr18	A_53_P113407	43892688	43892747	25	0
chr18	A_67_P08989484	47030213	47030272	25	0
chr18	A_67_P03476429	52727853	52727912	25	0
chr18	A_53_P146645	52741567	52741626	25	0
chr18	A_53_P147369	52745782	52745841	25	0
chr18	A_53_P117213	53540243	53540302	25	0
chr18	A_67_P09003475	53550724	53550780	25	0
chr18	A_67_P09003505	53562019	53562078	25	0
chr18	A_67_P09003505	53562019	53562078	25	0
chr18	A_67_P09003505	53562019	53562078	25	0
chr18	A_53_P118567	53577601	53577660	25	0
chr18	A_67_P09040679	69301554	69301613	25	0
chr18	A_53_P153176	69321992	69322048	25	0
chr18	A_53_P136300	69324270	69324314	50	0
chr18	A_53_P136300	69324270	69324314	50	0
chr18	A_53_P136300	69324270	69324314	50	0
chr18	A_53_P135024	69324293	69324352	50	0
chr18	A_53_P108782	69324323	69324378	50	0
chr18	A_53_P165013	69325674	69325733	50	0
chr18	A_67_P09040796	69347825	69347884	50	0
chr18	A_67_P09045731	71525194	71525253	25	0
chr18	A_53_P175080	71542028	71542087	25	0
chr18	A_53_P157166	71543199	71543258	25	0
chr19	A_53_P110968	5366093	5366140	25	0
chr19	A_67_P09100045	8640093	8640152	50	0
chr19	A_53_P178543	12528984	12529040	25	0
chr19	A_53_P170355	23991393	23991452	0	25
chr19	A_53_P137572	32858532	32858591	0	25
chr19	A_53_P137572	32858532	32858591	0	25
chr19	A_53_P137572	32858532	32858591	0	25
chr19	A_53_P165169	42655157	42655216	25	0
chr19	A_53_P154274	42658537	42658586	25	0
chr19	A_67_P09176752	42671023	42671082	25	0
chr19	A_67_P03601847	43700489	43700548	25	0
chrX	A_53_P148429	6761992	6762051	25	0
chrX	A_53_P121696	6771589	6771648	25	0
chrX	A_53_P133445	52942175	52942234	25	0
chrX	A_53_P111467	57434621	57434680	25	0
chrX	A_53_P167072	70450454	70450513	25	0
chrX	A_53_P149742	70459045	70459104	25	0
chrX	A_53_P149742	70459045	70459104	25	0
chrX	A_53_P149742	70459045	70459104	25	0
chrX	A_53_P134109	71665920	71665979	25	0
chrX	A_53_P122398	71671541	71671600	25	0
chrX	A_53_P172749	71690706	71690765	25	0
chrX	A_53_P160488	97934778	97934826	25	0
chrX	A_53_P126106	97935706	97935751	25	0
chrX	A_67_P09376608	97949869	97949928	25	0
chrX	A_67_P09434157	133885375	133885434	25	0
chrX	A_67_P09434207	133910324	133910383	25	0
chrX	A_67_P09465815	150587326	150587385	0	25
chrX	A_53_P151451	150606521	150606580	0	25
chrX	A_53_P159083	150608400	150608459	0	25
chrX	A_53_P159083	150608400	150608459	0	25
chrX	A_53_P159083	150608400	150608459	0	25
chrX	A_67_P09465901	150620880	150620939	0	25
chrY	A_67_P09495664	32258	32317	0	25
chrY	A_67_P09495665	37029	37088	0	25
chrY	A_67_P09495666	37653	37712	0	25
chrY	A_67_P09495672	47507	47560	0	25
chrY	A_67_P09495676	49697	49756	0	25
chrY	A_67_P09495677	56946	57005	0	25
chrY	A_67_P09495679	57466	57525	0	25
chrY	A_67_P09495681	58972	59027	0	25
chrY	A_67_P09495683	59668	59727	0	25
chrY	A_67_P09495685	60221	60280	0	25
chrY	A_67_P09495687	60984	61043	0	25
chrY	A_67_P09495688	61333	61392	0	25
chrY	A_67_P09495689	61790	61849	0	25
chrY	A_67_P09495692	62775	62834	0	25
chrY	A_67_P09495695	63474	63533	0	25
chrY	A_67_P09495696	63878	63937	0	25
chrY	A_67_P09495698	64309	64368	0	25
chrY	A_67_P09495702	66346	66405	0	25
chrY	A_67_P09496642	623572	623631	0	25
chrY	A_67_P09496643	623885	623944	0	25
chrY	A_67_P09496644	627345	627404	0	50
chrY	A_67_P09496646	628070	628121	0	75
chrY	A_67_P09496649	628670	628729	0	75
chrY	A_67_P09496652	629548	629607	0	75

chrY	A_67_P09496654	630269	630328	0	75
chrY	A_67_P09496657	631098	631157	0	75
chrY	A_67_P09496658	631698	631757	0	75
chrY	A_67_P09496659	631964	632023	0	75
chrY	A_67_P09496660	632329	632388	0	75
chrY	A_67_P09496661	632616	632675	0	75
chrY	A_67_P09496665	634102	634161	0	75
chrY	A_67_P09496668	635448	635507	0	75
chrY	A_67_P09496670	635897	635956	0	50
chrY	A_67_P09496672	636208	636267	0	50
chrY	A_67_P09496673	637137	637196	0	50
chrY	A_67_P09496674	637413	637471	0	50
chrY	A_67_P09496676	638747	638806	0	50
chrY	A_67_P09496678	639320	639379	0	50
chrY	A_67_P09496679	639815	639874	0	75
chrY	A_67_P09496682	640215	640274	0	75
chrY	A_67_P09496683	640588	640647	0	75
chrY	A_67_P09496685	641014	641073	0	75
chrY	A_67_P09496688	641515	641574	0	75
chrY	A_67_P09496690	641899	641958	0	75
chrY	A_67_P09496691	642598	642657	0	50
chrY	A_67_P09496693	642954	643013	0	50
chrY	A_67_P09496694	643417	643476	0	75
chrY	A_67_P09496695	643727	643786	0	75
chrY	A_67_P09496696	644057	644116	0	75
chrY	A_67_P09496700	644615	644674	0	75
chrY	A_67_P09496703	645418	645467	0	75
chrY	A_67_P09496704	646853	646912	0	50
chrY	A_67_P09496706	647133	647192	0	50
chrY	A_67_P09496707	647494	647553	0	50
chrY	A_67_P09496708	647770	647829	0	75
chrY	A_67_P09496709	648169	648228	0	75
chrY	A_67_P09496711	649317	649376	0	50
chrY	A_67_P09496714	650222	650281	0	75
chrY	A_67_P09496716	650704	650763	0	75
chrY	A_67_P09496719	651036	651095	0	75
chrY	A_67_P09496720	651315	651374	0	75
chrY	A_67_P09496721	651654	651713	0	75
chrY	A_67_P09496722	652080	652139	0	75
chrY	A_67_P09496724	652377	652436	0	75
chrY	A_67_P09496725	652730	652789	0	75
chrY	A_67_P09496726	653014	653070	0	75
chrY	A_67_P09496728	653581	653640	0	75
chrY	A_67_P09496729	653867	653926	0	75
chrY	A_67_P09496730	654154	654213	0	75
chrY	A_67_P09496731	654557	654616	0	75
chrY	A_67_P09496732	654904	654963	0	75
chrY	A_67_P09496733	655465	655524	0	75
chrY	A_67_P09496738	660856	660915	0	75
chrY	A_67_P09496739	661121	661180	0	75
chrY	A_67_P09496741	661473	661532	0	75
chrY	A_67_P09496742	662598	662657	0	75
chrY	A_67_P09496743	663678	663737	0	75
chrY	A_67_P09496744	664623	664682	0	75
chrY	A_67_P09496746	665310	665369	0	75
chrY	A_67_P09496747	666541	666600	0	75
chrY	A_67_P09496750	667350	667409	0	75
chrY	A_67_P09496752	667986	668045	0	75
chrY	A_67_P09496753	668539	668598	0	75
chrY	A_67_P09496755	669104	669163	0	75
chrY	A_67_P09496756	669418	669477	0	75
chrY	A_67_P09496757	670009	670068	0	75
chrY	A_67_P09496760	670297	670356	0	75
chrY	A_67_P09496761	670772	670831	0	75
chrY	A_67_P09496763	671329	671388	0	75
chrY	A_67_P09496764	671623	671682	0	75
chrY	A_67_P09496765	672167	672226	0	75
chrY	A_67_P09496768	672730	672789	0	75
chrY	A_67_P09496770	673484	673543	0	75
chrY	A_67_P09496771	674648	674707	0	75
chrY	A_67_P09496772	675552	675611	0	50
chrY	A_67_P09496773	676399	676458	0	50
chrY	A_67_P09496777	677292	677351	0	50
chrY	A_67_P09496779	677920	677979	0	50
chrY	A_67_P09496780	678180	678239	0	50
chrY	A_67_P09496783	679819	679878	0	25
chrY	A_67_P09496784	680224	680283	0	25
chrY	A_67_P09496785	680741	680800	0	50
chrY	A_67_P09496788	681659	681718	0	50
chrY	A_67_P09496792	687024	687083	0	50
chrY	A_67_P09496793	687335	687394	0	50
chrY	A_67_P09496795	687961	688020	0	50
chrY	A_67_P09496796	688281	688340	0	50
chrY	A_67_P09496799	688605	688664	0	50
chrY	A_67_P09496800	689126	689185	0	50
chrY	A_67_P09496803	690353	690412	0	50
chrY	A_67_P09496804	690811	690870	0	50
chrY	A_67_P09496805	691106	691165	0	50
chrY	A_67_P09496806	691676	691735	0	75
chrY	A_67_P09496808	692380	692439	0	50
chrY	A_67_P09496811	692699	692758	0	50
chrY	A_67_P09496813	693327	693386	0	50

chrY	A_67_P09496814	693733	693792	0	50
chrY	A_67_P09496815	697057	697116	0	75
chrY	A_67_P09496816	697432	697491	0	75
chrY	A_67_P09496817	699473	699517	0	75
chrY	A_67_P09496819	699765	699813	0	75
chrY	A_67_P09496821	701032	701091	0	75
chrY	A_67_P09496825	701902	701961	0	50
chrY	A_67_P09496827	702769	702828	0	50
chrY	A_67_P09496828	703225	703284	0	50
chrY	A_67_P09496830	703517	703575	0	50
chrY	A_67_P09496832	703962	704021	0	50
chrY	A_67_P09496833	704396	704455	0	50
chrY	A_67_P09496836	704729	704788	0	50
chrY	A_67_P09496837	705004	705058	0	50
chrY	A_67_P09496838	705993	706052	0	75
chrY	A_67_P09496840	717240	717299	0	50
chrY	A_67_P09496841	717601	717660	0	50
chrY	A_67_P09496844	718293	718352	0	50
chrY	A_67_P09496845	719247	719306	0	50
chrY	A_67_P09496847	719921	719980	0	50
chrY	A_67_P09496849	720549	720608	0	50
chrY	A_67_P09496854	721204	721263	0	50
chrY	A_67_P09496855	721660	721719	0	50
chrY	A_67_P09496885	723246	723305	0	100
chrY	A_67_P09496884	723606	723665	0	75
chrY	A_67_P09496882	724214	724273	0	75
chrY	A_67_P09496881	726601	726660	0	50
chrY	A_67_P09496880	727217	727276	0	50
chrY	A_67_P09496876	728034	728093	0	50
chrY	A_67_P09496875	728490	728549	0	50
chrY	A_67_P09496874	728876	728935	0	50
chrY	A_67_P09496873	729306	729365	0	50
chrY	A_67_P09496871	729900	729959	0	25
chrY	A_67_P09496870	730285	730344	0	50
chrY	A_67_P09496869	730810	730869	0	50
chrY	A_67_P09496868	731246	731305	0	25
chrY	A_67_P09496867	731532	731591	0	25
chrY	A_67_P09496867	731532	731591	0	25
chrY	A_67_P09496867	731532	731591	0	25
chrY	A_67_P09496866	731856	731915	0	25
chrY	A_67_P09496864	732462	732521	0	25
chrY	A_67_P09496862	733114	733173	0	25
chrY	A_67_P09496861	733675	733734	0	25
chrY	A_67_P09496888	743324	743383	0	50
chrY	A_67_P09496891	746327	746386	0	50
chrY	A_67_P09496892	746894	746953	0	50
chrY	A_67_P09496893	747278	747337	0	50
chrY	A_67_P09496895	747897	747956	0	50
chrY	A_67_P09496896	748399	748458	0	75
chrY	A_67_P09496897	748820	748879	0	75
chrY	A_67_P09496901	764310	764369	0	50
chrY	A_67_P09496902	764658	764717	0	50
chrY	A_67_P09496905	765433	765484	0	75
chrY	A_67_P09496906	765932	765991	0	75
chrY	A_67_P09496909	767056	767115	0	50
chrY	A_67_P09496910	767919	767978	0	100
chrY	A_67_P09496911	768258	768317	0	100
chrY	A_67_P09496913	768750	768809	0	100
chrY	A_67_P09496915	769887	769946	0	100
chrY	A_67_P09496916	770276	770335	0	100
chrY	A_67_P09496917	770560	770619	0	100
chrY	A_67_P09496918	770840	770899	0	100
chrY	A_67_P09496921	771509	771568	0	50
chrY	A_67_P09496922	771811	771870	0	100
chrY	A_67_P09496924	774972	775031	0	50
chrY	A_67_P09496925	775407	775466	0	50
chrY	A_67_P09496926	775742	775801	0	75
chrY	A_67_P09496928	776520	776579	0	50
chrY	A_67_P09496929	776898	776957	0	75
chrY	A_67_P09496930	777197	777256	0	75
chrY	A_67_P09496931	777594	777653	0	75
chrY	A_67_P09496932	777990	778049	0	75
chrY	A_67_P09496933	778485	778544	0	75
chrY	A_67_P09496935	779015	779074	0	75
chrY	A_67_P09496937	779518	779577	0	75
chrY	A_67_P09496938	780071	780130	0	75
chrY	A_67_P09496942	780989	781048	0	75
chrY	A_67_P09496943	781759	781818	0	75
chrY	A_67_P09496944	782048	782107	0	75
chrY	A_67_P09496946	782652	782711	0	75
chrY	A_67_P09496947	783230	783289	0	75
chrY	A_67_P09496949	783584	783643	0	75
chrY	A_67_P09496950	784163	784222	0	75
chrY	A_67_P09496950	784163	784222	0	75
chrY	A_67_P09496950	784163	784222	0	75
chrY	A_67_P09496951	784675	784734	0	75
chrY	A_67_P09496954	785451	785510	0	75
chrY	A_67_P09496955	786004	786063	0	75
chrY	A_67_P09496956	786266	786325	0	75
chrY	A_67_P09496957	786751	786810	0	75
chrY	A_67_P09496958	787301	787360	0	75
chrY	A_67_P09496959	787604	787663	0	75

chrY	A_67_P09496960	788271	788330	0	75
chrY	A_67_P09496961	788604	788663	0	75
chrY	A_67_P09496962	788991	789050	0	75
chrY	A_67_P09496964	790418	790477	0	50
chrY	A_67_P09496965	790754	790813	0	75
chrY	A_67_P09496967	791059	791118	0	50
chrY	A_67_P09496969	791570	791629	0	50
chrY	A_67_P09496970	792056	792115	0	50
chrY	A_67_P09496971	792392	792451	0	50
chrY	A_67_P09496972	793534	793593	0	75
chrY	A_67_P09496974	794678	794737	0	75
chrY	A_67_P09496976	795254	795313	0	50
chrY	A_67_P09496977	795793	795845	0	50
chrY	A_67_P09496979	796130	796189	0	50
chrY	A_67_P09496981	796753	796812	0	50
chrY	A_67_P09496982	798108	798167	0	50
chrY	A_67_P09496983	798920	798974	0	50
chrY	A_67_P09496984	799215	799274	0	50
chrY	A_67_P09496985	799656	799715	0	50
chrY	A_67_P09496987	800431	800490	0	50
chrY	A_67_P09496988	800792	800851	0	50
chrY	A_67_P09496989	801169	801228	0	50
chrY	A_67_P09496990	801874	801933	0	50
chrY	A_67_P09497449	1602758	1602817	0	25
chrY	A_67_P09497453	1604555	1604614	0	25
chrY	A_67_P09497459	1607051	1607110	0	25
chrY	A_67_P09497467	1609749	1609800	0	25
chrY	A_67_P09497471	1611237	1611288	0	25
chrY	A_67_P09497483	1620306	1620365	0	25
chrY	A_67_P09497492	1623398	1623449	0	25
chrY	A_67_P09497497	1625424	1625483	0	25
chrY	A_67_P09497502	1627320	1627379	0	25
chrY	A_67_P09497528	1641299	1641358	0	25
chrY	A_67_P09497556	1667176	1667235	0	25
chrY	A_67_P09497564	1669747	1669806	0	25
chrY	A_67_P09497565	1670249	1670308	0	25
chrY	A_67_P09497570	1671998	1672057	0	25
chrY	A_67_P09497573	1672749	1672808	0	25
chrY	A_67_P09497582	1677061	1677120	0	25
chrY	A_67_P09497601	1684098	1684157	0	25
chrY	A_67_P09497603	1684861	1684920	0	25
chrY	A_67_P09497606	1685953	1686012	0	25
chrY	A_67_P09497612	1699586	1699645	0	50
chrY	A_67_P09497615	1700636	1700695	0	50
chrY	A_67_P09497618	1702869	1702923	0	25
chrY	A_67_P09497620	1703624	1703673	0	50
chrY	A_67_P09497630	1708607	1708666	0	25
chrY	A_67_P09497631	1709117	1709167	0	25
chrY	A_67_P09497633	1709788	1709847	0	25
chrY	A_67_P09497635	1710446	1710498	0	25
chrY	A_67_P09497637	1711122	1711181	0	25
chrY	A_67_P09497643	1712381	1712435	0	25
chrY	A_67_P09497645	1712871	1712930	0	25
chrY	A_67_P09497647	1713604	1713661	0	25
chrY	A_67_P09497649	1714487	1714546	0	25
chrY	A_67_P09497671	1731943	1732001	0	25
chrY	A_67_P09497710	1756587	1756646	0	25
chrY	A_67_P09497721	1772303	1772362	0	25
chrY	A_67_P09497749	1788059	1788107	0	25
chrY	A_67_P09497751	1788886	1788945	0	25
chrY	A_67_P09497753	1789482	1789541	0	25
chrY	A_67_P09497762	1798851	1798910	0	50
chrY	A_67_P09497766	1800150	1800206	0	50
chrY	A_67_P09497768	1806959	1807018	0	25
chrY	A_67_P09497777	1813416	1813475	0	25
chrY	A_67_P09497778	1813881	1813940	0	25
chrY	A_67_P09497805	1823971	1824026	0	50
chrY	A_67_P09497818	1830151	1830210	0	50
chrY	A_67_P09497819	1830633	1830692	0	50
chrY	A_67_P09497821	1832953	1833012	0	50
chrY	A_67_P09497822	1834587	1834646	0	50
chrY	A_67_P09497824	1835307	1835366	0	50

The locations of copy number gain or loss observed from tumors in the mouse genome and the genes present in those regions
Deletion and amplification columns are percentage of tumors with a deletion or amplification.
