# Supplementary material for: Predisposition to Cancer Caused by Genetic and Functional Defects of Mammalian Atad5
Source: PLoS Genet. 2011 Aug 25;7(8):e1002245. doi: 10.1371/journal.pgen.1002245 (PMC3161924; doi:10.1371/journal.pgen.1002245)
Supplement: Table S2 — List of cancer gene transcripts that had up or down regulated expression in two tumor samples compared to their normal controls. (PDF) [file pgen.1002245.s006.pdf]

Table S2. List of cancer gene transcripts that had up or down regulated expressioin in two tumor samples compared to their normal controls

Notes : Fold change >= 1.0

ProbeName	Fold change([TUMOR] vs [COI	Regulation([TUMOR] vs [	Common name	GeneSymbol	GenbankAccession	Description
A_52_P430074	1.8096836	down	AK152535	Abcb7	AK152535	Mus musculus bone marrow macrophage cDNA, RIKEN full-length enriched library, clone:I830078I15 product:ATP-binding cassette, sub-family B (MDR/TAP), member 7, full insert sequence. [AK152535]
A_52_P360650	2.453843	down	XM_907304	Abcb7	XM_907304	PREDICTED: Mus musculus ATP-binding cassette, sub-family B (MDR/TAP), member 7, transcript variant 5 (Abcb7), mRNA [XM_907304]
A_51_P363556	4.2834387	up	NM_198127	Abi2	NM_198127	Mus musculus abl-interactor 2 (Abi2), mRNA [NM_198127]
A_52_P530042	4.517351	up	NM_198127	Abi2	NM_198127	Mus musculus abl-interactor 2 (Abi2), mRNA [NM_198127]
A_52_P141338	1.2804152	down	NM_022816	Acot10	NM_022816	Mus musculus acyl-CoA thioesterase 10 (Acot10), mRNA [NM_022816]
A_52_P392674	1.1627663	down	NM_019736	Acot9	NM_019736	Mus musculus acyl-CoA thioesterase 9 (Acot9), mRNA [NM_019736]
A_52_P664656	1.7672709	down	NM_007396	Acvr2a	NM_007396	Mus musculus activin receptor IIA (Acvr2a), mRNA [NM_007396]
A_52_P575854	2.2381537	down	NM_007396	Acvr2a	NM_007396	Mus musculus activin receptor IIA (Acvr2a), mRNA [NM_007396]
A_51_P148290	4.214034	up	NM_172875	Adc	NM_172875	Mus musculus arginine decarboxylase (Adc), mRNA [NM_172875]
A_51_P428555	7.6123295	down	NM_007409	Adh1	NM_007409	Mus musculus alcohol dehydrogenase 1 (class I) (Adh1), mRNA [NM_007409]
A_52_P629895	7.6195316	down	NM_007409	Adh1	NM_007409	Mus musculus alcohol dehydrogenase 1 (class I) (Adh1), mRNA [NM_007409]
A_51_P342567	6.9533815	up	NM_031185	Akap12	NM_031185	Mus musculus A kinase (PRKA) anchor protein (gravin) 12 (Akap12), mRNA [NM_031185]
A_51_P359272	9.003699	down	NM_009655	Alcam	NM_009655	Mus musculus activated leukocyte cell adhesion molecule (Alcam), mRNA [NM_009655]
A_51_P429681	1.7780472	up	AK033397	Apbb2	AK033397	Mus musculus 16 days embryo lung cDNA, RIKEN full-length enriched library, clone:8430429I17 product:unclassifiable, full insert sequence [AK033397]
A_51_P149562	1.9322677	up	NM_009686	Apbb2	NM_009686	Mus musculus amyloid beta (A4) precursor protein-binding, family B, member 2 (Apbb2), mRNA [NM_009686]
A_52_P555603	3.4982803	up	AK032770	Apbb2	AK032770	Mus musculus 12 days embryo male wolffian duct includes surrounding region cDNA, RIKEN full-length enriched library, clone:6720431O15 product:amyloid beta (A4) precursor protein-binding, family B, member 2, full insert sequence. [AK032770]
A_51_P223946	1.6330367	up	AK122414	Aprin	AK122414	Mus musculus mRNA for mKIAA0979 protein [AK122414]
A_52_P24783	2.1686547	up	NM_175310	Aprin	NM_175310	Mus musculus androgen-induced proliferation inhibitor (Aprin), mRNA [NM_175310]
A_52_P617762	2.0554051	down	AK039081	Arhgap5	AK039081	Mus musculus adult male hypothalamus cDNA, RIKEN full-length enriched library, clone:A230093J24 product:unclassifiable, full insert sequence. [AK039081]
A_52_P260864	2.4404852	down	NM_009706	Arhgap5	NM_009706	Mus musculus Rho GTPase activating protein 5 (Arhgap5), mRNA [NM_009706]
A_52_P551909	2.6211565	down	NM_009706	Arhgap5	NM_009706	Mus musculus Rho GTPase activating protein 5 (Arhgap5), mRNA [NM_009706]
A_52_P614762	1.5585276	down	AK029400	Bad	AK029400	Mus musculus 0 day neonate head cDNA, RIKEN full-length enriched library, clone:4833429M11 product:Bcl-associated death promoter, full insert sequence. [AK029400]
A_51_P419364	2.6857738	down	NM_007522	Bad	NM_007522	Mus musculus Bcl-associated death promoter (Bad), mRNA [NM_007522]
A_51_P106263	2.1901217	up	NM_133926	Camk1	NM_133926	Mus musculus calcium/calmodulin-dependent protein kinase I (Camk1), mRNA [NM_133926]
A_51_P106259	2.258415	up	NM_133926	Camk1	NM_133926	Mus musculus calcium/calmodulin-dependent protein kinase I (Camk1), mRNA [NM_133926]
A_51_P262766	3.800415	up	S78355	Ccnd1	S78355	Cyl-1=cyclin D1 [mice, BALB/c, brain, mRNA, 3737 nt]. [S78355]
A_51_P248666	3.0001664	up	NM_021893	Cd274	NM_021893	Mus musculus CD274 antigen (Cd274), mRNA [NM_021893]
A_51_P375146	2.9806201	down	NM_007643	Cd36	NM_007643	Mus musculus CD36 antigen (Cd36), mRNA [NM_007643]
A_51_P375138	3.2577126	down	L23108	Cd36	L23108	Mus musculus CD36 antigen mRNA, complete cds. [L23108]
A_52_P683146	1.129669	up	NM_009866	Cdh11	NM_009866	Mus musculus cadherin 11 (Cdh11), mRNA [NM_009866]
A_52_P437795	6.037858	up	NM_009866	Cdh11	NM_009866	Mus musculus cadherin 11 (Cdh11), mRNA [NM_009866]
A_51_P506204	7.4964786	up	NM_009866	Cdh11	NM_009866	Mus musculus cadherin 11 (Cdh11), mRNA [NM_009866]
A_51_P193563	1.2164589	down	NM_009874	Cdk7	NM_009874	Mus musculus cyclin-dependent kinase 7 (homolog of Xenopus MO15 cdk-activating kinase) (Cdk7), mRNA [NM_009874]
A_52_P258991	2.0637655	down	AK145759	Cdk7	AK145759	Mus musculus blastocyst blastocyst cDNA, RIKEN full-length enriched library, clone:I1C0038J11 product:cyclin-dependent kinase 7 (homolog of Xenopus MO15 cdk-activating kinase), full insert sequence [AK145759]
A_51_P513719	2.98359	up	NM_007671	Cdkn2c	NM_007671	Mus musculus cyclin-dependent kinase inhibitor 2C (p18, inhibits CDK4) (Cdkn2c), mRNA [NM_007671]
A_51_P513720	3.0137582	up	U19596	Cdkn2c	U19596	Mus musculus Cdk4 and Cdk6 inhibitor p18 protein mRNA, complete cds. [U19596]
A_51_P246653	2.4548311	up	NM_020008	Clec7a	NM_020008	Mus musculus C-type lectin domain family 7, member a (Clec7a), mRNA [NM_020008]
A_52_P216436	1.6547683	down	NM_031163	Col2a1	NM_031163	Mus musculus procollagen, type II, alpha 1 (Col2a1), mRNA [NM_031163]
A_51_P317141	6.2127233	up	NM_031163	Col2a1	NM_031163	Mus musculus procollagen, type II, alpha 1 (Col2a1), mRNA [NM_031163]
A_51_P493234	12.867895	up	NM_007752	Cp	NM_007752	Mus musculus ceruloplasmin (Cp), transcript variant 2, mRNA [NM_007752]
A_51_P111762	2.3400002	down	NM_011804	Creg1	NM_011804	Mus musculus cellular repressor of E1A-stimulated genes 1 (Creg1), mRNA [NM_011804]
A_52_P348709	2.454221	down	NM_011804	Creg1	NM_011804	Mus musculus cellular repressor of E1A-stimulated genes 1 (Creg1), mRNA [NM_011804]
A_52_P155708	3.8975482	down	NM_011804	Creg1	NM_011804	Mus musculus cellular repressor of E1A-stimulated genes 1 (Creg1), mRNA [NM_011804]
A_52_P542419	2.378108	up	NM_013767	Csnk1e	NM_013767	Mus musculus casein kinase 1, epsilon (Csnk1e), mRNA [NM_013767]
A_51_P429197	2.4807916	up	NM_013767	Csnk1e	NM_013767	Mus musculus casein kinase 1, epsilon (Csnk1e), mRNA [NM_013767]
A_51_P363187	23.046015	up	NM_008176	Cxcl1	NM_008176	Mus musculus chemokine (C-X-C motif) ligand 1 (Cxcl1), mRNA [NM_008176]
A_51_P252859	8.381687	up	NM_010516	Cyr61	NM_010516	Mus musculus cysteine rich protein 61 (Cyr61), mRNA [NM_010516]
A_51_P123895	6.5943394	up	NM_053078	D0H4S114	NM_053078	Mus musculus DNA segment, human D4S114 (D0H4S114), mRNA [NM_053078]
A_52_P301821	6.656651	up	NM_053078	D0H4S114	NM_053078	Mus musculus DNA segment, human D4S114 (D0H4S114), mRNA [NM_053078]
A_51_P120636	3.1766858	up	NM_007891	E2f1	NM_007891	Mus musculus E2F transcription factor 1 (E2f1), mRNA [NM_007891]
A_52_P587611	3.7846649	up	NM_007891	E2f1	NM_007891	Mus musculus E2F transcription factor 1 (E2f1), mRNA [NM_007891]
A_51_P499876	2.5193021	up	L20334	Edg5	L20334	Mouse EDG-like receptor mRNA, partial cds. [L20334]
A_51_P499871	6.7542095	up	NM_010333	Edg5	NM_010333	Mus musculus endothelial differentiation, sphingolipid G-protein-coupled receptor, 5 (Edg5), mRNA [NM_010333]
A_52_P604613	6.815614	up	NM_010333	Edg5	NM_010333	Mus musculus endothelial differentiation, sphingolipid G-protein-coupled receptor, 5 (Edg5), mRNA [NM_010333]
A_52_P326354	3.1786594	up	NM_010155	Erf	NM_010155	Mus musculus Ets2 repressor factor (Erf), mRNA [NM_010155]
A_51_P224192	2.3398597	down	NM_011935	Esrrg	NM_011935	Mus musculus estrogen-related receptor gamma (Esrrg), mRNA [NM_011935]
A_52_P317246	76.70423	down	NM_011935	Esrrg	NM_011935	Mus musculus estrogen-related receptor gamma (Esrrg), mRNA [NM_011935]
A_52_P194250	7.0206957	up	NM_011808	Ets1	NM_011808	Mus musculus E26 avian leukemia oncogene 1, 5' domain (Ets1), transcript variant 1, mRNA [NM_011808]
A_52_P232830	7.1455355	up	NM_011808	Ets1	NM_011808	Mus musculus E26 avian leukemia oncogene 1, 5' domain (Ets1), transcript variant 1, mRNA [NM_011808]
A_51_P329043	7.3364906	up	X55787	Ets1	X55787	M.musculus c-ets-1 gene. [X55787]
A_52_P258291	9.294166	up	NM_011808	Ets1	NM_011808	Mus musculus E26 avian leukemia oncogene 1, 5' domain (Ets1), transcript variant 1, mRNA [NM_011808]
A_51_P480311	23.250677	down	NM_010168	F2	NM_010168	Mus musculus coagulation factor II (F2), mRNA [NM_010168]
A_52_P344290	4.809881	up	NM_010169	F2r	NM_010169	Mus musculus coagulation factor II (thrombin) receptor (F2r), mRNA [NM_010169]
A_52_P278336	3.5344594	up	NM_011812	Fbln5	NM_011812	Mus musculus fibulin 5 (Fbln5), mRNA [NM_011812]
A_52_P288050	5.8763804	up	AK084895	Fbln5	AK084895	Mus musculus 13 days embryo lung cDNA, RIKEN full-length enriched library, clone:D430006M14 product:fibulin 5, full insert sequence. [AK084895]
A_51_P282179	2.6078815	down	NM_020009	Frap1	NM_020009	Mus musculus FK506 binding protein 12-rapamycin associated protein 1 (Frap1), mRNA [NM_020009]
A_52_P67643	3.452959	down	BC043920	Frap1	BC043920	Mus musculus FK506 binding protein 12-rapamycin associated protein 1, mRNA (cDNA clone IMAGE:5064192), complete cds. [BC043920]
A_51_P441798	6.143435	down	X16314	Glul	X16314	M.musculus mRNA for glutamine synthetase. [X16314]
A_52_P101279	8.605247	down	NM_008131	Glul	NM_008131	Mus musculus glutamate-ammonia ligase (glutamine synthetase) (Glul), mRNA [NM_008131]
A_52_P143866	9.359642	down	NM_008131	Glul	NM_008131	Mus musculus glutamate-ammonia ligase (glutamine synthetase) (Glul), mRNA [NM_008131]
A_51_P498004	2.1664174	down	NM_010301	Gna11	NM_010301	Mus musculus guanine nucleotide binding protein, alpha 11 (Gna11), mRNA [NM_010301]
A_52_P366932	2.2694962	down	NM_010301	Gna11	NM_010301	Mus musculus guanine nucleotide binding protein, alpha 11 (Gna11), mRNA [NM_010301]
A_51_P275623	4.711687	down	NM_015828	Gne	NM_015828	Mus musculus glucosamine (Gne), mRNA [NM_015828]
A_52_P37593	4.7183385	down	NM_015828	Gne	NM_015828	Mus musculus glucosamine (Gne), mRNA [NM_015828]
A_52_P188099	8.8575535	down	AK033507	Gne	AK033507	Mus musculus adult male colon cDNA, RIKEN full-length enriched library, clone:9030414E18 product:UDP-N-acetylglucosamine-2-epimerase [AK033507]
A_52_P45841	2.8249576	down	NM_028976	Gorasp1	NM_028976	Mus musculus golgi reassembly stacking protein 1 (Gorasp1), mRNA [NM_028976]
A_52_P68221	5.018613	up	NM_016886	Gria3	NM_016886	Mus musculus glutamate receptor, ionotropic, AMPA3 (alpha 3) (Gria3), mRNA [NM_016886]
A_52_P179309	5.3627505	up	NM_016886	Gria3	NM_016886	Mus musculus glutamate receptor, ionotropic, AMPA3 (alpha 3) (Gria3), mRNA [NM_016886]
A_52_P272364	6.9007053	up	AK161163	Gria3	AK161163	Mus musculus 0 day neonate head cDNA, RIKEN full-length enriched library, clone:4831401I16 product:glutamate receptor, ionotropic, AMPA3 (alpha 3), full insert sequence. [AK161163]
A_51_P112223	4.040848	down	NM_010357	Gsta4	NM_010357	Mus musculus glutathione S-transferase, alpha 4 (Gsta4), mRNA [NM_010357]
A_52_P466741	1.1672565	down	NM_011826	Hax1	NM_011826	Mus musculus HCLS1 associated X-1 (Hax1), mRNA [NM_011826]
A_51_P437697	1.3066484	down	NM_011826	Hax1	NM_011826	Mus musculus HCLS1 associated X-1 (Hax1), mRNA [NM_011826]
A_52_P572818	2.070577	down	AF023483	Hax1	AF023483	Mus musculus HS-1-associating protein HAX-1L (mHAX-1s) pseudogene mRNA, complete sequence. [AF023483]
A_52_P29583	1.191088	down	AK016742	Hipk2	AK016742	Mus musculus adult male testis cDNA, RIKEN full-length enriched library, clone:4933408M17 product:homeodomain interacting protein kinase 2, full insert sequence. [AK016742]
A_51_P318292	1.8156087	down	NM_010433	Hipk2	NM_010433	Mus musculus homeodomain interacting protein kinase 2 (Hipk2), mRNA [NM_010433]
A_52_P554845	1.8751788	down	NM_010433	Hipk2	NM_010433	Mus musculus homeodomain interacting protein kinase 2 (Hipk2), mRNA [NM_010433]
A_51_P125316	2.2076242	down	AK003718	Hipk2	AK003718	Mus musculus 18-day embryo whole body cDNA, RIKEN full-length enriched library, clone:1110014O20 product:unclassifiable, full insert sequence. [AK003718]
A_51_P517182	1.3813775	down	AK052657	Hrasls3	AK052657	Mus musculus 0 day neonate kidney cDNA, RIKEN full-length enriched library, clone:D630015C01 product:H-REV 107 PROTEIN homolog [Rattus norvegicus], full insert sequence. [AK052657]
A_52_P154800	3.2264626	down	NM_139269	Hrasls3	NM_139269	Mus musculus HRAS like suppressor 3 (Hrasls3), mRNA [NM_139269]
A_52_P90363	2.305422	down	NM_029803	Ifi27	NM_029803	Mus musculus interferon, alpha-inducible protein 27 (Ifi27), mRNA [NM_029803]
A_51_P358233	2.4438343	down	AK010014	Ifi27	AK010014	Mus musculus adult male tongue cDNA, RIKEN full-length enriched library, clone:2310061N23 product:similar to ALPHA-INTERFERON INDUCIBLE PROTEIN (FRAGMENT) [Mesocricetus auratus], full insert sequence. [AK010014]
A_52_P90364	7.517165	down	NM_029803	Ifi27	NM_029803	Mus musculus interferon, alpha-inducible protein 27 (Ifi27), mRNA [NM_029803]
A_51_P490305	5.0101123	down	NM_023065	Ifi30	NM_023065	Mus musculus interferon gamma inducible protein 30 (Ifi30), mRNA [NM_023065]
A_52_P15461	2.1339648	down	NM_008357	Il15	NM_008357	Mus musculus interleukin 15 (Il15), mRNA [NM_008357]
A_52_P383066	2.6090174	down	AB022307	Il15	AB022307	Mus musculus mRNA for shorter isoform of interleukin 15, partial cds. [AB022307]
A_51_P239750	15.527142	up	NM_008380	Inhba	NM_008380	Mus musculus inhibin beta-A (Inhba), mRNA [NM_008380]

A_52_P549279	1.7002345	down	NM_172944	Itgae	NM_172944	Mus musculus integrin, alpha E, epithelial-associated (Itgae), transcript variant 2, mRNA [NM_172944]
A_51_P352076	2.2181423	down	NM_008399	Itgae	NM_008399	Mus musculus integrin, alpha E, epithelial-associated (Itgae), transcript variant 1, mRNA [NM_008399]
A_51_P425680	3.261504	down	NM_019826	Ivd	NM_019826	Mus musculus isovaleryl coenzyme A dehydrogenase (Ivd), mRNA [NM_019826]
A_52_P550069	3.5622003	down	NM_019826	Ivd	NM_019826	Mus musculus isovaleryl coenzyme A dehydrogenase (Ivd), mRNA [NM_019826]
A_51_P139410	5.449637	up	NM_008478	L1cam	NM_008478	Mus musculus L1 cell adhesion molecule (L1cam), mRNA [NM_008478]
A_51_P465281	5.0744843	up	NM_008495	Lgals1	NM_008495	Mus musculus lectin, galactose binding, soluble 1 (Lgals1), mRNA [NM_008495]
A_52_P480351	8.826755	up	XM_985144	Loxl2	XM_985144	PREDICTED: Mus musculus lysyl oxidase-like 2 (Loxl2), mRNA [XM_985144]
A_51_P275949	9.040233	up	NM_033325	Loxl2	NM_033325	Mus musculus lysyl oxidase-like 2 (Loxl2), mRNA [NM_033325]
A_52_P189946	2.759351	up	NM_019919	Ltbp1	NM_019919	Mus musculus latent transforming growth factor beta binding protein 1 (Ltbp1), transcript variant 1, mRNA [NM_019919]
A_51_P281716	5.700854	up	NM_019919	Ltbp1	NM_019919	Mus musculus latent transforming growth factor beta binding protein 1 (Ltbp1), transcript variant 1, mRNA [NM_019919]
A_51_P257457	3.3849494	up	NM_013589	Ltbp2	NM_013589	Mus musculus latent transforming growth factor beta binding protein 2 (Ltbp2), mRNA [NM_013589]
A_51_P425632	2.5662844	down	NM_025649	Mad2l1bp	NM_025649	Mus musculus MAD2L1 binding protein (Mad2l1bp), mRNA [NM_025649]
A_51_P426270	6.409573	up	NM_008597	Mgp	NM_008597	Mus musculus matrix Gla protein (Mgp), mRNA [NM_008597]
A_52_P415168	30.265642	down	NM_019394	Mia1	NM_019394	Mus musculus melanoma inhibitory activity 1 (Mia1), mRNA [NM_019394]
A_52_P415167	31.490252	down	NM_019394	Mia1	NM_019394	Mus musculus melanoma inhibitory activity 1 (Mia1), mRNA [NM_019394]
A_52_P174346	31.516293	down	NM_019394	Mia1	NM_019394	Mus musculus melanoma inhibitory activity 1 (Mia1), mRNA [NM_019394]
A_51_P216303	6.9670506	up	NM_008608	Mmp14	NM_008608	Mus musculus matrix metallopeptidase 14 (membrane-inserted) (Mmp14), mRNA [NM_008608]
A_52_P304128	9.432858	up	NM_008608	Mmp14	NM_008608	Mus musculus matrix metallopeptidase 14 (membrane-inserted) (Mmp14), mRNA [NM_008608]
A_52_P127130	8.683262	up	NM_031195	Msr1	NM_031195	Mus musculus macrophage scavenger receptor 1 (Msr1), mRNA [NM_031195]
A_51_P404846	9.584209	up	NM_031195	Msr1	NM_031195	Mus musculus macrophage scavenger receptor 1 (Msr1), mRNA [NM_031195]
A_52_P607128	9.630034	up	NM_031195	Msr1	NM_031195	Mus musculus macrophage scavenger receptor 1 (Msr1), mRNA [NM_031195]
A_52_P320971	2.5988462	down	NM_013603	Mt3	NM_013603	Mus musculus metallothionein 3 (Mt3), mRNA [NM_013603]
A_51_P309328	2.1514068	down	NM_013752	Nbn	NM_013752	Mus musculus nibrin (Nbn), mRNA [NM_013752]
A_52_P298548	2.3453636	down	NM_013752	Nbn	NM_013752	Mus musculus nibrin (Nbn), mRNA [NM_013752]
A_52_P860172	2.9339912	down	AK085134	Nbn	AK085134	Mus musculus 13 days embryo lung cDNA, RIKEN full-length enriched library, clone:D430042D16 product:unclassifiable, full insert sequence. [AK085134]
A_52_P907073	2.0523741	down	AK019159	Neo1	AK019159	Mus musculus 10 days embryo whole body cDNA, RIKEN full-length enriched library, clone:2610028H22 product:unclassifiable, full insert sequence. [AK019159]
A_51_P476728	2.1847363	down	NM_008684	Neo1	NM_008684	Mus musculus neogenin (Neo1), transcript variant 1, mRNA [NM_008684]
A_52_P269003	2.804242	down	NM_008684	Neo1	NM_008684	Mus musculus neogenin (Neo1), transcript variant 1, mRNA [NM_008684]
A_51_P452323	7.772345	up	NM_016701	Nes	NM_016701	Mus musculus nestin (Nes), mRNA [NM_016701]
A_51_P460143	10.083625	down	NM_015750	Neu2	NM_015750	Mus musculus neuraminidase 2 (Neu2), mRNA [NM_015750]
A_52_P183088	2.1586757	up	NM_008714	Notch1	NM_008714	Mus musculus Notch gene homolog 1 (Drosophila) (Notch1), mRNA [NM_008714]
A_51_P508510	2.2233179	up	Z11886	Notch1	Z11886	M.musculus notch-1 mRNA. [Z11886]
A_52_P225912	3.6903982	up	NM_130866	Olfr78	NM_130866	Mus musculus olfactory receptor 78 (Olfr78), mRNA [NM_130866]
A_52_P495729	2.6905644	up	NM_011019	Osmr	NM_011019	Mus musculus oncostatin M receptor (Osmr), mRNA [NM_011019]
A_51_P319460	2.7220554	up	NM_011019	Osmr	NM_011019	Mus musculus oncostatin M receptor (Osmr), mRNA [NM_011019]
A_52_P370274	7.3907814	up	AK171700	Pde4b	AK171700	Mus musculus activated spleen cDNA, RIKEN full-length enriched library, clone:F830005J09 product:phosphodiesterase 4B, cAMP specific, full insert sequence. [AK171700]
A_52_P423247	8.59967	up	NM_019840	Pde4b	NM_019840	Mus musculus phosphodiesterase 4B, cAMP specific (Pde4b), mRNA [NM_019840]
A_51_P512340	9.169716	up	NM_019840	Pde4b	NM_019840	Mus musculus phosphodiesterase 4B, cAMP specific (Pde4b), mRNA [NM_019840]
A_51_P224311	5.4113717	down	NM_022318	Popdc2	NM_022318	Mus musculus popeye domain containing 2 (Popdc2), transcript variant 2, mRNA [NM_022318]
A_52_P27332	1.1177047	down	NM_012023	Ppp2r5c	NM_012023	Mus musculus protein phosphatase 2, regulatory subunit B (B56), gamma isoform (Ppp2r5c), transcript variant 1, mRNA [NM_012023]
A_51_P320523	2.1761737	down	NM_012023	Ppp2r5c	NM_012023	Mus musculus protein phosphatase 2, regulatory subunit B (B56), gamma isoform (Ppp2r5c), transcript variant 1, mRNA [NM_012023]
A_52_P166952	2.2764115	down	NM_001081458	Ppp2r5c	NM_001081458	Mus musculus protein phosphatase 2, regulatory subunit B (B56), gamma isoform (Ppp2r5c), transcript variant 3, mRNA [NM_001081458]
A_52_P94495	1.2268181	up	AK050059	Prkcn	AK050059	Mus musculus adult male liver tumor cDNA, RIKEN full-length enriched library, clone:C730009D23 product:weakly similar to PROTEIN KINASE C BETA (FRAGMENT) [Homo sapiens], full insert sequence. [AK050059]
A_51_P413965	1.645991	up	DV056587	Prkcn	DV056587	DLP01_18_M21.x2 FH DLP01 Mus musculus cDNA clone DLP01_18_M21 similar to Protein kinase C nu, mRNA sequence [DV056587]
A_51_P106397	1.9773166	up	NM_029239	Prkcn	NM_029239	Mus musculus protein kinase C, nu (Prkcn), mRNA [NM_029239]
A_52_P669996	2.161055	up	NM_029239	Prkcn	NM_029239	Mus musculus protein kinase C, nu (Prkcn), mRNA [NM_029239]
A_52_P1012802	2.8035061	up	NM_008982	Ptprj	NM_008982	Mus musculus protein tyrosine phosphatase, receptor type, J (Ptprj), mRNA [NM_008982]
A_52_P1641	3.932017	up	NM_008982	Ptprj	NM_008982	Mus musculus protein tyrosine phosphatase, receptor type, J (Ptprj), mRNA [NM_008982]
A_52_P162697	1.1217824	up	NM_027514	Pvr	NM_027514	Mus musculus poliovirus receptor (Pvr), mRNA [NM_027514]
A_52_P162695	1.7960799	up	NM_027514	Pvr	NM_027514	Mus musculus poliovirus receptor (Pvr), mRNA [NM_027514]
A_52_P628590	1.9556673	up	NM_027514	Pvr	NM_027514	Mus musculus poliovirus receptor (Pvr), mRNA [NM_027514]
A_51_P237097	2.0245855	up	NM_027514	Pvr	NM_027514	Mus musculus poliovirus receptor (Pvr), mRNA [NM_027514]
A_52_P225377	4.047059	up	AK014446	Pvr	AK014446	Mus musculus 18 days pregnant adult female placenta and extra embryonic tissue cDNA, RIKEN full-length enriched library, clone:3830421F03 product:hypothetical protein, full insert sequence. [AK014446]
A_51_P241733	2.3632343	down	NM_145435	Pyy	NM_145435	Mus musculus peptide YY (Pyy), mRNA [NM_145435]
A_52_P366462	15.498475	down	NM_145435	Pyy	NM_145435	Mus musculus peptide YY (Pyy), mRNA [NM_145435]
A_51_P314763	1.4071349	down	NM_011231	Rabggtb	NM_011231	Mus musculus RAB geranylgeranyl transferase, b subunit (Rabggtb), mRNA [NM_011231]
A_52_P430628	1.5020984	down	NM_011231	Rabggtb	NM_011231	Mus musculus RAB geranylgeranyl transferase, b subunit (Rabggtb), mRNA [NM_011231]
A_52_P682873	2.1243684	down	BC057661	Rabggtb	BC057661	Mus musculus RAB geranylgeranyl transferase, b subunit, mRNA (cDNA clone IMAGE:3155581), complete cds. [BC057661]
A_52_P26409	2.35221	up	M32070	Rarg	M32070	Mouse retinoic acid receptor gamma 3 mRNA, 5' end. [M32070]
A_51_P372853	2.854912	up	NM_011244	Rarg	NM_011244	Mus musculus retinoic acid receptor, gamma (Rarg), transcript variant 1, mRNA [NM_011244]
A_51_P372858	3.0058188	up	NM_011244	Rarg	NM_011244	Mus musculus retinoic acid receptor, gamma (Rarg), transcript variant 1, mRNA [NM_011244]
A_52_P380418	3.537931	up	M32070	Rarg	M32070	Mouse retinoic acid receptor gamma 3 mRNA, 5' end. [M32070]
A_51_P271644	2.75481	down	NM_011246	Rasgrp1	NM_011246	Mus musculus RAS guanyl releasing protein 1 (Rasgrp1), mRNA [NM_011246]
A_52_P487303	8.492334	down	NM_011246	Rasgrp1	NM_011246	Mus musculus RAS guanyl releasing protein 1 (Rasgrp1), mRNA [NM_011246]
A_51_P224903	1.8568233	up	NM_009062	Rgs4	NM_009062	Mus musculus regulator of G-protein signaling 4 (Rgs4), mRNA [NM_009062]
A_52_P43326	2.3301473	up	NM_009062	Rgs4	NM_009062	Mus musculus regulator of G-protein signaling 4 (Rgs4), mRNA [NM_009062]
A_51_P314375	2.6451383	down	NM_080844	Serpinc1	NM_080844	Mus musculus serine (or cysteine) peptidase inhibitor, clade C (antithrombin), member 1 (Serpinc1), mRNA [NM_080844]
A_52_P409746	3.580984	down	NM_080844	Serpinc1	NM_080844	Mus musculus serine (or cysteine) peptidase inhibitor, clade C (antithrombin), member 1 (Serpinc1), mRNA [NM_080844]
A_51_P183571	15.332139	up	NM_008871	Serpine1	NM_008871	Mus musculus serine (or cysteine) peptidase inhibitor, clade E, member 1 (Serpine1), mRNA [NM_008871]
A_51_P145520	5.5402393	down	NM_023059	Sigirr	NM_023059	Mus musculus single immunoglobulin and toll-interleukin 1 receptor (TIR) domain (Sigirr), mRNA [NM_023059]
A_52_P640221	6.283906	down	NM_008767	Slc22a18	NM_008767	Mus musculus solute carrier family 22 (organic cation transporter), member 18 (Slc22a18), transcript variant 1, mRNA [NM_008767]
A_51_P165882	8.795703	down	NM_008767	Slc22a18	NM_008767	Mus musculus solute carrier family 22 (organic cation transporter), member 18 (Slc22a18), transcript variant 1, mRNA [NM_008767]
A_51_P372826	3.7735744	up	NM_145533	Smox	NM_145533	Mus musculus spermine oxidase (Smox), mRNA [NM_145533]
A_52_P430194	5.8303814	up	AK087437	Smox	AK087437	Mus musculus 0 day neonate eyeball cDNA, RIKEN full-length enriched library, clone:E130115D16 product:POLYAMINE OXIDASE ISOFORM-3 homolog [Homo sapiens], full insert sequence. [AK087437]
A_51_P474459	5.173515	up	NM_007707	Socs3	NM_007707	Mus musculus suppressor of cytokine signaling 3 (Socs3), mRNA [NM_007707]
A_52_P204311	9.630001	up	NM_011898	Spry4	NM_011898	Mus musculus sprouty homolog 4 (Drosophila) (Spry4), mRNA [NM_011898]
A_51_P390715	5.047894	up	NM_011577	Tgfb1	NM_011577	Mus musculus transforming growth factor, beta 1 (Tgfb1), mRNA [NM_011577]
A_52_P337092	2.0655103	up	NM_011580	Thbs1	NM_011580	Mus musculus thrombospondin 1 (Thbs1), mRNA [NM_011580]
A_52_P337086	4.5024614	up	NM_011580	Thbs1	NM_011580	Mus musculus thrombospondin 1 (Thbs1), mRNA [NM_011580]
A_52_P363951	4.5403414	up	NM_011580	Thbs1	NM_011580	Mus musculus thrombospondin 1 (Thbs1), mRNA [NM_011580]
A_51_P388835	2.7979538	down	NM_009380	Thrb	NM_009380	Mus musculus thyroid hormone receptor beta (Thrb), mRNA [NM_009380]
A_52_P532559	3.1085851	down	AK158826	Thrb	AK158826	Mus musculus visual cortex cDNA, RIKEN full-length enriched library, clone:K430339A03 product:thyroid hormone receptor beta, full insert sequence. [AK158826]
A_52_P877015	3.785894	down	BC089035	Thrb	BC089035	Mus musculus thyroid hormone receptor beta, mRNA (cDNA clone MGC:115768 IMAGE:30695885), complete cds [BC089035]
A_51_P313532	1.9120182	down	NM_027154	Tmbim1	NM_027154	Mus musculus transmembrane BAX inhibitor motif containing 1 (Tmbim1), mRNA [NM_027154]
A_52_P461378	2.0281549	down	NM_027154	Tmbim1	NM_027154	Mus musculus transmembrane BAX inhibitor motif containing 1 (Tmbim1), mRNA [NM_027154]
A_52_P127362	1.5548475	down	NM_029478	Tmem49	NM_029478	Mus musculus transmembrane protein 49 (Tmem49), mRNA [NM_029478]
A_52_P413448	2.1169481	down	AK019443	Tmem49	AK019443	Mus musculus 13 days embryo head cDNA, RIKEN full-length enriched library, clone:3110098I04 product:unclassifiable, full insert sequence. [AK019443]
A_51_P439746	4.2890396	down	NM_029478	Tmem49	NM_029478	Mus musculus transmembrane protein 49 (Tmem49), mRNA [NM_029478]
A_51_P278282	1.5826579	down	NM_011610	Tnfrsf1b	NM_011610	Mus musculus tumor necrosis factor receptor superfamily, member 1b (Tnfrsf1b), mRNA [NM_011610]
A_52_P228621	2.2646317	up	NM_011610	Tnfrsf1b	NM_011610	Mus musculus tumor necrosis factor receptor superfamily, member 1b (Tnfrsf1b), mRNA [NM_011610]
A_52_P151853	2.8686774	down	NM_009412	Tpd52	NM_009412	Mus musculus tumor protein D52 (Tpd52), transcript variant 5, mRNA [NM_009412]
A_51_P506792	3.289838	down	NM_009412	Tpd52	NM_009412	Mus musculus tumor protein D52 (Tpd52), transcript variant 5, mRNA [NM_009412]
A_52_P383913	3.6701207	up	AK020775	Trim35	AK020775	Mus musculus 0 day neonate thymus cDNA, RIKEN full-length enriched library, clone:A430106H13 product:tripartite motif-containing 35, full insert sequence. [AK020775]
A_52_P45738	5.755788	up	NM_029979	Trim35	NM_029979	Mus musculus tripartite motif-containing 35 (Trim35), mRNA [NM_029979]
A_51_P373777	6.833079	up	NM_029979	Trim35	NM_029979	Mus musculus tripartite motif-containing 35 (Trim35), mRNA [NM_029979]
A_51_P173656	3.2427914	up	NM_134028	Tubg2	NM_134028	Mus musculus tubulin, gamma 2 (Tubg2), mRNA [NM_134028]
A_51_P109840	3.8461542	down	NM_011707	Vtn	NM_011707	Mus musculus vitronectin (Vtn), mRNA [NM_011707]
A_51_P390804	2.9994416	up	NM_016873	Wisp2	NM_016873	Mus musculus WNT1 inducible signaling pathway protein 2 (Wisp2), mRNA [NM_016873]
A_51_P121206	4.979566	up	NM_015753	Zeb2	NM_015753	Mus musculus zinc finger E-box binding homeobox 2 (Zeb2), transcript variant 2, mRNA [NM_015753]

A_52_P526372	6.219306	up	NM_015753	Zeb2	NM_015753	Mus musculus zinc finger E-box binding homeobox 2 (Zeb2), transcript variant 2, mRNA [NM_015753]
A_52_P596008	6.952508	up	NM_015753	Zeb2	NM_015753	Mus musculus zinc finger E-box binding homeobox 2 (Zeb2), transcript variant 2, mRNA [NM_015753]
A_51_P106893	7.538661	up	AK051204	Zeb2	AK051204	Mus musculus 12 days embryo spinal ganglion cDNA, RIKEN full-length enriched library, clone:D130016B08 product:unclassifiable, full insert sequence. [AK051204]
A_52_P587928	8.078221	up	AK031541	Zeb2	AK031541	Mus musculus 13 days embryo male testis cDNA, RIKEN full-length enriched library, clone:6030449F06 product:zinc finger homeobox 1b, full insert sequence. [AK031541]

List of cancer genes that were up or down regulated in two tumor samples with respect to their normal controls (genes that were affected at least by 2 fold with p value ≤ 0.01 were listed here)
Genes that exhibited at least by 2 fold with p value £ 0.01 were listed here
