# Supplementary material for: Predisposition to Cancer Caused by Genetic and Functional Defects of Mammalian Atad5
Source: PLoS Genet. 2011 Aug 25;7(8):e1002245. doi: 10.1371/journal.pgen.1002245 (PMC3161924; doi:10.1371/journal.pgen.1002245)
Supplement: Table S3 — Genes that showed both altered expression and copy number in murine tumors. (PDF) [file pgen.1002245.s007.pdf]

Table S3. Genes that showed both altered expression and copy number in murine tumors.

Down regulated and deleted

ProbeName	Fold chang	Regulation(	Common nar	GeneSymb	Description	GenbankAccession
A_51_P336282	3.319486	down	NM_010934	Npy1r	Mus musculus neuropeptide Y receptor Y1 (Npy1r), mRNA [NM_010934]	NM_010934
A_51_P390884	2.516718	down	NM_011862	Pacsin2	Mus musculus protein kinase C and casein kinase substrate in neurons 2 (Pacsin2), mRNA [NM_011862]	NM_011862
A_52_P229648	2.598706	down	NM_011862	Pacsin2	Mus musculus protein kinase C and casein kinase substrate in neurons 2 (Pacsin2), mRNA [NM_011862]	NM_011862
A_52_P407941	1.48456	down	NM_172125	Adam1b	Mus musculus a disintegrin and metallopeptidase domain 1b (Adam1b), mRNA [NM_172125]	NM_172125
A_51_P381670	1.249522	down	NM_023066	Asph	Mus musculus aspartate-beta-hydroxylase (Asph), transcript variant 1, mRNA [NM_023066]	NM_023066
A_51_P253531	3.800577	down	NM_026097	Rffl	Mus musculus ring finger and FYVE like domain containing protein (Rffl), transcript variant 2, mRNA [NM_026097]	NM_026097
A_52_P356698	1.938858	down	NM_013665	Shox2	Mus musculus short stature homeobox 2 (Shox2), mRNA [NM_013665]	NM_013665
A_52_P399175	2.815641	down	NM_001007	Rffl	Mus musculus ring finger and FYVE like domain containing protein (Rffl), transcript variant 1, mRNA [NM_001007465]	NM_001007465
A_51_P346842	1.528089	down	NM_001081	Pcdh11x	Mus musculus protocadherin 11 X-linked (Pcdh11x), mRNA [NM_001081385]	NM_001081385
A_52_P545393	2.563429	down	NM_011862	Pacsin2	Mus musculus protein kinase C and casein kinase substrate in neurons 2 (Pacsin2), mRNA [NM_011862]	NM_011862
A_52_P625171	1.775249	down	NM_133723	Asph	Mus musculus aspartate-beta-hydroxylase (Asph), transcript variant 2, mRNA [NM_133723]	NM_133723
A_52_P679869	1.037685	down	NM_001081	Pcdh11x	Mus musculus protocadherin 11 X-linked (Pcdh11x), mRNA [NM_001081385]	NM_001081385
A_51_P344447	5.632451	down	NM_172125	Adam1b	Mus musculus a disintegrin and metallopeptidase domain 1b (Adam1b), mRNA [NM_172125]	NM_172125
A_52_P124219	1.485787	down	AK054261	Pot1b	Mus musculus 2 days pregnant adult female ovary cDNA, RIKEN full-length enriched library, clone:E330005B05 product:	AK054261
A_51_P253527	3.733293	down	NM_026097	Rffl	Mus musculus ring finger and FYVE like domain containing protein (Rffl), transcript variant 2, mRNA [NM_026097]	NM_026097
A_51_P453079	1.52097	down	NM_148943	Usp9y	Mus musculus ubiquitin specific peptidase 9, Y chromosome (Usp9y), mRNA [NM_148943]	NM_148943
A_52_P306351	1.910277	down	AK165260	Asph	Mus musculus 6 days neonate spleen cDNA, RIKEN full-length enriched library, clone:F430004K08 product:aspartate-be	AK165260

Upregulated and amplified

ProbeName	Fold chang	Regulation(	Common nar	GeneSymb	Description	GenbankAccession
A_51_P140321	1.415056	up	NM_026779	Mocos	Mus musculus molybdenum cofactor sulfurase (Mocos), mRNA [NM_026779]	NM_026779
A_52_P75348	4.343492	up	NM_027411	Ccdc99	Mus musculus coiled-coil domain containing 99 (Ccdc99), mRNA [NM_027411]	NM_027411
A_52_P622600	1.189097	up	AK013536	3010026O	Mus musculus adult male hippocampus cDNA, RIKEN full-length enriched library, clone:2900016G09 product:hypothetic	AK013536
A_51_P415126	2.26505	up	NM_031199	Tgfa	Mus musculus transforming growth factor alpha (Tgfa), mRNA [NM_031199]	NM_031199
A_51_P454993	1.637344	up	NM_178874	Tmcc2	Mus musculus transmembrane and coiled-coil domains 2 (Tmcc2), mRNA [NM_178874]	NM_178874
A_52_P231217	1.263406	up	AK050320	Nfia	Mus musculus adult male liver tumor cDNA, RIKEN full-length enriched library, clone:C730037C02 product:nuclear facto	AK050320
A_52_P633550	2.851515	up	AK053579	Ghr	Mus musculus 0 day neonate eyeball cDNA, RIKEN full-length enriched library, clone:E130111P21 product:growth horm	AK053579
A_51_P121302	1.173367	up	NM_029431	Them4	Mus musculus thioesterase superfamily member 4 (Them4), mRNA [NM_029431]	NM_029431
A_52_P661044	1.471235	up	NM_009914	Ccr3	Mus musculus chemokine (C-C motif) receptor 3 (Ccr3), mRNA [NM_009914]	NM_009914
A_52_P101496	1.413626	up	BC002033	Rad50	Mus musculus RAD50 homolog (S. cerevisiae), mRNA (cDNA clone IMAGE:3491597), complete cds. [BC002033]	BC002033
A_51_P508853	1.73272	up	AK030095	Ep400	Mus musculus adult male testis cDNA, RIKEN full-length enriched library, clone:4932441J05 product:similar to P400 SWI	AK030095
A_51_P427317	1.15792	up	NM_013855	Abca3	Mus musculus ATP-binding cassette, sub-family A (ABC1), member 3 (Abca3), transcript variant 1, mRNA [NM_013855]	NM_013855
A_52_P84315	1.275373	up	NM_001081	Chd7	Mus musculus chromodomain helicase DNA binding protein 7 (Chd7), mRNA [NM_001081417]	NM_001081417
A_51_P290840	14.96621	up	NM_172475	Frmd4a	Mus musculus FERM domain containing 4A (Frmd4a), mRNA [NM_172475]	NM_172475
A_52_P556933	6.441222	up	NM_028266	Col16a1	Mus musculus procollagen, type XVI, alpha 1 (Col16a1), mRNA [NM_028266]	NM_028266
A_51_P499854	1.748589	up	NM_010284	Ghr	Mus musculus growth hormone receptor (Ghr), transcript variant 1, mRNA [NM_010284]	NM_010284
A_51_P450057	1.122329	up	NM_007708	Cit	Mus musculus citron (Cit), mRNA [NM_007708]	NM_007708
A_52_P618132	1.17909	up	NM_172865	Manea	Mus musculus mannosidase, endo-alpha (Manea), mRNA [NM_172865]	NM_172865
A_51_P211934	1.588249	up	NM_138593	Larp7	Mus musculus La ribonucleoprotein domain family, member 7 (Larp7), mRNA [NM_138593]	NM_138593
A_52_P218132	2.112158	up	AK083930	Top1	Mus musculus 12 days embryo spinal ganglion cDNA, RIKEN full-length enriched library, clone:D130064I21 product:topo	AK083930
A_52_P265996	1.430908	up	AK031375	Odz4	Mus musculus 13 days embryo male testis cDNA, RIKEN full-length enriched library, clone:6030416D17 product:odd Oz/	AK031375
A_52_P379531	4.835554	up	NM_011589	Timeless	Mus musculus timeless homolog (Drosophila) (Timeless), mRNA [NM_011589]	NM_011589
A_52_P374997	9.670921	up	NM_178609	E2f7	Mus musculus E2F transcription factor 7 (E2f7), mRNA [NM_178609]	NM_178609
A_52_P249588	1.084398	up	NM_028043	D1Bwg021	Mus musculus DNA segment, Chr 1, Brigham & Women's Genetics 0212 expressed (D1Bwg0212e), mRNA [NM_028043]	NM_028043
A_52_P233255	3.062366	up	NM_053171	Csmd1	Mus musculus CUB and Sushi multiple domains 1 (Csmd1), mRNA [NM_053171]	NM_053171
A_52_P530121	1.38253	up	NM_001081	D430042O	Mus musculus RIKEN cDNA D430042O09 gene (D430042O09Rik), mRNA [NM_001081022]	NM_001081022
A_52_P680935	1.489004	up	NM_010284	Ghr	Mus musculus growth hormone receptor (Ghr), transcript variant 1, mRNA [NM_010284]	NM_010284
A_52_P597800	2.086194	up	NM_007961	Etv6	Mus musculus ets variant gene 6 (TEL oncogene) (Etv6), mRNA [NM_007961]	NM_007961
A_52_P191193	1.762605	up	NM_172595	Arl15	Mus musculus ADP-ribosylation factor-like 15 (Arl15), mRNA [NM_172595]	NM_172595

A_52_P421149	1.403726	up	XM_204015	Rere	PREDICTED: Mus musculus arginine glutamic acid dipeptide (RE) repeats, transcript variant 1 (Rere), mRNA [XM_204015	XM_204015
A_52_P108021	1.266896	up	NM_00108	D430042O(	Mus musculus RIKEN cDNA D430042O09 gene (D430042O09Rik), mRNA [NM_001081022]	NM_001081022
A_52_P234214	1.6712	up	AK083774	Manea	Mus musculus 12 days embryo spinal ganglion cDNA, RIKEN full-length enriched library, clone:D130006M08 product:un	AK083774
A_52_P91563	1.175985	up	NM_008070	Gabrb2	Mus musculus gamma-aminobutyric acid (GABA-A) receptor, subunit beta 2 (Gabrb2), mRNA [NM_008070]	NM_008070
A_52_P446677	1.724954	up	NM_183163	Rhbdl2	Mus musculus rhomboid, veinlet-like 2 (Drosophila) (Rhbdl2), mRNA [NM_183163]	NM_183163
A_51_P237878	2.971797	up	NM_027671	Sntg1	Mus musculus syntrophin, gamma 1 (Sntg1), mRNA [NM_027671]	NM_027671
A_52_P516733	1.05975	up	NM_145959	D15Ertd62	Mus musculus DNA segment, Chr 15, ERATO Doi 621, expressed (D15Ertd621e), mRNA [NM_145959]	NM_145959
A_51_P155445	1.096393	up	NM_011076	Abcb1a	Mus musculus ATP-binding cassette, sub-family B (MDR/TAP), member 1A (Abcb1a), mRNA [NM_011076]	NM_011076
A_52_P360529	27.06769	up	AK089210	Frmd4a	Mus musculus NOD-derived CD11c +ve dendritic cells cDNA, RIKEN full-length enriched library, clone:F630038O13 produ	AK089210
A_52_P278238	6.844258	up	AK016937	Cep170	Mus musculus adult male testis cDNA, RIKEN full-length enriched library, clone:4933426L22 product:KARP-1-BINDING P	AK016937
A_52_P100506	1.167095	up	NM_145959	D15Ertd62	Mus musculus DNA segment, Chr 15, ERATO Doi 621, expressed (D15Ertd621e), mRNA [NM_145959]	NM_145959
A_52_P555423	2.31139	up	NM_031199	Tgfa	Mus musculus transforming growth factor alpha (Tgfa), mRNA [NM_031199]	NM_031199
A_51_P239884	1.014231	up	NM_172865	Manea	Mus musculus mannosidase, endo-alpha (Manea), mRNA [NM_172865]	NM_172865
A_52_P451123	1.155173	up	AK044403	1110003E0	Mus musculus adult retina cDNA, RIKEN full-length enriched library, clone:A930011B09 product:unclassifiable, full inser	AK044403
A_51_P489034	1.437364	up	NM_177694	Tmem16e	Mus musculus transmembrane protein 16E (Tmem16e), mRNA [NM_177694]	NM_177694
A_52_P84217	1.172637	up	AK077982	Ophn1	Mus musculus 13 days embryo male testis cDNA, RIKEN full-length enriched library, clone:6030495I02 product:oligophr	AK077982
A_51_P479138	1.704777	up	NM_146547	Olfr771	Mus musculus olfactory receptor 771 (Olfr771), mRNA [NM_146547]	NM_146547
A_51_P396570	5.17464	up	NM_011961	Plod2	Mus musculus procollagen lysine, 2-oxoglutarate 5-dioxygenase 2 (Plod2), mRNA [NM_011961]	NM_011961
A_52_P175821	1.123003	up	NM_00108	Chd7	Mus musculus chromodomain helicase DNA binding protein 7 (Chd7), mRNA [NM_001081417]	NM_001081417
A_52_P117294	2.122613	up	NM_177136	Fryl	Mus musculus furry homolog-like (Drosophila) (Fryl), transcript variant 2, mRNA [NM_177136]	NM_177136
A_51_P380165	1.126158	up	NM_029337	Ep400	Mus musculus E1A binding protein p400 (Ep400), mRNA [NM_029337]	NM_029337
A_51_P432764	2.874419	up	NM_007547	Sirpa	Mus musculus signal-regulatory protein alpha (Sirpa), mRNA [NM_007547]	NM_007547
A_51_P296013	2.592618	up	NM_007547	Sirpa	Mus musculus signal-regulatory protein alpha (Sirpa), mRNA [NM_007547]	NM_007547
A_51_P515446	1.014488	up	NM_018887	Cyp39a1	Mus musculus cytochrome P450, family 39, subfamily a, polypeptide 1 (Cyp39a1), mRNA [NM_018887]	NM_018887
A_51_P137094	11.99095	up	NM_015772	Sall2	Mus musculus sal-like 2 (Drosophila) (Sall2), mRNA [NM_015772]	NM_015772
A_52_P491861	1.455853	up	NM_009282	Stag1	Mus musculus stromal antigen 1 (Stag1), mRNA [NM_009282]	NM_009282
A_51_P155152	1.781355	up	NM_020332	Ank	Mus musculus progressive ankylosis (Ank), mRNA [NM_020332]	NM_020332
A_52_P432289	1.877695	up	NM_023066	Asph	Mus musculus aspartate-beta-hydroxylase (Asph), transcript variant 1, mRNA [NM_023066]	NM_023066
A_52_P460687	1.01217	up	NM_008070	Gabrb2	Mus musculus gamma-aminobutyric acid (GABA-A) receptor, subunit beta 2 (Gabrb2), mRNA [NM_008070]	NM_008070
A_51_P464539	3.448329	up	NM_153412	Phldb2	Mus musculus pleckstrin homology-like domain, family B, member 2 (Phldb2), mRNA [NM_153412]	NM_153412
A_51_P118468	4.105514	up	AK039235	Cep170	Mus musculus adult male spinal cord cDNA, RIKEN full-length enriched library, clone:A330004A13 product:hypothetical	AK039235
A_51_P507307	1.081933	up	NM_00108	Chd7	Mus musculus chromodomain helicase DNA binding protein 7 (Chd7), mRNA [NM_001081417]	NM_001081417
A_52_P589622	12.34876	up	NM_008651	Mybl1	Mus musculus myeloblastosis oncogene-like 1 (Mybl1), mRNA [NM_008651]	NM_008651
A_52_P640686	5.740226	up	AF086824	Cit	Mus musculus rho/rac-interacting citron kinase (Crik) mRNA, complete cds. [AF086824]	AF086824
A_52_P1115466	1.115215	up	AK020879	Eif4enif1	Mus musculus adult retina cDNA, RIKEN full-length enriched library, clone:A930019J01 product:unclassifiable, full insert	AK020879
A_52_P598774	1.358978	up	NM_00100	Baz2b	Mus musculus bromodomain adjacent to zinc finger domain, 2B (Baz2b), mRNA [NM_001001182]	NM_001001182
A_51_P194249	1.842856	up	NM_019675	Stmn4	Mus musculus stathmin-like 4 (Stmn4), mRNA [NM_019675]	NM_019675
A_51_P429592	4.79948	up	NM_053171	Csmd1	Mus musculus CUB and Sushi multiple domains 1 (Csmd1), mRNA [NM_053171]	NM_053171
A_51_P357486	1.622504	up	NM_138593	Larp7	Mus musculus La ribonucleoprotein domain family, member 7 (Larp7), mRNA [NM_138593]	NM_138593
A_52_P533540	5.893283	up	ENSMUST00	Cep170	centrosomal protein 170 [Source:MarkerSymbol;Acc:MGI:1918348] [ENSMUST00000070119]	
A_51_P356931	3.865885	up	NM_011015	Orc1l	Mus musculus origin recognition complex, subunit 1-like (S.cereviaiae) (Orc1l), transcript variant A, mRNA [NM_011015	NM_011015
A_51_P185071	1.127101	up	NM_146332	Olfr135	Mus musculus olfactory receptor 135 (Olfr135), mRNA [NM_146332]	NM_146332
A_52_P267451	1.272236	up	NM_009282	Stag1	Mus musculus stromal antigen 1 (Stag1), mRNA [NM_009282]	NM_009282
A_51_P187352	2.298994	up	NM_010610	Kcnma1	Mus musculus potassium large conductance calcium-activated channel, subfamily M, alpha member 1 (Kcnma1), mRNA	NM_010610
A_51_P433887	1.277407	up	NM_011720	Wnt8b	Mus musculus wingless related MMTV integration site 8b (Wnt8b), mRNA [NM_011720]	NM_011720
A_52_P125485	2.293351	up	NM_177809	AU042651	Mus musculus expressed sequence AU042651 (AU042651), mRNA [NM_177809]	NM_177809
A_51_P450549	1.556345	up	NM_011060	Padi3	Mus musculus peptidyl arginine deiminase, type III (Padi3), mRNA [NM_011060]	NM_011060
A_52_P631240	2.039531	up	AK029073	Frmd4a	Mus musculus 10 days neonate skin cDNA, RIKEN full-length enriched library, clone:4732488M06 product:similar to HYP	AK029073
A_52_P394755	1.015785	up	NM_152825	Usp45	Mus musculus ubiquitin specific petidase 45 (Usp45), mRNA [NM_152825]	NM_152825
A_51_P394745	1.850025	up	AK009138	Fryl	Mus musculus adult male tongue cDNA, RIKEN full-length enriched library, clone:2310004H21 product:hypothetical pro	AK009138
A_51_P161037	6.654703	up	AK172953	Cep170	Mus musculus mRNA for mKIAA0470 protein. [AK172953]	AK172953
A_51_P241210	13.08977	up	NM_00108	Lhfpl3	Mus musculus lipoma HMGIC fusion partner-like 3 (Lhfpl3), mRNA [NM_001081231]	NM_001081231

A_52_P477559	9.742324	up	NM_172475	Frmd4a	Mus musculus FERM domain containing 4A (Frmd4a), mRNA [NM_172475]	NM_172475
A_52_P680941	1.508557	up	NM_010284	Ghr	Mus musculus growth hormone receptor (Ghr), transcript variant 1, mRNA [NM_010284]	NM_010284
A_51_P227564	3.707463	up	NM_027411	Ccdc99	Mus musculus coiled-coil domain containing 99 (Ccdc99), mRNA [NM_027411]	NM_027411
A_52_P393488	2.291699	up	NM_029499	Ms4a4c	Mus musculus membrane-spanning 4-domains, subfamily A, member 4C (Ms4a4c), mRNA [NM_029499]	NM_029499
A_52_P86935	1.435047	up	NM_026779	Mocos	Mus musculus molybdenum cofactor sulfurase (Mocos), mRNA [NM_026779]	NM_026779
A_52_P170635	3.247001	up	NM_007547	Sirpa	Mus musculus signal-regulatory protein alpha (Sirpa), mRNA [NM_007547]	NM_007547
A_51_P412768	1.459004	up	NM_009914	Ccr3	Mus musculus chemokine (C-C motif) receptor 3 (Ccr3), mRNA [NM_009914]	NM_009914
A_51_P437559	3.512431	up	NM_011589	Timeless	Mus musculus timeless homolog (Drosophila) (Timeless), mRNA [NM_011589]	NM_011589
A_51_P230287	3.268854	up	NM_030889	Sorcs2	Mus musculus sortilin-related VPS10 domain containing receptor 2 (Sorcs2), mRNA [NM_030889]	NM_030889
A_52_P403420	1.433174	up	AK081492	Atp6v1h	Mus musculus 16 days embryo head cDNA, RIKEN full-length enriched library, clone:C130021C16 product:hypothetical V	AK081492
A_51_P317460	4.628732	up	NM_010824	Mpo	Mus musculus myeloperoxidase (Mpo), mRNA [NM_010824]	NM_010824
A_52_P108023	1.12902	up	NM_001081(	D430042O(	Mus musculus RIKEN cDNA D430042O09 gene (D430042O09Rik), mRNA [NM_001081022]	NM_001081022
A_52_P544052	1.364355	up	NM_0010011	Baz2b	Mus musculus bromodomain adjacent to zinc finger domain, 2B (Baz2b), mRNA [NM_001001182]	NM_001001182
A_52_P404469	2.665476	up	AK162292	Rhbdl2	Mus musculus adult male colon cDNA, RIKEN full-length enriched library, clone:9030006F14 product:hypothetical protei	AK162292
A_52_P475229	1.203776	up	NM_029337	Ep400	Mus musculus E1A binding protein p400 (Ep400), mRNA [NM_029337]	NM_029337
A_52_P619991	1.631913	up	AK014834	Them4	Mus musculus adult male testis cDNA, RIKEN full-length enriched library, clone:4921507I02 product:weakly similar to C-	AK014834
A_52_P279845	2.953864	up	AK047881	Sorcs2	Mus musculus 16 days embryo head cDNA, RIKEN full-length enriched library, clone:C130019G06 product:VPS10 domai	AK047881
A_51_P223092	1.346762	up	NM_028106	Zbed3	Mus musculus zinc finger, BED domain containing 3 (Zbed3), mRNA [NM_028106]	NM_028106
A_51_P103237	5.725899	up	NM_015755	Hunk	Mus musculus hormonally upregulated Neu-associated kinase (Hunk), mRNA [NM_015755]	NM_015755
A_51_P444666	1.462685	up	NM_144830	Tmem106a	Mus musculus transmembrane protein 106A (Tmem106a), mRNA [NM_144830]	NM_144830
A_51_P291062	9.097803	up	NM_028266	Col16a1	Mus musculus procollagen, type XVI, alpha 1 (Col16a1), mRNA [NM_028266]	NM_028266
A_52_P673971	1.872442	up	NM_009282	Stag1	Mus musculus stromal antigen 1 (Stag1), mRNA [NM_009282]	NM_009282
A_52_P194382	2.939632	up	NM_025850	Fank1	Mus musculus fibronectin type 3 and ankyrin repeat domains 1 (Fank1), mRNA [NM_025850]	NM_025850
A_52_P153700	2.833047	up	NM_153412	Phldb2	Mus musculus pleckstrin homology-like domain, family B, member 2 (Phldb2), mRNA [NM_153412]	NM_153412
A_52_P203741	3.366654	up	NM_009176	St3gal3	Mus musculus ST3 beta-galactoside alpha-2,3-sialyltransferase 3 (St3gal3), mRNA [NM_009176]	NM_009176
A_52_P155692	5.477172	up	NM_009176	St3gal3	Mus musculus ST3 beta-galactoside alpha-2,3-sialyltransferase 3 (St3gal3), mRNA [NM_009176]	NM_009176
A_51_P111462	1.792434	up	NM_172595	Arl15	Mus musculus ADP-ribosylation factor-like 15 (Arl15), mRNA [NM_172595]	NM_172595
A_51_P446022	1.368964	up	AK052528	D430042O(	Mus musculus 13 days embryo lung cDNA, RIKEN full-length enriched library, clone:D430042O09 product:hypothetical p	AK052528
A_51_P240019	1.227721	up	NM_013755	Gyg	Mus musculus glycogenin (Gyg), mRNA [NM_013755]	NM_013755
A_52_P670950	1.175861	up	NM_029337	Ep400	Mus musculus E1A binding protein p400 (Ep400), mRNA [NM_029337]	NM_029337
A_52_P54106	2.634995	up	AK034547	Ghr	Mus musculus 12 days embryo embryonic body between diaphragm region and neck cDNA, RIKEN full-length enriched l	AK034547
A_51_P356552	1.442655	up	NM_007826	Dach1	Mus musculus dachshund 1 (Drosophila) (Dach1), transcript variant 1, mRNA [NM_007826]	NM_007826
A_52_P636649	1.982663	up	AK048713	Csmd1	Mus musculus 0 day neonate cerebellum cDNA, RIKEN full-length enriched library, clone:C230015P12 product:unclassifi	AK048713
A_51_P283044	1.618325	up	NM_011419	Jarid1d	Mus musculus jumonji, AT rich interactive domain 1D (Rbp2 like) (Jarid1d), mRNA [NM_011419]	NM_011419
A_51_P444669	1.427282	up	NM_144830	Tmem106a	Mus musculus transmembrane protein 106A (Tmem106a), mRNA [NM_144830]	NM_144830
A_52_P541833	1.475532	up	NM_177876	Vps37b	Mus musculus vacuolar protein sorting 37B (yeast) (Vps37b), mRNA [NM_177876]	NM_177876
A_52_P479539	5.842863	up	AK047894	Cit	Mus musculus 16 days embryo head cDNA, RIKEN full-length enriched library, clone:C130020H05 product:unclassifiable	AK047894
A_51_P139184	1.884928	up	NM_025850	Fank1	Mus musculus fibronectin type 3 and ankyrin repeat domains 1 (Fank1), mRNA [NM_025850]	NM_025850
A_52_P418515	2.255601	up	NM_001024(	1700007G1	Mus musculus RIKEN cDNA 1700007G11 gene (1700007G11Rik), mRNA [NM_001024614]	NM_001024614
A_52_P458853	1.439761	up	AK012459	Brf2	Mus musculus 11 days embryo whole body cDNA, RIKEN full-length enriched library, clone:2700059M06 product:BRF2,	AK012459
A_52_P267446	1.401976	up	NM_009282	Stag1	Mus musculus stromal antigen 1 (Stag1), mRNA [NM_009282]	NM_009282
A_52_P607075	8.226221	up	AK041481	E2f7	Mus musculus 3 days neonate thymus cDNA, RIKEN full-length enriched library, clone:A630014C11 product:hypothetica	AK041481
A_52_P100028	1.526413	up	NM_009035	Rbpj	Mus musculus recombination signal binding protein for immunoglobulin kappa J region (Rbpj), transcript variant 1, mRN	NM_009035
A_51_P280013	2.344587	up	NM_028194	Fryl	Mus musculus furry homolog-like (Drosophila) (Fryl), transcript variant 1, mRNA [NM_028194]	NM_028194
A_52_P309022	1.292373	up	NM_007826	Dach1	Mus musculus dachshund 1 (Drosophila) (Dach1), transcript variant 1, mRNA [NM_007826]	NM_007826

List of genes identified both from the microarray due to expression change and the comparative genomic hybridization analyses as gain or loss
